# Evaluating large language model performance in Risk of Bias assessments: A cross-sectional validation study

**DOI:** 10.1371/journal.pone.0353155

**Published:** 2026-07-09

**Authors:** Siddharth Gandhi, Arveen Shokravi, Yashan Chelliahpillai, Michael Balas

**Affiliations:** 1 Faculty of Medicine, Queen’s University, Kingston, Ontario, Canada; 2 Department of Medicine, University of Calgary, Calgary, Alberta, Canada; 3 Department of Medicine, University of Toronto, Toronto, Ontario, Canada; 4 Department of Ophthalmology and Vision Sciences, University of Toronto, Toronto, Ontario, Canada; The University of Alabama in Huntsville, UNITED STATES OF AMERICA

## Abstract

**Objective:**

To evaluate the reliability and diagnostic performance of ChatGPT-o3 in conducting Risk of Bias (RoB) assessments of randomized clinical trials (RCTs) using the Cochrane RoB 2.0 tool.

**Materials and methods:**

This methodological validation study analyzed 50 RCTs sampled from 50 published meta-analyses. Each trial was independently assessed by the original systematic review authors (OSRAs), our masked human panel, and ChatGPT-o3. Structured prompts based on RoB 2.0 guidelines were used to elicit ChatGPT-o3 assessments. Agreement was evaluated using weighted Cohen’s kappa and Gwet’s AC_2_. Diagnostic performance was measured by sensitivity, specificity, and balanced accuracy, with human ratings as the reference.

**Results:**

ChatGPT-o3 classified 34% of trials as high risk, compared with 22% by our panel, and 12% by the OSRAs. Agreement was modest (median κ: 0.33 with our panel; 0.14 with OSRAs). Overall Gwet’s AC_2_ was 0.30. For detecting high-risk trials, ChatGPT-o3 achieved a sensitivity of 0.46, specificity of 0.69, and balanced accuracy of 0.57. For low-risk trials, its sensitivity was 0.47, specificity was 0.86, and balanced accuracy was 0.66.

**Discussion:**

The results indicate that ChatGPT-o3 produced more conservative RoB ratings than human reviewers, identifying a greater percentage of trials as having a high RoB.

**Conclusion:**

While unsuitable to be used as a sole assessor, ChatGPT-o3 may serve as an adjunct tool to enhance the efficiency and consistency of RoB assessments in systematic reviews.

## Introduction

Accurate Risk of Bias (RoB) assessments in randomized clinical trials (RCTs) are essential for producing high-quality systematic reviews, which directly inform clinical guidelines and healthcare decisions [1–3]. Although widely accepted, tools like the Cochrane RoB 2 tool remain labor-intensive and subject to variability between reviewers, posing challenges as the volume of biomedical literature grows rapidly [4,5].

Large language models (LLMs), including ChatGPT, have emerged as potential tools to automate critical components of evidence synthesis, such as RoB assessment [6–8]. Previous evaluations utilizing simpler, checklist-based instruments have reported substantial agreement between LLMs and human reviewers. For instance, Lai et al. (2024) demonstrated that LLMs achieved accuracy rates exceeding 85% when assessing bias using a modified version of the original Cochrane tool (the CLARITY instrument), which relies on explicit text extraction [9]. Similarly, Xia et al. (2025) reported high performance when applying LLMs to cohort studies using the Newcastle-Ottawa Scale, suggesting that AI models perform well with explicit criteria [10]. However, the transition to complex, domain-based tools like RoB 2.0 has challenged AI reasoning capabilities. Šuster et al. (2024) found that zero-shot prompting of LLMs on RoB 2.0 yielded poor performance, while Huang et al. (2025) noted that accuracy for domain-level judgments dropped to between 57% and 70% [11,12] This struggle extends to non-randomized studies as well; Hasan et al. recently reported only moderate agreement (61%) between GPT-4 and humans using the ROBINS-I tool [13]. Ultimately, early research suggests that these models can achieve good agreement with expert assessments, indicating their promise for enhancing efficiency and consistency in systematic reviews [9,12,14,15]. However, existing evidence is limited by small, narrowly selected datasets and reliance on older model versions [9,16]. There is also a need to further assess these LLMs with the latest RoB tools.

The present study evaluates the performance of a recent, advanced LLM (ChatGPT-o3) for RoB assessments across a broad and diverse sample of 50 randomly selected RCTs from various medical specialties [17]. We aimed to quantify the agreement between LLM-generated assessments and blinded human expert ratings, providing essential evidence regarding the potential of LLMs as reliable adjunctive tools for systematic review methodologies.

By clarifying the capabilities and limitations of state-of-the-art LLMs in conducting RoB evaluations, this study seeks to inform future directions in automated evidence synthesis. These advancements could streamline the systematic review process and help ensure faster, more reproducible, and methodologically rigorous integration of clinical evidence into practice.

## Methods

### Study design and search strategy

This methodological validation study aimed to quantify the reliability and accuracy of an advanced LLM (ChatGPT-o3; OpenAI, San Francisco, CA, USA) in performing RoB assessments for RCTs [18]. The reporting of this study follows the Transparent Reporting of a multivariable prediction model for Individual Prognosis Or Diagnosis-Large Language Models (TRIPOD-LLM) guidelines [19]. We conducted a systematic search on PubMed using the query: “clinical trial*” AND “meta analysis” AND “Risk of Bias 2”. This specific search strategy was used to identify studies that employed the revised Cochrane RoB 2.0 tool, as it is the most widely adopted instrument for assessing RoB in RCTs [20] A formal study protocol was not pre-registered for this methodological validation. The search yielded meta-analyses published between 2020 and 2025. From the resulting records, we randomly selected 50 meta-analyses to ensure a diverse sample for interrater agreement analysis. Each meta-analysis was manually screened to ensure that complete RoB assessments were included. Subsequently, from each meta-analysis, we randomly selected one RCT using a random number generator based on the total number of RCTs assessed in that review, resulting in a sample of 50 RCTs across diverse medical subspecialties (SMethods 1 in S1 File). The publication dates of the 50 randomly selected RCTs ranged from 1998 to 2025. Because no human participants or data were involved, institutional review board approval was not required. The study adhered to the tenets of the Declaration of Helsinki.

### Data collection and RoB assessment

For each included RCT, we extracted the RoB ratings reported by the original systematic review authors (OSRAs). Separately, three human reviewers (S.G., A.S., and Y.C.), masked to the OSRA’s assessments, independently conducted their own RoB assessments. To ensure a comprehensive evaluation, particularly for selective reporting (Domain 5), our human panel retrieved trial registry entries (e.g., ClinicalTrials.gov) whenever available. Similarly, ChatGPT-o3 assessments were conducted with web-browsing capabilities enabled, allowing the model to theoretically search for and cross-reference external registry data if referenced within the manuscript. Discrepancies between human reviewers were resolved by consensus discussion, or by a fourth author (M.B.), if necessary. Finally, ChatGPT-o3 was used to independently generate RoB assessments for each RCT while being masked to the ratings from our panel and the OSRAs. The ChatGPT-o3 assessments were generated using a structured RoB prompt that was developed by integrating the Cochrane RoB 2.0 guidelines with a detailed template from Lai et al. (2024) (SMethods 2 in S1 File) [9,21]. We accessed the model via the standard web interface (chatgpt.com) between April 20, 2025, and May 20, 2025, utilizing the “ChatGPT-o3” model version. As the web interface does not permit manipulation of generation parameters (e.g., temperature, top_p), default settings were applied. To ensure reproducibility and prevent context accumulation (data leakage between trials), each assessment was performed in a new, independent chat session (single-shot approach). For each trial, the complete PDF of the article was uploaded along with the prompt, allowing the model to parse the full text and tables directly. This prompt explicitly instructs the model to base all judgments strictly on reported information without extrapolation or speculation, following the signaling questions and branching logic found in the RoB 2.0 cribsheet [22].

### Statistical analysis

RoB ratings were compared across three sources: the OSRAs, our manual masked assessment, and the assessments generated by ChatGPT-o3. All ratings were made using the Cochrane RoB 2.0 tool across five individual domains: (1) bias arising from the randomization process, (2) bias due to deviations from intended interventions, (3) bias due to missing outcome data, (4) bias in measurement of the outcome, and (5) bias in selection of the reported result; as well as an overall judgment. Each domain was rated on an ordinal scale with three categories: Low risk, Some concerns, or High risk.

Pairwise interrater agreement was assessed using linearly weighted Cohen κ statistics. Cohen’s κ values were interpreted using standard thresholds: < 0.00 (poor), 0.00–0.20 (slight), 0.21–0.40 (fair), 0.41–0.60 (moderate), 0.61–0.80 (substantial), and 0.81–1.00 (almost perfect) agreement [23]. Agreement was calculated within our panel and between our panel, OSRAs, and ChatGPT-o3 for each domain as well as the overall judgement. 95% confidence intervals (CIs) were estimated using percentile bootstrap methods with 1000 resamples. To assess global agreement among all three raters, Gwet’s AC_2_ was computed. This statistic was chosen over Fleiss’ κ due to its greater stability under conditions of uneven category distribution (the prevalence paradox). In RoB assessments, where a majority of studies may fall into a single category (e.g., “Low Risk”), Kappa often drastically underestimates agreement; Gwet’s AC2 corrects for this by adjusting for chance agreement based on the marginal probabilities of the ratings [24,25]. We also calculated exact percent agreement between each rater pair across domains to provide an intuitive summary of rating concordance.

To examine the direction and magnitude of disagreement, ordinal scores were assigned to ratings (0 = Low risk, 1 = Some concerns, 2 = High risk). Mean signed differences were calculated for each domain and rater pair. Symmetry of disagreement patterns was tested using Bowker’s test for paired ordinal data. To further characterize disagreement severity, we also quantified “extreme disagreements” between raters, defined as instances where one rater assigned a “High risk” and another a “Low risk” rating for the same study within the same domain.

To evaluate diagnostic performance, ratings were dichotomized into High Risk versus Not High Risk. Sensitivity, specificity, and balanced accuracy of ChatGPT-o3 were calculated using our masked human assessment as the reference standard. We selected our panel as the reference because their ratings were generated specifically for this study under blinded conditions, effectively mitigating the potential optimism bias or conflicts of interest that may influence original systematic review authors. However, we acknowledge that RoB assessments possess inherent subjectivity; therefore, this reference represents a rigorous expert consensus rather than an indisputable objective ground truth. A secondary analysis dichotomized ratings as Low Risk versus Not Low Risk. Pairwise McNemar’s tests were also conducted to assess differences in binary classification rates between raters.

All analyses and data visualizations were conducted using R version 4.4.1 (R Foundation for Statistical Computing, Vienna, Austria). To control for family-wise error rates, all reported p-values were Bonferroni-adjusted for the 18 distinct hypotheses tested (3 rater pairs × 6 domains), with a two-sided significance threshold of 0.05.

## Results

A total of 50 RCTs underwent independent RoB assessment using the Cochrane RoB 2.0 tool across five individual domains and an overall RoB judgment. ChatGPT-o3, our masked human panel, and OSRAs independently provided ratings, yielding 300 complete assessments for analysis (Table 1). All full LLM rating responses can be found in SMethods 3 in S1 File.

**Table 1:** Risk of Bias 2.0 ratings across five domains and overall judgment for 50 randomized clinical trials.

	Domain 1*			Domain 2*			Domain 3*			Domain 4*			Domain 5*			Overall		
RCT Authors	OSRA^†^	Our Panel	LLM^‡^	OSRA	Our Panel	LLM	OSRA	Our Panel	LLM	OSRA	Our Panel	LLM	OSRA	Our Panel	LLM	OSRA	Our Panel	LLM
Malmstrom (1999)	Low	Low	Low	Low	Low	Some	Low	Low	Low	Low	Low	Low	Low	Low	Low	Low	Low	Some
Mortensen (1998)	Low	Low	High	Low	Low	Low	Some	High	High	Low	Low	Low	Low	Low	Some	Some	High	High
Davis (2012)	Low	Low	Low	High	Low	Low	Some	High	Low	Low	Low	Low	Low	Low	Low	Low	High	Low
García-Morales (2020)	Low	Low	Some	Low	High	Some	Low	Low	Some	High	High	High	Some	Some	Low	High	High	High
Arntz (2003)	High	High	High	High	High	Low	Some	High	High	Low	High	High	Some	Some	High	High	High	High
Marsh (2015)	Low	Low	Low	Low	Low	Low	Low	Low	Some	Low	Some	Low	Low	Some	Some	Low	Some	Some
Stryjer (2010)	High	High	Some	Low	Low	Low	Low	Low	Low	Low	Some	Low	Low	Some	Some	High	High	Some
Grob (2011)	Low	Low	Low	Some	High	Low	Low	Low	High	Low	High	High	Low	High	High	Some	High	High
Colhoun (2004)	Low	Low	Low	Some	Low	Low	Low	Low	Low	Low	Low	Low	Low	Low	Low	Some	Low	Low
Dehghan (2022)	Low	Low	Low	Low	Low	Some	Low	Low	Low	Low	Low	Some	Low	Low	Low	Low	Low	Some
Vernon (2012)	Low	Low	Low	Some	Low	Some	Low	Low	Low	Low	Low	Low	Low	Some	Some	Some	Some	Some
Duzgun (2019)	Some	Some	Some	Low	Low	Low	Low	Low	Low	Low	Low	Low	Some	Some	Some	Some	Some	Some
de Alencar (2021)	Low	Low	Low	Low	Low	Low	Low	Low	Low	Low	Low	Low	Low	Low	Low	Low	Low	Low
Ghavane (2012)	Low	Low	Low	Low	Low	Low	Low	Low	Low	Low	Low	Low	Low	Low	Some	Low	Low	Some
James (2016)	Low	Low	Low	Low	Low	Low	Low	Low	Low	Low	Low	Low	Low	Low	Low	Low	Low	Low
Chawanpaiboon (2021)	High	High	High	High	High	Some	Low	High	High	Low	High	Some	High	Low	Some	High	High	High
Diakomi (2014)	Low	Some	High	Low	Low	Some	Low	Low	Low	Low	Low	Low	Low	Low	Low	Low	Some	High
Dobkin (2020)	Low	Low	Low	Low	Low	Low	Low	High	Low	Some	Low	Low	Low	Low	Low	Some	High	Low
Babalola (2021)	Low	Some	Some	Low	Some	Some	Low	Low	Low	Low	Low	Low	Low	Some	Low	Low	High	Some
Pavlou (2023)	Low	Some	High	Low	Low	Low	Low	Low	Low	Some	Low	Low	Some	Low	Low	Some	Some	High
Rahman (2021)	Some	Some	Low	Low	Low	Some	Low	Low	Low	Low	Low	Low	Some	Some	Low	Some	Some	Some
Vijayaraghavan (2022)	Low	Low	Low	Low	Low	Low	Low	Low	Low	Low	Low	Low	Low	Low	Low	Low	Low	Low
Leo (2010)	Low	Low	Low	Low	Low	Low	Low	Low	Low	Low	Low	Low	Some	Low	Some	Some	Low	Some
Collins (2017)	Low	Low	Low	Low	Low	Low	Low	Low	Low	Low	Low	Low	Low	Low	Low	Low	Low	Low
Olson (2006)	Low	Some	High	Some	Low	Low	Low	Low	Low	Low	Low	Low	Low	Some	Low	Some	Some	High
de Jonghe (2014)	Low	Low	Low	Low	Low	Some	Low	Some	High	Low	Low	Low	Low	Low	Some	Low	Some	High
Palmer (2013)	Low	Low	Some	Low	Low	Low	Low	Some	Low	Low	Low	Low	Low	Low	Some	Low	Some	Some
Kim (2020)	Low	Low	Some	Low	Low	High	Low	Low	High	Low	Low	High	Low	Low	High	Low	Low	High
Gazal (2007)	Low	Low	Low	Low	Low	Some	Low	Low	Low	Low	Low	High	Low	Some	Some	Low	Some	High
Reischig (2018)	Low	Low	Some	Low	Low	Some	Low	Some	High	Low	Low	Low	Low	Low	Low	Low	Some	High
Ventura-López (2022)	Low	Some	Low	High	Low	Low	Low	Low	Low	Low	Low	Low	Low	Some	Some	High	Some	Some
Jamshed (2022)	Some	Some	High	Low	Low	Low	Low	Low	Low	Low	Low	Low	Low	Low	Low	Some	Some	High
Holroyd (2010)	Low	Low	Low	Low	Some	Low	Low	Some	Low	Low	Some	Low	High	Some	Some	High	High	Some
Parry (2009)	Low	Low	Some	Low	Low	High	Low	Low	High	Low	Low	Low	Some	Some	Some	Some	Some	High
Louw (2019)	Some	Some	Some	Low	Low	Low	Low	Low	Low	Low	Low	Low	Low	Low	Low	Some	Some	Some
Dougan (2021)	Low	Low	Some	Low	Low	Some	Low	Low	Low	Low	Low	Low	Low	Low	Low	Low	Low	Some
Mummolo (2013)	Low	Some	Some	Low	Low	Low	Low	Low	Low	Low	Low	Low	Low	Some	Some	Low	Some	Some
Brunello (2008)	Low	Low	Low	Low	Low	Low	Low	Low	Low	Low	Low	Some	Low	Some	Low	Low	Some	Some
Shapiro (1998)	Low	Low	Some	Low	Low	Some	Low	Low	High	Low	Low	High	Some	Some	Some	Some	Some	High
Pfeiffer (2020)	Low	Low	Low	Low	Some	Low	Low	Low	Low	Low	Some	Low	Low	Some	Some	Low	High	Some
Girard (2008)	Low	Low	Low	Low	Low	Low	Low	Low	Low	Low	Some	Low	Low	Low	Low	Low	Some	Low
Kocherov (2013)	Low	Some	High	Some	Low	Low	Low	Low	Low	Low	Some	Low	Low	Low	Some	Low	Some	High
Aliabadi (2023)	Low	Low	Low	Low	Low	Low	Low	Low	Low	Low	Low	Low	Low	Low	Low	Low	Low	Low
Coron (2016)	Low	Low	High	Low	Low	High	Low	Low	Low	Low	Low	Low	Low	Low	Some	Low	Low	High
Wallström (2019)	Low	Low	Low	Low	Some	Some	Low	Low	Low	Low	Some	Low	Low	Low	Low	Low	Some	Some
Elbarbary (2020)	Low	Low	Low	Low	Low	Low	Low	Low	Low	Low	Low	Some	Low	Low	Some	Low	Low	Some
Held (2020)	Low	Some	Some	Low	Low	Low	Low	Low	Low	Low	Some	Low	Some	Low	Some	Some	Some	Some
Rerksupphaphol (2020)	Low	Low	Low	Low	Low	Low	Low	Low	Low	Low	Low	Low	Some	Low	Low	Some	Low	Low
Vallentin (2025)	Low	Low	Low	Low	Some	Low	Low	Low	Low	Low	Some	Low	Low	Low	Low	Low	Some	Low
Kyriazopoulou (2021)	Low	Some	Low	Low	Some	Low	Low	Low	Low	Low	Low	Low	Low	Low	Low	Low	Some	Low

*Domain 1 – Domain 5 refer to the following Risk of Bias (RoB) 2.0 domains, respectively: (1) bias arising from the randomization process, (2) bias due to deviations from intended interventions, (3) bias due to missing outcome data, (4) bias in measurement of the outcome, and (5) bias in selection of the reported result.

†OSRA refers to the RoB ratings by the original authors of the 50 systematic reviews.

‡LLM refers to the RoB ratings by ChatGPT-o3.

### Distribution of high-risk judgments and extreme disagreement patterns

**Fig 1:**
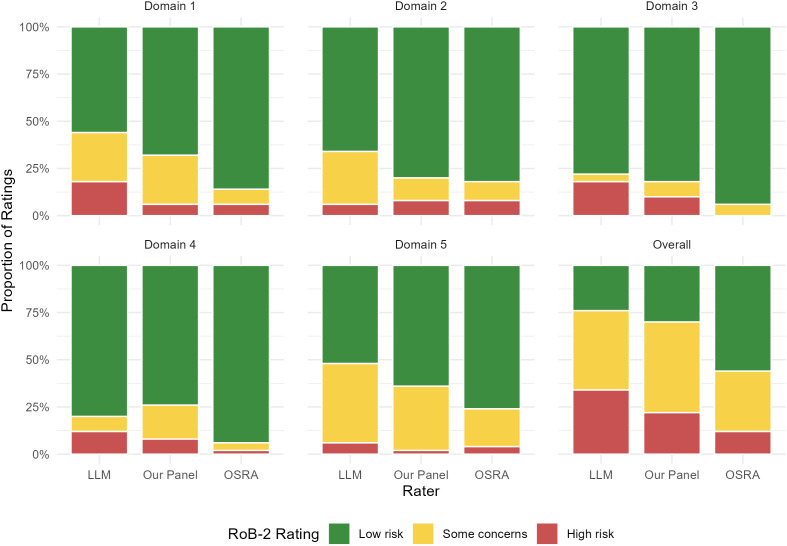
Distribution of Risk of Bias 2.0 Ratings by Rater and Domain. Stacked bar plots showing the distribution of Risk of Bias 2.0 ratings assigned by ChatGPT-o3 (LLM), our panel, and the original systematic review authors (OSRAs) across each risk of bias domain (Domain 1 to Domain 5) and overall. Ratings are categorized as low risk (green), some concerns (yellow), or high risk (red).

The distribution of RoB ratings varied notably across raters (Fig 1). ChatGPT-o3 classified 34% of trials (17 of 50) as high risk, compared with 22% (11 of 50) by our panel and 12% (6 of 50) by the OSRAs. ChatGPT-o3 consistently assigned high-risk ratings more frequently than our panel across most domains, with the largest differences observed in domain 1 (18% vs 6%), domain 3 (18% vs 10%), domain 4 (12% vs 8%), and domain 5 (6% vs 2%). However, none of these differences were significant (all p > 0.05). We also assessed extreme disagreements, defined as one rater assigning a “High risk” and another a “Low risk” rating for the same study. Between ChatGPT-o3 and our panel, these occurred in 4 cases (8%), split evenly in both directions. In contrast, extreme discrepancies between ChatGPT-o3 and OSRAs were more unidirectional, with ChatGPT-o3 rating 7 trials (14%) as “High risk” that OSRAs had judged “Low risk”; the reverse did not occur. Between OSRAs and our panel, extreme discrepancies were likewise unidirectional, with our panel rating 3 trials (6%) as “High risk” that OSRAs had judged “Low risk”; the reverse did not occur.

### Interrater agreement

Pairwise agreement, assessed using linear-weighted Cohen κ, demonstrated limited consistency overall (Table 2). We conducted 18 pairwise comparisons by evaluating agreement between each pair of raters (ChatGPT-o3, our masked panel, and the OSRAs) across the five individual RoB domains and the overall rating. Of these 18 comparisons, 10 (56%) reflected fair or moderate agreement, while 6 (33%) showed slight and 1 (6%) poor agreement, highlighting substantial variability in concordance. Median κ values across all domains were highest between ChatGPT-o3 and our panel (0.33), followed by the OSRAs and our panel (0.28), and lowest between ChatGPT-o3 and OSRAs (0.14). For the overall RoB judgment, Fleiss’s κ across all three raters was 0.16. The strongest individual agreement occurred between the OSRAs and our panel in domain 1 (κ = 0.63; 95% CI, 0.31–0.83), while the weakest occurred between ChatGPT-o3 and the OSRAs in domain 2 (κ = –0.10; 95% CI, –0.25 to 0.08), suggesting near-random agreement in that domain.

**Table 2:** Pairwise interrater agreement (linear-weighted Cohen κ) by risk of Bias 2.0 domain.

Domain	LLM* vs Our Panel	OSRA^†^ vs Our Panel	LLM vs OSRA
Domain 1	0.43 (0.21–0.61)^‡^	0.63 (0.31–0.83)	0.26 (0.05–0.48)
Domain 2	0.03 (−0.18–0.24)	0.25 (−0.13–0.56)	−0.10 (−0.25–0.08)
Domain 3	0.36 (0.04–0.65)	0.31 (0.00–0.50)	0.12 (−0.04–0.36)
Domain 4	0.29 (−0.10–0.56)	0.12 (−0.12–0.43)	0.14 (−0.09–0.47)
Domain 5	0.37 (0.09–0.60)	0.22 (−0.05–0.47)	0.15 (−0.06–0.34)
Overall	0.23 (0.00–0.43)	0.36 (0.15–0.55)	0.14 (−0.04–0.30)

*LLM refers to ChatGPT-o3.

†OSRA refers to the original systematic review authors.

‡Values are κ (95% CI) estimated via percentile bootstrap (1000 resamples).

Exact percent agreement also varied widely, ranging from 36% for overall RoB ratings between ChatGPT-o3 and OSRAs to 82% for domains 1 and 3 between OSRAs and our panel. For the overall RoB judgment specifically, agreement was 54% between OSRAs and our panel, 46% between ChatGPT-o3 and our panel, and 36% between ChatGPT-o3 and OSRAs. A domain-level heatmap of exact agreement is provided in SFigure 1 in S1 File. To better understand internal consistency within our reference group, we conducted an additional analysis of interrater reliability among members of our masked panel. Agreement varied across domains, with percent agreement ranging from 60% to 90%, and linear-weighted κ values ranging from 0.21 (fair agreement) to 0.74 (substantial agreement). For the overall RoB judgment, percent agreement was 70%, with a linear-weighted κ of 0.56, reflecting moderate internal agreement. Full results are provided in STable 1 in S1 File.

Global agreement across all three raters, as assessed using Gwet’s AC_2_, was higher for individual domains (range, 0.64–0.79) but lower for the overall rating (AC_2_ = 0.30) (STable 2 in S1 File). Pairwise AC_2_ values followed a similar pattern: overall agreement was 0.33 (ChatGPT-o3 vs our panel), 0.15 (ChatGPT-o3 vs OSRA), and 0.46 (OSRA vs our panel), with domain-level pairwise AC_2_ values ranging from 0.56 to 0.87 (STable 3 in S1 File). In the within-panel analysis, AC_2_ values were higher across domains (range, 0.66–0.93), and agreement remained strong overall at 0.71. (STable 4 in S1 File).

### Directionality of rating differences

Analysis of disagreement patterns revealed systematic differences in rating tendencies. To quantify directional differences, categorical RoB judgments were converted to ordinal scores (0 for low risk, 1 for some concerns, and 2 for high risk). ChatGPT-o3 consistently applied more severe ratings than our panel, with a positive mean difference of 0.18 for overall RoB assessments (p > 0.05, STable 5 in S1 File). In contrast, OSRAs assigned lower-risk ratings relative to our panel (mean difference, –0.36, p < 0.01), with an even larger discrepancy observed between OSRAs and ChatGPT-o3 (mean difference, –0.54, p < 0.001). Bowker symmetry tests identified significant asymmetry in domain 1 ratings between OSRAs and our panel (p < 0.05; STable 6 in S1 File), whereas all other comparisons across domains and rater pairs were not statistically significant (p > 0.05).

### Diagnostic accuracy relative to panel ratings

Using our masked panel ratings as the reference standard, ChatGPT-o3 demonstrated moderate sensitivity (0.46) and specificity (0.69) for identifying high-risk studies, resulting in a balanced accuracy of 0.57 (Table 3). The OSRAs had the same sensitivity (0.46) but substantially higher specificity (0.97), yielding a higher balanced accuracy of 0.71. When identifying low-risk studies (Table 4), ChatGPT-o3 achieved a balanced accuracy of 0.66 (sensitivity, 0.47; specificity, 0.86), while OSRAs demonstrated similar overall performance (balanced accuracy, 0.67), with higher sensitivity (0.80) but lower specificity (0.54).

**Table 3:** Diagnostic performance for detecting high‑risk studies.

Domain	Rater Pair*	Sensitivity	Specificity	Balanced Accuracy
Domain 1	LLM^†^ – Our Panel	0.67	0.85	0.76
	OSRA^‡^ – Our Panel	1.00	1.00	1.00
	LLM – OSRA	0.67	0.85	0.76
Domain 2	LLM – Our Panel	0.00	0.93	0.47
	OSRA – Our Panel	0.50	0.96	0.73
	LLM – OSRA	0.00	0.93	0.47
Domain 3	LLM – Our Panel	0.60	0.87	0.73
	OSRA – Our Panel	0.00	1.00	0.50
	LLM – OSRA	—	0.82	0.82
Domain 4	LLM – Our Panel	0.75	0.93	0.84
	OSRA – Our Panel	0.25	1.00	0.63
	LLM – OSRA	1.00	0.90	0.95
Domain 5	LLM – Our Panel	1.00	0.96	0.98
	OSRA – Our Panel	0.00	0.96	0.48
	LLM – OSRA	0.00	0.94	0.47
Overall	LLM – Our Panel	0.46	0.69	0.57
	OSRA – Our Panel	0.46	0.97	0.71
	LLM – OSRA	0.50	0.68	0.59

*Test versus reference: sensitivity and specificity are computed with respect to the second‐named rater in each pair (i.e., “Our Panel” for LLM–Our Panel & OSRA–Our Panel; “OSRA” for LLM–OSRA).

†LLM refers to ChatGPT-o3.

‡OSRA refers to the original systematic review authors.

Note: Balanced accuracy is the mean of sensitivity and specificity. A dash indicates sensitivity was not estimable because the reference had no positive cases.

**Table 4:** Diagnostic performance for detecting low‑risk studies.

Domain	Rater Pair*	Sensitivity	Specificity	Balanced Accuracy
Domain 1	LLM^†^ – Our Panel	0.74	0.81	0.77
	OSRA^‡^ – Our Panel	1.00	0.44	0.72
	LLM – OSRA	0.63	0.86	0.74
Domain 2	LLM – Our Panel	0.68	0.40	0.54
	OSRA – Our Panel	0.85	0.30	0.58
	LLM – OSRA	0.63	0.22	0.43
Domain 3	LLM – Our Panel	0.85	0.56	0.70
	OSRA – Our Panel	1.00	0.33	0.67
	LLM – OSRA	0.81	0.67	0.74
Domain 4	LLM – Our Panel	0.84	0.31	0.57
	OSRA – Our Panel	0.95	0.08	0.51
	LLM – OSRA	0.81	0.33	0.57
Domain 5	LLM – Our Panel	0.66	0.72	0.69
	OSRA – Our Panel	0.84	0.39	0.61
	LLM – OSRA	0.58	0.67	0.62
Overall	LLM – Our Panel	0.47	0.86	0.66
	OSRA – Our Panel	0.80	0.54	0.67
	LLM – OSRA	0.32	0.86	0.59

*Test versus reference: sensitivity and specificity are computed with respect to the second‐named rater in each pair (i.e., “Our Panel” for LLM–Our Panel & OSRA–Our Panel; “OSRA” for LLM–OSRA).

†LLM refers to ChatGPT-o3.

‡OSRA refers to the original systematic review authors.

Note: Balanced accuracy is the mean of sensitivity and specificity. A dash indicates sensitivity was not estimable because the reference had no positive cases.

## Discussion

This study evaluated the reliability and diagnostic performance of ChatGPT-o3 in assessing RoB in RCTs using the RoB 2.0 tool. While the model demonstrated promising consistency and potential as a screening tool, systematic deviations from human reviewers limit its readiness for standalone use. Our findings diverge from earlier studies, such as Lai et al. (2024), who reported high accuracy (>80%) using a modified version of the simpler Cochrane RoB 1.0 tool [9]. In contrast, evaluations utilizing the complex, domain-based RoB 2.0 tool have yielded significantly lower performance, highlighting a reasoning gap in current LLMs. For instance, Šuster et al. (2024) reported F1 scores as low as 0.10–0.20 for zero-shot RoB 2.0 predictions, concluding that the task complexity currently exceeds off-the-shelf model capabilities [11]. Similarly, Huang et al. (2025) found that while LLMs could identify signaling questions with reasonable accuracy, their ability to derive correct domain-level judgments dropped to 57.5%, which is a figure strikingly similar to our balanced accuracy of 0.57 [12]. This difficulty in synthesizing methodological nuances is further corroborated by Hasan et al., who reported only 61% agreement for the similarly complex ROBINS-I tool [13]. Collectively, these data confirm that while LLMs excel at information extraction, they struggle with the algorithmic logic required for advanced bias assessment tools. ChatGPT-o3 consistently assigned higher RoB ratings than both our masked panel and the OSRAs, likely reflecting a more conservative or literal interpretation of signaling criteria. This tendency persisted even though the model had web-browsing capabilities enabled. This strongly suggests that our prompting strategy may have been the primary driver of these conservative ratings: by explicitly instructing the model to assess strictly based on reported information without extrapolation, we likely inhibited it from utilizing its browsing tool to verify external protocols. Consequently, the model penalized incomplete reporting, such as assigning “Some concerns” or “High risk” where human reviewers (who accessed registries) confirmed low risk. This stricter approach may help identify subtle or underreported sources of bias, reducing false negatives, but it risks overclassifying studies as high risk. Consequently, this could lower the certainty of evidence in Grading of Recommendations, Assessment, Development and Evaluations (GRADE) assessments and negatively impact clinical recommendations. In contrast, OSRAs tended toward more lenient ratings, possibly reflecting implicit incentives to classify studies as lower risk, thereby increasing overall certainty and strength of conclusions. This underscores the value of independent reviews to counterbalance bias.

The divergence between ChatGPT-o3 and human raters underscores the potential value of LLMs as adjunct reviewers. By identifying inconsistencies and offsetting overly permissive human ratings, LLMs can support authors or journal editors. Specifically, it may be possible to have a “human-in-the-loop” workflow where the LLM functions as an adjunctive, conservative screener to flag potential risks. While the model’s moderate sensitivity (0.46) indicates it should not be relied upon to catch every high-risk trial independently, its tendency toward strict criteria results in a high volume of flagged studies. In this capacity, reviewers must accept a higher rate of false positives that human experts can then adjudicate. This approach leverages the model’s rigid adherence to signaling questions to highlight potential methodological flaws, thereby allowing human reviewers to focus their cognitive effort on verifying flagged domains rather than performing entirely de novo extraction. A hybrid model combining automated screening with expert oversight could offer a transparent and scalable approach, particularly in high-volume evidence synthesis contexts.

Overall interrater reliability, as measured by Gwet’s AC_2_, was modest, reflecting both the inherent subjectivity of RoB assessments and limitations in LLMs replicating nuanced judgment. Notably, agreement within our own panel varied substantially across domains, highlighting that discrepancies between ChatGPT-o3 and human raters may stem more from structural features of the RoB tool rather than individual reviewer performance. Agreement was notably weakest for Domain 2 (deviations from intended interventions), which yielded near-random concordance. This finding is consistent with landmark validation studies of RoB 2.0; for instance, Minozzi et al. (2020) reported an interrater reliability of only 0.04 for this domain among experienced reviewers [26]. This specific complexity arises because RoB 2.0 requires reviewers to distinguish between the effect of assignment to intervention and the effect of adhering to intervention, a process that necessitates nuanced interpretation of protocol deviations and their potential impact on the trial results. Beyond this individual domain, our overall results align with broader research documenting that RoB 2.0 consistently exhibits low to moderate interrater reliability (κ often < 0.50) even among trained methodologists [4,26,27]. In the Minozzi et al. study, human agreement for the overall RoB 2.0 judgment was as low as 0.16, which is categorized as slight agreement [23]. Such inherent subjectivity complicates the designation of any single human rating as an objective gold standard. Consequently, we utilized our blinded panel as the reference not because it represents a definitive truth, but because it offers a consensus-based benchmark independent of the potential incentives or outdated methods that may influence OSRAs. Indeed, low agreement between SR authors and our panel was somewhat comparable to internal panel variability, underscoring the need for improved guidance, training, or consensus processes to enhance consistency across reviewers.

ChatGPT-o3 exhibited moderate sensitivity (0.46) and specificity (0.69) in identifying high-risk trials, indicating some capacity to flag problematic studies, though it may miss many genuinely high-risk cases. For low-risk trials, specificity was higher (0.86), with similar sensitivity (0.47), reflecting a cautious approach that avoids false positives but may overlook genuinely low-risk studies. These results suggest a practical role for LLMs in triage workflows. For example, they could help deprioritize clearly low-risk studies for manual review, thereby improving overall efficiency. However, prospective validation is needed to confirm accuracy and maintain integrity.

Several limitations warrant consideration. First, our search strategy employed specific quoted terms; while necessary to identify studies utilizing the relatively recent RoB 2.0 framework, this strict approach may have excluded relevant meta-analyses that utilized alternative terminology or abbreviations (e.g., “RoB2” or “Risk of Bias tool 2”). Consequently, our sample may represent a subset of trials with more standardized reporting, potentially narrowing the diversity of reporting styles assessed. Second, we acknowledge the potential for data leakage. Since the meta-analyses in our sample were published prior to the model’s training cutoff, the LLM may have been exposed to the OSRA ratings during training. However, the low agreement observed between ChatGPT-o3 and the OSRAs (κ = 0.14) suggests the model was not simply regurgitating memorized training data but rather generating de novo judgments. Third, while newer iterations such as ChatGPT-5.2 have since been released, ChatGPT-o3 represented the state-of-the-art at the time of study design. Additionally, the evaluation involved only one LLM and one prompting strategy; other models or prompts, particularly fine-tuned on methodological content, may yield different outcomes. Furthermore, larger datasets would enhance precision and enable subgroup analyses based on trial characteristics or reporting quality. Time saved, a critical practical consideration, was not assessed. Consequently, we cannot calculate the net benefit of the LLM; without efficiency data, it is unclear if the time required to verify the model’s frequent “High Risk” flags outweighs the time taken to conduct a manual review from scratch. Additionally, although web browsing was enabled, we did not quantify how frequently the model successfully retrieved external protocols compared to human reviewers, which limits the transparency of the model’s information-gathering process. It appears the model may have prioritized our strict textual constraints over its ability to browse external data; however, without access to the model’s internal browsing logs, we cannot definitively determine when external searches were attempted or failed. Future research should perform sensitivity analyses using varied prompting strategies, such as specifically testing “optimistic” prompts that explicitly command the model to search for and ingest registry data, to determine if this yields better alignment with human raters. Furthermore, because this study focused solely on English-language RCTs, generalizability to non-English contexts remains uncertain. Finally, our study utilized a generalist approach, assessing trials across 50 different medical subspecialties. Unlike human experts who often possess deep domain-specific knowledge, which allows them to detect subtle field-specific biases (e.g., blinding difficulties in surgical trials versus pharmacological trials), the LLM applied a generalized logic. This lack of domain-specific context may have contributed to the lower agreement observed, suggesting that future implementations might require fine-tuning on specialty-specific literature.

Future research should investigate the performance of diverse LLMs across various tasks and contexts, including comparisons between general-purpose and domain-specific models. Prospective evaluations within real-world workflows will clarify practical utility concerning efficiency, reproducibility, and reviewer consistency. Additionally, exploring language-specific performance, international generalizability, and assessing LLM utility with other RoB tools beyond RCTs is essential. Alternative output formats such as confidence scores or probabilistic judgments may also enhance nuance and more accurately reflect uncertainty. Collectively, these efforts will support the responsible and scalable integration of LLMs into evidence synthesis.

Ultimately, ChatGPT-o3 showed potential as an adjunctive tool for RoB assessment in RCTs, consistently applying stricter criteria than both our masked panel and SR authors. While not yet ready for standalone use, the model may be useful in screening workflows or as a secondary reviewer. Continued development and validation will be essential to support the responsible integration of LLMs into SR practices.

## Supporting information

S1 File Supporting information. This file contains all supplementary methods, figures and tables.
(DOCX)

S2. File TRIPOD-LLM Checklist. Completed TRIPOD-LLM checklist for this study.
(DOCX)
