# Supplementary material for: Evaluating large language model performance in Risk of Bias assessments: A cross-sectional validation study
Source: PLoS One. 2026 Jul 9;21(7):e0353155. doi: 10.1371/journal.pone.0353155 (PMC13349098; doi:10.1371/journal.pone.0353155)
Supplement: S1 File — This file contains all supplementary methods, figures and tables. (DOCX) [file pone.0353155.s001.docx]

SMethods 1. Citations for RCTs and Systematic Reviews

SFigure 1. Exact Percent Agreement Between Rater Pairs by Risk of Bias 2.0 Domain

STable 1. Inter-Rater Agreement Among Our Masked Raters Across Risk of Bias 2.0 Domains

STable 2. Global Inter-Rater Agreement (Gwet’s AC2) by Risk of Bias 2.0 Domain

STable 3. Pairwise Inter-Rater Agreement (Gwet’s AC2 95% CI) by Risk of Bias 2.0 Domain

STable 4. Within Panel Inter-Rater Agreement (Gwet’s AC2) by Risk of Bias 2.0 Domain

STable 5. Mean Signed Differences in RoB‑2 Ratings

STable 6. Bowker Test for Symmetry of Paired Ordinal Ratings

SMethods 2: Risk of Bias LLM Prompt

SMethods 3: ChatGPT-o3 Responses

| **SMethods 1. Citations for RCTs and Systematic Reviews** | |
| --- | --- |
| **RCT Authors** | **Systematic Review Authors** |
| Malmstrom (1999)(1) | Mayoral (2023)(2) |
| Mortensen (1998)(3) | Ardebili (2023)(4) |
| Davis (2012)(5) | García-Hermoso (2023)(6) |
| García-Morales (2020)(7) | Schönenberger (2021)(8) |
| Arntz (2003)(9) | Papola (2024)(10) |
| Marsh (2015)(11) | Balachandran (2022)(12) |
| Stryjer (2010)(13) | Gerolymos (2024)(14) |
| Grob (2011)(15) | Metaxa (2024)(16) |
| Colhoun (2004)(17) | Byrne (2022)(18) |
| Dehghan (2022)(19) | Hisamune (2024)(20) |
| Vernon (2012)(21) | Pankrath (2024)(22) |
| Duzgun (2019)(23) | Olguín-Huerta (2023)(24) |
| de Alencar (2021)(25) | Siemieniuk (2020)(26) |
| Ghavane (2012)(27) | Narciso (2022)(28) |
| James (2016)(29) | Vale (2023)(30) |
| Chawanpaiboon (2021)(31) | Levene (2024)(32) |
| Diakomi (2014)(33) | Guay (2020)(34) |
| Dobkin (2020)(35) | Pigott (2022)(36) |
| Babalola (2021)(37) | Marcolino (2022)(38) |
| Pavlou (2023)(39) | Liu (2024)(40) |
| Rahman (2021)(41) | Argueta-Figueroa (2022)(42) |
| Vijayaraghavan (2022)(43) | Zhou (2023)(44) |
| Leo (2010)(45) | Cartledge (2022)(46) |
| Collins (2017)(47) | Marc (2023)(48) |
| Olson (2006)(49) | Cortes (2023)(50) |
| de Jonghe (2014)(51) | Barnes (2023)(52) |
| Palmer (2013)(53) | Bärebring (2022)(54) |
| Kim (2020)(55) | Bistolfi (2022)(56) |
| Gazal (2007)(57) | Miroshnychenko (2023)(58) |
| Reischig (2018)(59) | Ruenroengbun (2022)(60) |
| Ventura-López (2022)(61) | Erickson (2023)(62) |
| Jamshed (2022)(63) | Xie (2024)(64) |
| Holroyd (2010)(65) | Versijpt (2024)(66) |
| Parry (2009)(67) | Bourke (2024)(68) |
| Louw (2019)(69) | Sit (2021)(70) |
| Dougan (2021)(71) | Siemieniuk (2021)(72) |
| Mummolo (2013)(73) | Coronel-Zubiate (2024)(74) |
| Brunello (2008)(75) | Wade (2023)(76) |
| Shapiro (1998)(77) | da Silva (2023)(78) |
| Pfeiffer (2020)(79) | Pliannuom (2024)(80) |
| Girard (2008)(81) | Hernandez (2024)(82) |
| Kocherov (2013)(83) | Nurtamin (2022)(84) |
| Aliabadi (2023)(85) | Asante (2024)(86) |
| Coron (2016)(87) | Sasso (2022)(88) |
| Wallström (2019)(89) | Pergialiotis (2023)(90) |
| Elbarbary (2020)(91) | Ferraz (2024)(92) |
| Held (2020)(93) | Castilla-López (2022)(94) |
| Rerksupphaphol (2020)(95) | Ali (2024)(96) |
| Vallentin (2025)(97) | Couper (2025)(98) |
| Kyriazopoulou (2021)(99) | Davidson (2022)(100) |

**REFERENCES**

1. Malmstrom K, Rodriguez-Gomez G, Guerra J, Villaran C, Piñeiro A, Wei LX, et al. Oral montelukast, inhaled beclomethasone, and placebo for chronic asthma. A randomized, controlled trial. Montelukast/Beclomethasone Study Group. Ann Intern Med. 1999 Mar 16;130(6):487–95.

2. Mayoral K, Lizano-Barrantes C, Zamora V, Pont A, Miret C, Barrufet C, et al. Montelukast in paediatric asthma and allergic rhinitis: a systematic review and meta-analysis. Eur Respir Rev Off J Eur Respir Soc. 2023 Dec 31;32(170):230124.

3. Mortensen L, Charles P, Bekker PJ, Digennaro J, Johnston CC. Risedronate increases bone mass in an early postmenopausal population: two years of treatment plus one year of follow-up. J Clin Endocrinol Metab. 1998 Feb;83(2):396–402.

4. Ahadzadeh Ardebili A, Fu T, Dunnewold N, Aghajafari F, Billington EO. Bisphosphonates Preserve Bone Mineral Density and Suppress Bone Turnover Markers in Early Menopausal Women: A Systematic Review and Meta-Analysis of Randomized Trials. JBMR Plus. 2023 Jun;7(6):e10748.

5. Davis CL, Pollock NK, Waller JL, Allison JD, Dennis BA, Bassali R, et al. Exercise dose and diabetes risk in overweight and obese children: a randomized controlled trial. JAMA. 2012 Sep 19;308(11):1103–12.

6. García-Hermoso A, López-Gil JF, Izquierdo M, Ramírez-Vélez R, Ezzatvar Y. Exercise and Insulin Resistance Markers in Children and Adolescents With Excess Weight: A Systematic Review and Network Meta-Analysis. JAMA Pediatr. 2023 Dec 1;177(12):1276–84.

7. García-Morales JM, Lozada-Mellado M, Hinojosa-Azaola A, Llorente L, Ogata-Medel M, Pineda-Juárez JA, et al. Effect of a Dynamic Exercise Program in Combination With Mediterranean Diet on Quality of Life in Women With Rheumatoid Arthritis. J Clin Rheumatol Pract Rep Rheum Musculoskelet Dis. 2020 Oct;26(7S Suppl 2):S116–22.

8. Schönenberger KA, Schüpfer AC, Gloy VL, Hasler P, Stanga Z, Kaegi-Braun N, et al. Effect of Anti-Inflammatory Diets on Pain in Rheumatoid Arthritis: A Systematic Review and Meta-Analysis. Nutrients. 2021 Nov 24;13(12):4221.

9. Arntz A. Cognitive therapy versus applied relaxation as treatment of generalized anxiety disorder. Behav Res Ther. 2003 Jun;41(6):633–46.

10. Papola D, Miguel C, Mazzaglia M, Franco P, Tedeschi F, Romero SA, et al. Psychotherapies for Generalized Anxiety Disorder in Adults: A Systematic Review and Network Meta-Analysis of Randomized Clinical Trials. JAMA Psychiatry. 2024 Mar 1;81(3):250–9.

11. Marsh AP, Miller ME, Rejeski WJ, Hutton SL, Kritchevsky SB. Lower extremity muscle function after strength or power training in older adults. J Aging Phys Act. 2009 Oct;17(4):416–43.

12. Balachandran AT, Steele J, Angielczyk D, Belio M, Schoenfeld BJ, Quiles N, et al. Comparison of Power Training vs Traditional Strength Training on Physical Function in Older Adults: A Systematic Review and Meta-analysis. JAMA Netw Open. 2022 May 2;5(5):e2211623.

13. Stryjer R, Rosenzcwaig S, Bar F, Ulman AM, Weizman A, Spivak B. Trazodone for the treatment of neuroleptic-induced acute akathisia: a placebo-controlled, double-blind, crossover study. Clin Neuropharmacol. 2010;33(5):219–22.

14. Gerolymos C, Barazer R, Yon DK, Loundou A, Boyer L, Fond G. Drug Efficacy in the Treatment of Antipsychotic-Induced Akathisia: A Systematic Review and Network Meta-Analysis. JAMA Netw Open. 2024 Mar 4;7(3):e241527.

15. Grob CS, Danforth AL, Chopra GS, Hagerty M, McKay CR, Halberstadt AL, et al. Pilot study of psilocybin treatment for anxiety in patients with advanced-stage cancer. Arch Gen Psychiatry. 2011 Jan;68(1):71–8.

16. Metaxa AM, Clarke M. Efficacy of psilocybin for treating symptoms of depression: systematic review and meta-analysis. BMJ. 2024 May 1;385:e078084.

17. Colhoun HM, Betteridge DJ, Durrington PN, Hitman GA, Neil HAW, Livingstone SJ, et al. Primary prevention of cardiovascular disease with atorvastatin in type 2 diabetes in the Collaborative Atorvastatin Diabetes Study (CARDS): multicentre randomised placebo-controlled trial. Lancet Lond Engl. 2004 Aug 21;364(9435):685–96.

18. Byrne P, Demasi M, Jones M, Smith SM, O’Brien KK, DuBroff R. Evaluating the Association Between Low-Density Lipoprotein Cholesterol Reduction and Relative and Absolute Effects of Statin Treatment: A Systematic Review and Meta-analysis. JAMA Intern Med. 2022 May 1;182(5):474–81.

19. Dehghan N, Nauth A, Schemitsch E, Vicente M, Jenkinson R, Kreder H, et al. Operative vs Nonoperative Treatment of Acute Unstable Chest Wall Injuries: A Randomized Clinical Trial. JAMA Surg. 2022 Nov 1;157(11):983–90.

20. Hisamune R, Kobayashi M, Nakasato K, Yamazaki T, Ushio N, Mochizuki K, et al. A meta-analysis and trial sequential analysis of randomised controlled trials comparing nonoperative and operative management of chest trauma with multiple rib fractures. World J Emerg Surg WJES. 2024 Mar 19;19(1):11.

21. Vernon HT, Triano JJ, Ross JK, Tran SK, Soave DM, Dinulos MD. Validation of a novel sham cervical manipulation procedure. Spine J Off J North Am Spine Soc. 2012 Nov;12(11):1021–8.

22. Pankrath N, Nilsson S, Ballenberger N. Adverse Events After Cervical Spinal Manipulation - A Systematic Review and Meta-Analysis of Randomized Clinical Trials. Pain Physician. 2024 May;27(4):185–201.

23. Duzgun I, Turgut E, Eraslan L, Elbasan B, Oskay D, Atay OA. Which method for frozen shoulder mobilization: manual posterior capsule stretching or scapular mobilization? J Musculoskelet Neuronal Interact. 2019 Sep 1;19(3):311–6.

24. Olguín-Huerta C, Araya-Quintanilla F, Moncada-Ramírez V, Estrella-Flores E, Cuyúl-Vásquez I, Gutiérrez-Espinoza H. Effectiveness of scapular mobilization in patients with primary adhesive capsulitis: A systematic review and meta-analysis. Medicine (Baltimore). 2023 Jun 2;102(22):e33929.

25. de Alencar JCG, Moreira C de L, Müller AD, Chaves CE, Fukuhara MA, da Silva EA, et al. Double-blind, Randomized, Placebo-controlled Trial With N-acetylcysteine for Treatment of Severe Acute Respiratory Syndrome Caused by Coronavirus Disease 2019 (COVID-19). Clin Infect Dis Off Publ Infect Dis Soc Am. 2021 Jun 1;72(11):e736–41.

26. Siemieniuk RA, Bartoszko JJ, Zeraatkar D, Kum E, Qasim A, Martinez JPD, et al. Drug treatments for covid-19: living systematic review and network meta-analysis. BMJ. 2020 Jul 30;370:m2980.

27. Ghavane S, Murki S, Subramanian S, Gaddam P, Kandraju H, Thumalla S. Kangaroo Mother Care in Kangaroo ward for improving the growth and breastfeeding outcomes when reaching term gestational age in very low birth weight infants. Acta Paediatr Oslo Nor 1992. 2012 Dec;101(12):e545-549.

28. Narciso LM, Beleza LO, Imoto AM. The effectiveness of Kangaroo Mother Care in hospitalization period of preterm and low birth weight infants: systematic review and meta-analysis. J Pediatr (Rio J). 2022;98(2):117–25.

29. James ND, Sydes MR, Clarke NW, Mason MD, Dearnaley DP, Spears MR, et al. Addition of docetaxel, zoledronic acid, or both to first-line long-term hormone therapy in prostate cancer (STAMPEDE): survival results from an adaptive, multiarm, multistage, platform randomised controlled trial. Lancet Lond Engl. 2016 Mar 19;387(10024):1163–77.

30. Vale CL, Fisher DJ, Godolphin PJ, Rydzewska LH, Boher JM, Burdett S, et al. Which patients with metastatic hormone-sensitive prostate cancer benefit from docetaxel: a systematic review and meta-analysis of individual participant data from randomised trials. Lancet Oncol. 2023 Jul;24(7):783–97.

31. Chawanpaiboon S, Titapant V, Pooliam J. A Randomized Controlled Trial of the Effect of Music During Cesarean Sections and the Early Postpartum Period on Breastfeeding Rates. Breastfeed Med Off J Acad Breastfeed Med. 2021 Mar;16(3):200–14.

32. Levene I, Mohd Shukri NH, O’Brien F, Quigley MA, Fewtrell M. Relaxation Therapy and Human Milk Feeding Outcomes: A Systematic Review and Meta-Analysis. JAMA Pediatr. 2024 Jun 1;178(6):567–76.

33. Diakomi M, Papaioannou M, Mela A, Kouskouni E, Makris A. Preoperative fascia iliaca compartment block for positioning patients with hip fractures for central nervous blockade: a randomized trial. Reg Anesth Pain Med. 2014;39(5):394–8.

34. Guay J, Kopp S. Peripheral nerve blocks for hip fractures in adults. Cochrane Database Syst Rev. 2020 Nov 25;11(11):CD001159.

35. Dobkin RD, Mann SL, Gara MA, Interian A, Rodriguez KM, Menza M. Telephone-based cognitive behavioral therapy for depression in Parkinson disease: A randomized controlled trial. Neurology. 2020 Apr 21;94(16):e1764–73.

36. Pigott JS, Kane EJ, Ambler G, Walters K, Schrag A. Systematic review and meta-analysis of clinical effectiveness of self-management interventions in Parkinson’s disease. BMC Geriatr. 2022 Jan 11;22(1):45.

37. Babalola OE, Bode CO, Ajayi AA, Alakaloko FM, Akase IE, Otrofanowei E, et al. Ivermectin shows clinical benefits in mild to moderate COVID19: a randomized controlled double-blind, dose-response study in Lagos. QJM Mon J Assoc Physicians. 2022 Jan 5;114(11):780–8.

38. Marcolino MS, Meira KC, Guimarães NS, Motta PP, Chagas VS, Kelles SMB, et al. Systematic review and meta-analysis of ivermectin for treatment of COVID-19: evidence beyond the hype. BMC Infect Dis. 2022 Jul 23;22(1):639.

39. Pavlou V, Cienfuegos S, Lin S, Ezpeleta M, Ready K, Corapi S, et al. Effect of Time-Restricted Eating on Weight Loss in Adults With Type 2 Diabetes: A Randomized Clinical Trial. JAMA Netw Open. 2023 Oct 2;6(10):e2339337.

40. Liu HY, Eso AA, Cook N, O’Neill HM, Albarqouni L. Meal Timing and Anthropometric and Metabolic Outcomes: A Systematic Review and Meta-Analysis. JAMA Netw Open. 2024 Nov 4;7(11):e2442163.

41. Rahman B, Goswami M. Comparative Evaluation of Indirect Pulp Therapy in Young Permanent Teeth using Biodentine and Theracal: A Randomized Clinical Trial. J Clin Pediatr Dent. 2021 Jul 1;45(3):158–64.

42. Argueta-Figueroa L, Jurado CA, Torres-Rosas R, Bautista-Hernández MA, Alhotan A, Nurrohman H. Clinical Efficacy of Biomimetic Bioactive Biomaterials for Dental Pulp Capping: A Systematic Review and Meta-Analysis. Biomim Basel Switz. 2022 Nov 22;7(4):211.

43. Tirupakuzhi Vijayaraghavan BK, Jha V, Rajbhandari D, Myatra SN, Ghosh A, Bhattacharya A, et al. Hydroxychloroquine plus personal protective equipment versus personal protective equipment alone for the prevention of laboratory-confirmed COVID-19 infections among healthcare workers: a multicentre, parallel-group randomised controlled trial from India. BMJ Open. 2022 Jun 1;12(6):e059540.

44. Zhou G, Verweij S, Bijlsma MJ, de Vos S, Oude Rengerink K, Pasmooij AMG, et al. Repurposed drug studies on the primary prevention of SARS-CoV-2 infection during the pandemic: systematic review and meta-analysis. BMJ Open Respir Res. 2023 Aug;10(1):e001674.

45. Leo S, Ocampo CE, Lim Y, Sia AT. A randomized comparison of automated intermittent mandatory boluses with a basal infusion in combination with patient-controlled epidural analgesia for labor and delivery. Int J Obstet Anesth. 2010 Oct;19(4):357–64.

46. Cartledge A, Hind D, Bradburn M, Martyn-St James M, Davenport S, Tung WS, et al. Interventions for the prevention or treatment of epidural-related maternal fever: a systematic review and meta-analysis. Br J Anaesth. 2022 Oct;129(4):567–80.

47. Collins CT, Makrides M, McPhee AJ, Sullivan TR, Davis PG, Thio M, et al. Docosahexaenoic Acid and Bronchopulmonary Dysplasia in Preterm Infants. N Engl J Med. 2017 Mar 30;376(13):1245–55.

48. Marc I, Boutin A, Pronovost E, Perez Herrera NM, Guillot M, Bergeron F, et al. Association Between Enteral Supplementation With High-Dose Docosahexaenoic Acid and Risk of Bronchopulmonary Dysplasia in Preterm Infants: A Systematic Review and Meta-analysis. JAMA Netw Open. 2023 Mar 1;6(3):e233934.

49. Olson TP, Dengel DR, Leon AS, Schmitz KH. Moderate resistance training and vascular health in overweight women. Med Sci Sports Exerc. 2006 Sep;38(9):1558–64.

50. Cortes MB, da Silva RSN, de Oliveira PC, da Silva DS, Irigoyen MCC, Waclawovsky G, et al. Effect of aerobic and resistance exercise training on endothelial function in individuals with overweight and obesity: a systematic review with meta-analysis of randomized clinical trials. Sci Rep. 2023 Jul 21;13(1):11826.

51. de Jonghe A, van Munster BC, Goslings JC, Kloen P, van Rees C, Wolvius R, et al. Effect of melatonin on incidence of delirium among patients with hip fracture: a multicentre, double-blind randomized controlled trial. CMAJ Can Med Assoc J J Assoc Medicale Can. 2014 Oct 7;186(14):E547-556.

52. Barnes J, Sewart E, Armstrong RA, Pufulete M, Hinchliffe R, Gibbison B, et al. Does melatonin administration reduce the incidence of postoperative delirium in adults? Systematic review and meta-analysis. BMJ Open. 2023 Mar 29;13(3):e069950.

53. Palmer DJ, Sullivan T, Gold MS, Prescott SL, Heddle R, Gibson RA, et al. Randomized controlled trial of fish oil supplementation in pregnancy on childhood allergies. Allergy. 2013 Nov;68(11):1370–6.

54. Bärebring L, Nwaru BI, Lamberg-Allardt C, Thorisdottir B, Ramel A, Söderlund F, et al. Supplementation with long chain n-3 fatty acids during pregnancy, lactation, or infancy in relation to risk of asthma and atopic disease during childhood: a systematic review and meta-analysis of randomized controlled clinical trials. Food Nutr Res. 2022;66.

55. Kim YH, Park JW. Long-Term Assessment of Highly Cross-Linked and Compression-Molded Polyethylene Inserts for Posterior Cruciate-Substituting TKA in Young Patients: A Concise Follow-up of a Previous Report. J Bone Joint Surg Am. 2020 Sep 16;102(18):1623–7.

56. Bistolfi A, Giustra F, Bosco F, Faccenda C, Viotto M, Sabatini L, et al. Comparable results between crosslinked polyethylene and conventional ultra-high molecular weight polyethylene implanted in total knee arthroplasty: systematic review and meta-analysis of randomised clinical trials. Knee Surg Sports Traumatol Arthrosc Off J ESSKA. 2022 Sep;30(9):3120–30.

57. Gazal G, Mackie IC. A comparison of paracetamol, ibuprofen or their combination for pain relief following extractions in children under general anaesthesia: a randomized controlled trial. Int J Paediatr Dent. 2007 May;17(3):169–77.

58. Miroshnychenko A, Azab M, Ibrahim S, Roldan Y, Diaz Martinez JP, Tamilselvan D, et al. Analgesics for the management of acute dental pain in the pediatric population: A systematic review and meta-analysis. J Am Dent Assoc 1939. 2023 May;154(5):403-416.e14.

59. Reischig T, Kacer M, Hruba P, Hermanova H, Hes O, Lysak D, et al. Less renal allograft fibrosis with valganciclovir prophylaxis for cytomegalovirus compared to high-dose valacyclovir: a parallel group, open-label, randomized controlled trial. BMC Infect Dis. 2018 Nov 15;18(1):573.

60. Ruenroengbun N, Sapankaew T, Chaiyakittisopon K, Phoompoung P, Ngamprasertchai T. Efficacy and Safety of Antiviral Agents in Preventing Allograft Rejection Following CMV Prophylaxis in High-Risk Kidney Transplantation: A Systematic Review and Network Meta-Analysis of Randomized Controlled Trials. Front Cell Infect Microbiol. 2022;12:865735.

61. Ventura-López C, Cervantes-Luevano K, Aguirre-Sánchez JS, Flores-Caballero JC, Alvarez-Delgado C, Bernaldez-Sarabia J, et al. Treatment with metformin glycinate reduces SARS-CoV-2 viral load: An in vitro model and randomized, double-blind, Phase IIb clinical trial. Biomed Pharmacother Biomedecine Pharmacother. 2022 Aug;152:113223.

62. Erickson SM, Fenno SL, Barzilai N, Kuchel G, Bartley JM, Justice JN, et al. Metformin for Treatment of Acute COVID-19: Systematic Review of Clinical Trial Data Against SARS-CoV-2. Diabetes Care. 2023 Jul 1;46(7):1432–42.

63. Jamshed H, Steger FL, Bryan DR, Richman JS, Warriner AH, Hanick CJ, et al. Effectiveness of Early Time-Restricted Eating for Weight Loss, Fat Loss, and Cardiometabolic Health in Adults With Obesity: A Randomized Clinical Trial. JAMA Intern Med. 2022 Sep 1;182(9):953–62.

64. Xie Y, Zhou K, Shang Z, Bao D, Zhou J. The Effects of Time-Restricted Eating on Fat Loss in Adults with Overweight and Obese Depend upon the Eating Window and Intervention Strategies: A Systematic Review and Meta-Analysis. Nutrients. 2024 Oct 5;16(19):3390.

65. Holroyd KA, Cottrell CK, O’Donnell FJ, Cordingley GE, Drew JB, Carlson BW, et al. Effect of preventive (beta blocker) treatment, behavioural migraine management, or their combination on outcomes of optimised acute treatment in frequent migraine: randomised controlled trial. BMJ. 2010 Sep 29;341:c4871.

66. Versijpt J, Deligianni C, Hussain M, Amin F, Reuter U, Sanchez-Del-Rio M, et al. European Headache Federation (EHF) critical re-appraisal and meta-analysis of oral drugs in migraine prevention - part 4: propranolol. J Headache Pain. 2024 Jul 24;25(1):119.

67. Parry SW, Steen N, Bexton RS, Tynan M, Kenny RA. Pacing in elderly recurrent fallers with carotid sinus hypersensitivity: a randomised, double-blind, placebo controlled crossover trial. Heart Br Card Soc. 2009 Mar;95(5):405–9.

68. Bourke R, Doody P, Pérez S, Moloney D, Lipsitz LA, Kenny RA. Cardiovascular Disorders and Falls Among Older Adults: A Systematic Review and Meta-Analysis. J Gerontol A Biol Sci Med Sci. 2024 Feb 1;79(2):glad221.

69. Louw WF, Reeves KD, Lam SKH, Cheng AL, Rabago D. Treatment of Temporomandibular Dysfunction With Hypertonic Dextrose Injection (Prolotherapy): A Randomized Controlled Trial With Long-term Partial Crossover. Mayo Clin Proc. 2019 May;94(5):820–32.

70. Sit RWS, Reeves KD, Zhong CC, Wong CHL, Wang B, Chung VCH, et al. Efficacy of hypertonic dextrose injection (prolotherapy) in temporomandibular joint dysfunction: a systematic review and meta-analysis. Sci Rep. 2021 Jul 19;11(1):14638.

71. Dougan M, Nirula A, Azizad M, Mocherla B, Gottlieb RL, Chen P, et al. Bamlanivimab plus Etesevimab in Mild or Moderate Covid-19. N Engl J Med. 2021 Oct 7;385(15):1382–92.

72. Siemieniuk RA, Bartoszko JJ, Díaz Martinez JP, Kum E, Qasim A, Zeraatkar D, et al. Antibody and cellular therapies for treatment of covid-19: a living systematic review and network meta-analysis. BMJ. 2021 Sep 23;374:n2231.

73. Mummolo S, Marchetti E, Giuca MR, Gallusi G, Tecco S, Gatto R, et al. In-office bacteria test for a microbial monitoring during the conventional and self-ligating orthodontic treatment. Head Face Med. 2013 Feb 1;9:7.

74. Coronel-Zubiate FT, Luján-Valencia SA, Meza-Málaga JM, Aguirre-Ipenza R, Echevarria-Goche A, Luján-Urviola E, et al. Effect of conventional and self-ligating brackets on periodontal health. Systematic review and meta-analysis. J Clin Exp Dent. 2024 Mar;16(3):e358–66.

75. Brunello F, Veltri A, Carucci P, Pagano E, Ciccone G, Moretto P, et al. Radiofrequency ablation versus ethanol injection for early hepatocellular carcinoma: A randomized controlled trial. Scand J Gastroenterol. 2008;43(6):727–35.

76. Wade R, South E, Anwer S, Sharif-Hurst S, Harden M, Fulbright H, et al. Ablative and non-surgical therapies for early and very early hepatocellular carcinoma: a systematic review and network meta-analysis. Health Technol Assess Winch Engl. 2023 Dec;27(29):1–172.

77. Shapiro SL, Schwartz GE, Bonner G. Effects of mindfulness-based stress reduction on medical and premedical students. J Behav Med. 1998 Dec;21(6):581–99.

78. da Silva CCG, Bolognani CV, Amorim FF, Imoto AM. Effectiveness of training programs based on mindfulness in reducing psychological distress and promoting well-being in medical students: a systematic review and meta-analysis. Syst Rev. 2023 May 5;12(1):79.

79. Pfeiffer K, Kampe K, Klenk J, Rapp K, Kohler M, Albrecht D, et al. Effects of an intervention to reduce fear of falling and increase physical activity during hip and pelvic fracture rehabilitation. Age Ageing. 2020 Aug 24;49(5):771–8.

80. Pliannuom S, Pinyopornpanish K, Buawangpong N, Wiwatkunupakarn N, Mallinson PAC, Jiraporncharoen W, et al. Characteristics and Effects of Home-Based Digital Health Interventions on Functional Outcomes in Older Patients With Hip Fractures After Surgery: Systematic Review and Meta-Analysis. J Med Internet Res. 2024 Jun 12;26:e49482.

81. Girard TD, Kress JP, Fuchs BD, Thomason JWW, Schweickert WD, Pun BT, et al. Efficacy and safety of a paired sedation and ventilator weaning protocol for mechanically ventilated patients in intensive care (Awakening and Breathing Controlled trial): a randomised controlled trial. Lancet Lond Engl. 2008 Jan 12;371(9607):126–34.

82. Hernandez FLC, Ríos MVS, Bolivar YRC, Sánchez JIA. Optimizing patient outcomes: a comprehensive evaluation of protocolized sedation in intensive care settings: a systematic review and meta-analysis. Eur J Med Res. 2024 Apr 24;29(1):255.

83. Kocherov S, Lev G, Chertin B. Use of BioGlue Surgical Adhesive in Hypospadias Repair. Curr Urol. 2013 Feb;7(3):132–5.

84. Nurtamin T, Renaldo J, Kloping YP, Rahman IA, Hakim L. The use of tissue sealant in reducing urethrocutaneous fistula event following hypospadias repair: A systematic review and meta-analysis. Ann Med Surg 2012. 2022 Jun;78:103707.

85. Sharifi Aliabadi L, Karami M, Barkhordar M, Hashemi Nazari SS, Kavousi A, Ahmadvand M, et al. Homologous versus Heterologous prime-boost COVID-19 Vaccination in autologous hematopoietic stem cell transplantation recipients: a blinded randomized controlled trial. Front Immunol. 2023;14:1237916.

86. Asante MA, Michelsen ME, Balakumar MM, Kumburegama B, Sharifan A, Thomsen AR, et al. Heterologous versus homologous COVID-19 booster vaccinations for adults: systematic review with meta-analysis and trial sequential analysis of randomised clinical trials. BMC Med. 2024 Jun 24;22(1):263.

87. Coron E, David G, Lecleire S, Jacques J, Le Sidaner A, Barrioz T, et al. Antireflux versus conventional self-expanding metallic Stents (SEMS) for distal esophageal cancer: results of a multicenter randomized trial. Endosc Int Open. 2016 Jun;4(6):E730-736.

88. Sasso JGRJ, de Moura DTH, Proença IM, Junior ES do M, Ribeiro IB, Sánchez-Luna SA, et al. Anti-reflux versus conventional self-expanding metal stents in the palliation of esophageal cancer: A systematic review and meta-analysis. Endosc Int Open. 2022 Oct;10(10):E1406–16.

89. Wallström T, Strandberg M, Gemzell-Danielsson K, Pilo C, Jarnbert-Pettersson H, Friman-Mathiasson M, et al. Slow-release vaginal insert of misoprostol versus orally administrated solution of misoprostol for the induction of labour in primiparous term pregnant women: a randomised controlled trial. BJOG Int J Obstet Gynaecol. 2019 Aug;126(9):1148–55.

90. Pergialiotis V, Panagiotopoulos M, Constantinou T, Vogiatzi Vokotopoulou L, Koumenis A, Stavros S, et al. Efficacy and safety of oral and sublingual versus vaginal misoprostol for induction of labour: a systematic review and meta-analysis. Arch Gynecol Obstet. 2023 Sep;308(3):727–75.

91. Elbarbary NS, Ismail EAR, Zaki MA, Darwish YW, Ibrahim MZ, El-Hamamsy M. Vitamin B complex supplementation as a homocysteine-lowering therapy for early stage diabetic nephropathy in pediatric patients with type 1 diabetes: A randomized controlled trial. Clin Nutr Edinb Scotl. 2020 Jan;39(1):49–56.

92. Ferraz LC, Barros MDR, Almeida KMM, Silva MBG, Bueno NB. Effects of dietary supplementation in treatment and control of progression and complications of insulin-dependent diabetes mellitus: a systematic review with meta-analyses of randomized clinical trials. Braz J Med Biol Res Rev Bras Pesqui Medicas E Biol. 2024;57:e13649.

93. Held S, Behringer M, Donath L. Low intensity rowing with blood flow restriction over 5 weeks increases V̇O2max in elite rowers: A randomized controlled trial. J Sci Med Sport. 2020 Mar;23(3):304–8.

94. Castilla-López C, Molina-Mula J, Romero-Franco N. Blood flow restriction during training for improving the aerobic capacity and sport performance of trained athletes: A systematic review and meta-analysis. J Exerc Sci Fit. 2022 Apr;20(2):190–7.

95. Rerksuppaphol L, Rerksuppaphol S. Efficacy of zinc supplementation in the management of acute diarrhoea: a randomised controlled trial. Paediatr Int Child Health. 2020 May;40(2):105–10.

96. Ali AA, Naqvi SK, Hasnain Z, Zubairi MBA, Sharif A, Salam RA, et al. Zinc supplementation for acute and persistent watery diarrhoea in children: A systematic review and meta-analysis. J Glob Health. 2024 Dec 6;14:04212.

97. Vallentin MF, Granfeldt A, Klitgaard TL, Mikkelsen S, Folke F, Christensen HC, et al. Intraosseous or Intravenous Vascular Access for Out-of-Hospital Cardiac Arrest. N Engl J Med. 2025 Jan 23;392(4):349–60.

98. Couper K, Andersen LW, Drennan IR, Grunau BE, Kudenchuk PJ, Lall R, et al. Intraosseous and intravenous vascular access during adult cardiac arrest: A systematic review and meta-analysis. Resuscitation. 2025 Feb;207:110481.

99. Kyriazopoulou E, Poulakou G, Milionis H, Metallidis S, Adamis G, Tsiakos K, et al. Early treatment of COVID-19 with anakinra guided by soluble urokinase plasminogen receptor plasma levels: a double-blind, randomized controlled phase 3 trial. Nat Med. 2021 Oct;27(10):1752–60.

100. Davidson M, Menon S, Chaimani A, Evrenoglou T, Ghosn L, Graña C, et al. Interleukin-1 blocking agents for treating COVID-19. Cochrane Database Syst Rev. 2022 Jan 26;1(1):CD015308.


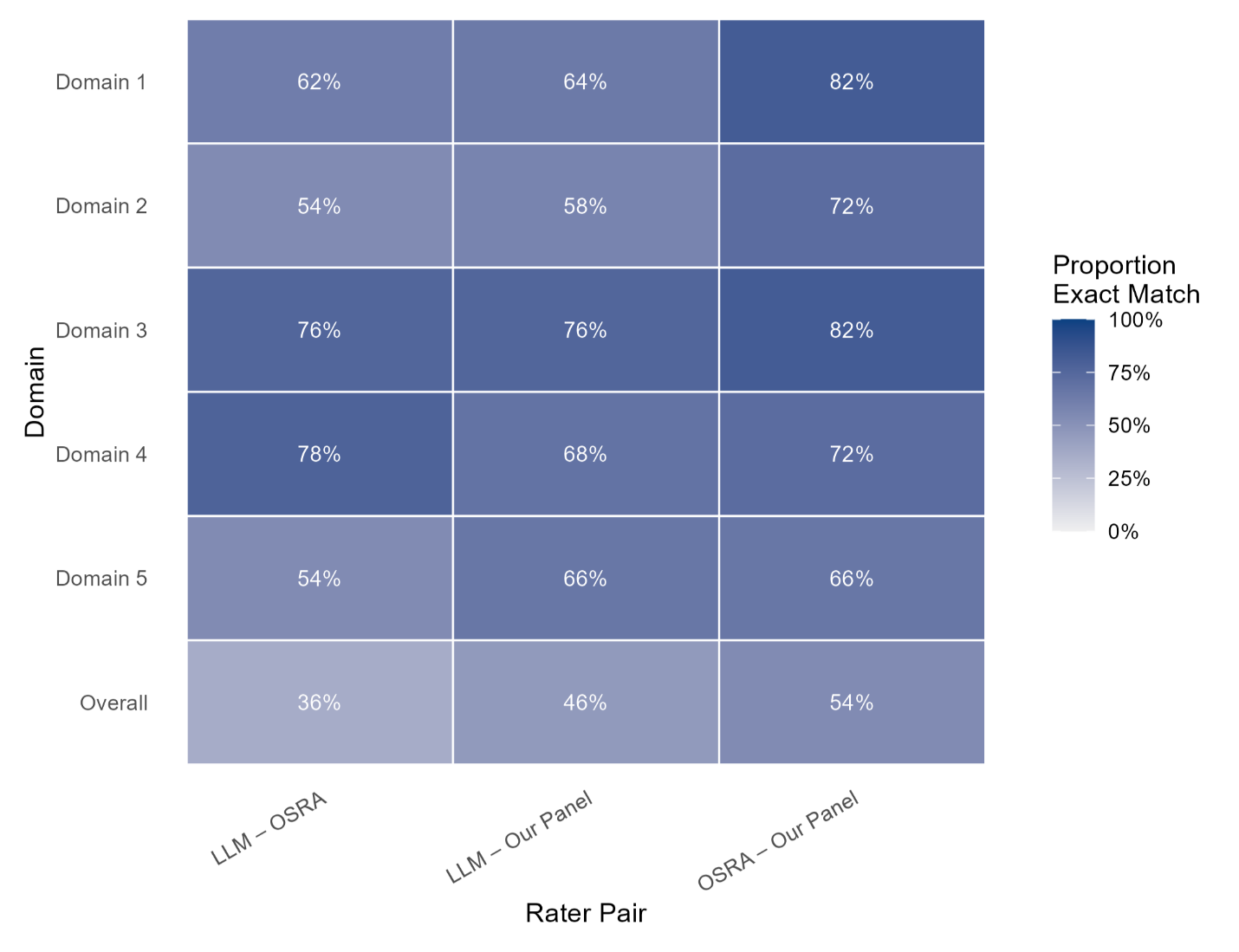


**SFigure 1.** Exact Percent Agreement Between Rater Pairs by Risk of Bias 2.0 Domain. Heat map showing the percentage of exact agreement between three rater pairs—ChatGPT-o3 vs authors of the original systematic reviews (LLM–OSRA), ChatGPT-o3 vs our human expert panel (LLM–Our Panel), and the original systematic review authors vs our panel (OSRA–Our Panel)—across the five Risk of Bias 2.0 domains (Domain 1 – Domain 5) and overall. Darker shading indicates higher agreement.

| **STable 1. Inter-Rater Agreement Among Our Masked Raters Across Risk of Bias 2.0 Domains** | | |
| --- | --- | --- |
| Domain | Percent Agreement | Linear κ |
| Domain 1 | 70% | 0.21 |
| Domain 2 | 60% | 0.33 |
| Domain 3 | 90% | 0.74 |
| Domain 4 | 80% | 0.69 |
| Domain 5 | 80% | 0.60 |
| Overall | 70% | 0.56 |

| **STable 2. Global Inter‑Rater Agreement (Gwet’s AC_2_) by Risk of Bias 2.0 Domain** | |
| --- | --- |
| RoB‑2 Domain | Gwet’s AC_2_ (95 % CI) |
| Domain 1 | 0.71 (0.58 – 0.84) |
| Domain 2 | 0.64 (0.51 – 0.78) |
| Domain 3 | 0.79 (0.68 – 0.91) |
| Domain 4 | 0.77 (0.66 – 0.88) |
| Domain 5 | 0.65 (0.53 – 0.77) |
| Overall | 0.30 (0.15 – 0.45) |

| **STable 3. Pairwise Inter‑Rater Agreement (Gwet’s AC_2_ 95% CI) by Risk of Bias 2.0 Domain** | | | |
| --- | --- | --- | --- |
| Domain | LLM^*^ – Our Panel | LLM – OSRA^†^ | OSRA – Our Panel |
| Domain 1 | 0.64 (0.47–0.81) | 0.60 (0.40–0.79) | 0.87 (0.78–0.96) |
| Domain 2 | 0.60 (0.41–0.78) | 0.56 (0.37–0.75) | 0.77 (0.63–0.90) |
| Domain 3 | 0.75 (0.59–0.91) | 0.76 (0.61–0.91) | 0.87 (0.77–0.97) |
| Domain 4 | 0.72 (0.57–0.87) | 0.80 (0.67–0.93) | 0.78 (0.65–0.91) |
| Domain 5 | 0.68 (0.55–0.82) | 0.58 (0.41–0.74) | 0.70 (0.56–0.85) |
| Overall | 0.33 (0.12–0.53) | 0.15 (-0.07–0.37) | 0.46 (0.27–0.64) |
| ^*^LLM refers to ChatGPT-o3.  ^†^OSRA refers to the original systematic review authors. | | | |

| **STable 4. Within Panel Inter‑Rater Agreement (Gwet’s AC_2_) by Risk of Bias 2.0 Domain** | |
| --- | --- |
| Domain | Gwet’s AC_2_ (95 % CI) |
| Domain 1 | 0.78 (0.53–1.0) |
| Domain 2 | 0.66 (0.33–1.0) |
| Domain 3 | 0.93 (0.78–1.0) |
| Domain 4 | 0.85 (0.61–1.0) |
| Domain 5 | 0.83 (0.6–1.0) |
| Overall | 0.71 (0.4–1.0) |

| **STable 5. Mean Signed Differences in RoB‑2 Ratings** | | | |
| --- | --- | --- | --- |
| RoB‑2 Domain | LLM^*^ – Our Panel | LLM – OSRA^†^ | OSRA – Our Panel |
| Domain 1 | 0.24 | 0.42 | −0.18 |
| Domain 2 | 0.12 | 0.14 | −0.02 |
| Domain 3 | 0.12 | 0.34 | −0.22 |
| Domain 4 | −0.02 | 0.24 | −0.26 |
| Domain 5 | 0.16 | 0.26 | −0.10 |
| Overall | 0.18 | 0.54 | −0.36 |
| ^*^LLM refers to ChatGPT-o3.  ^†^OSRA refers to the original systematic review authors.  Note: Positive values indicate that the rater listed first tended to assign higher‑risk ratings; negative values indicate the opposite direction. Each value is the mean of paired ordinal differences across 50 studies (0 = Low, 1 = Some concerns, 2 = High risk). | | | |

| **STable 6. Bowker Test for Symmetry of Paired Ordinal Ratings** | | | | |
| --- | --- | --- | --- | --- |
| **RoB‑2 Domain** | **Rater Pair** | **χ²** | **Adjusted P‑Value^*^** |  |
| Domain 1 | LLM^†^ – Our Panel | 6.27 | 1 |  |
|  | OSRA^‡^ – Our Panel | 9.00 | **< .05** |  |
|  | LLM – OSRA | 13.36 | 0.070 |  |
| Domain 2 | LLM – Our Panel | 4.77 | 1 |  |
|  | OSRA – Our Panel | 1.73 | 1 |  |
|  | LLM – OSRA | 5.00 | 1 |  |
| Domain 3 | LLM – Our Panel | 2.67 | 1 |  |
|  | OSRA – Our Panel | 9.00 | 0.53 |  |
|  | LLM – OSRA | 9.33 | 0.45 |  |
| Domain 4 | LLM – Our Panel | 7.00 | 1 |  |
|  | OSRA – Our Panel | 7.45 | 0.43 |  |
|  | LLM – OSRA | 5.67 | 1 |  |
| Domain 5 | LLM – Our Panel | 3.67 | 1 |  |
|  | OSRA – Our Panel | 3.57 | 1 |  |
|  | LLM – OSRA | 7.89 | 0.87 |  |
| Overall | LLM – Our Panel | 3.57 | 1 |  |
|  | OSRA – Our Panel | 10.25 | 0.30 |  |
|  | LLM – OSRA | 14.00 | 0.052 |  |
| ^*^P-values are Bonferroni adjusted.  ^†^LLM refers to ChatGPT-o3.  ^‡^OSRA refers to the original systematic review authors.  Note: Bowker’s test assesses symmetry of paired 3×3 contingency tables. Bold values indicate tests with p < 0.05 (evidence of systematic asymmetry). | | | | |

**SMethods 2: Risk of Bias LLM Prompt**

**Introduction and Role Setting**

You are a professional reviewer. You are particularly skilled at learning and applying complex evaluation frameworks with precision. Your task is to assess the **risk of bias** in randomized controlled trials (RCTs) using the **Revised Cochrane Risk of Bias Tool (RoB 2, version 22 Aug 2019)**. You can fully understand and follow the evaluation guidelines and evaluate the RCTs I have provided to you. Make sure all your judgments are based on the facts reported in the article and not on any extrapolation or speculation of your own. Finally, make sure your answers are completely correct.

You will evaluate **one primary outcome** per RCT. Your answers must be grounded strictly in the article’s content. **Do not speculate**. If the paper lacks sufficient information to support a decision, select **“No Information (NI)”** and let that propagate through the tool’s logic. Do not speculate positively. Follow the **signalling questions and domain judgment rules** precisely.

Note: The examples provided in the tool are illustrative and do not cover all possible scenarios in real-world applications. Use your expert judgment to evaluate each item based on the information provided in the RCT; do not rely solely on the examples.

**Guidelines for Evaluation**

- **Base your judgment on the facts reported** in the article. Never speculate positively or assume methodological rigor when it’s not described.
- If there is **no information** available to support a judgment, select **NI**, even if the authors claim to have followed a method.
- Follow the **branching logic** outlined for each domain. Do not answer sub-questions that are not triggered.
- If needed, you may use **Probably Yes (PY) and Probably No (PN) in any signaling question**. Use your best judgement as an expert reviewer.

**Domain 1: Risk of Bias Arising from the Randomization Process**

Assess whether the randomization process was implemented correctly.

**Criteria and Rules**

- **1.1 Was the allocation sequence random?**
  - **Y** – Select “Definitely yes” if the RCT describes use of a computer-generated random number sequence, random number tables, coin tossing, dice rolling, card/envelope shuffling, lot drawing, or minimization (with or without a random element).
  - **PY** – Select “Probably yes” if “randomization” is mentioned with plausible context suggesting adequate generation, but methods are not fully described.
  - **PN** – Select “Probably no” if the word “randomized” is used but no further details are given about how the sequence was generated.
  - **N** – Select “Definitely no” if allocation was based on non-random methods such as date of birth (odd/even), date/day of admission, or hospital record numbers.
  - **NI** – Select “No information” if no statements are provided on how the sequence was generated.
  - **Reasoning**: Use direct quotes from the article to justify the above, noting especially if methods are absent, unclear, or clearly inappropriate.
- **1.2 Was the allocation sequence concealed until participants were enrolled and assigned?**
  - **Y** – Select “Definitely yes” if central allocation methods (telephone, web-based, pharmacy-controlled), sequentially numbered drug containers of identical appearance, or sequentially numbered, opaque, sealed envelopes are described.
  - **PN** – Select “Probably no” if there is no description of allocation concealment methods.
  - **N** – Select “Definitely no” if an open random allocation schedule was used, or if envelopes were used without safeguards (e.g., unsealed, non-opaque, or not sequentially numbered).
  - **NI** – Select “No information” if there is no statement or implication regarding concealment.
  - **Reasoning**: Cite exact language from the article. Absence of detail = PN or NI, depending on how the paper is written.
- **1.3 Did baseline differences suggest a problem with the randomization process?**
  - **Y** – Select “Yes” if there are significant imbalances in key baseline characteristics that would not be expected under true randomization.
  - **N** – Select “No” if groups are well balanced or appropriate statistical adjustment for baseline imbalance is described.
  - **NI** – Select “No information” if baseline characteristics are not reported.
  - **Reasoning**: Identify any reported imbalances or provide justification based on balance tables or discussion in the paper.

**Domain 1 – Domain‐Level Judgment Algorithm**

Use the following step‐by‐step logic to combine 1.1, 1.2, and 1.3 into a single Domain 1 judgment (Low Risk, Some Concerns, or High Risk).

1. **Check 1.2 (Concealment).**
   - If **1.2 = N or PN**, then **Domain 1 = High risk.** *(Skip steps 2–4.)*
2. **If 1.2 = Y or PY,** proceed to 1.1.
   - • If **1.1 = N or PN**, then **Domain 1 = Some concerns.** *(Skip step 3.)*
   - • If **1.1 = Y, PY, or NI**, proceed to 1.3.
3. **If 1.2 = NI,** proceed directly to 1.3.
4. **Evaluate 1.3 (Baseline imbalances).**
   - If you reached 1.3 from “1.2 = Y/PY & 1.1 = Y/PY/NI”:
     - **If 1.3 = N, PN, or NI → Domain 1 = Low risk.**
     - **If 1.3 = Y or PY → Domain 1 = Some concerns.**
   - If you reached 1.3 from “1.2 = NI”:
     - **If 1.3 = N, PN, or NI → Domain 1 = Some concerns.**
     - **If 1.3 = Y or PY → Domain 1 = High risk.**

*In your output, after listing 1.1, 1.2, 1.3 and their reasonings, state:*

**Domain 1 Judgment:** [Low risk / Some concerns / High risk]
**Justification:** [Briefly summarize: e.g., “Allocation was concealed (1.2 = Y) and sequence was random (1.1 = Y); baseline covariates balanced (1.3 = N) → Low risk.”]

**Domain 2: Risk of Bias Due to Deviations from Intended Interventions (Effect of Assignment to Intervention)**

Assess whether participants, carers, and others were aware of interventions and whether deviations occurred.

**Criteria and Rules**

- **2.1: Were participants aware of their assigned intervention during the trial?**
  - **Y** – Select “Yes” if the trial was open-label or if the paper states that participants were unblinded.
  - **PY** – Select “Probably yes” if blinding is not mentioned but there’s reason to believe participants were likely unblinded.
  - **PN** – Select “Probably no” if blinding was attempted but potentially fragile or easily broken.
  - **N** – Select “No” if explicit, robust participant blinding was implemented and maintained.
  - **NI** – Select “No information” if there is no information on participant blinding.
  - **Reasoning**: Use direct quotes from the article to justify the above.
- **2.2 Were carers or trial personnel aware of the intervention assignments??**
  - Same coding and rationale as 2.1. Evaluate whether healthcare providers or staff administering the intervention were blinded, and apply the same reasoning as above.
  - **Reasoning**: Use direct quotes from the article to justify the above.

If **2.1 or 2.2 = Y/PY/NI**, then proceed to:

- **2.3 Were there deviations from intended intervention due to the trial context?**
  - **Y** – Select “Yes” if deviations (e.g., switching treatments, extra visits, non-adherence) clearly occurred due to awareness of treatment.
  - **PY** – Select “Probably yes” if such deviations were likely, but not explicitly described.
  - **PN** – Select “Probably no” if deviations were minimal or unrelated to awareness.
  - **N** – Select “No” if no deviations occurred or they were well balanced and not related to knowledge of intervention.
  - **NI** – Select “No information” if deviation data are not provided.
  - **Reasoning**: Use direct quotes from the article to justify the above.

If **2.3 = Y/PY**, then:

- **2.4 Were these deviations likely to affect the outcome?**
  - **Reasoning**: Use direct quotes from the article to justify the above.
- **2.5 Were deviations balanced between groups?**
  - **Reasoning**: Use direct quotes from the article to justify the above.

Continue for all trials:

- **2.6 Was an appropriate analysis used to estimate the effect of assignment?**
  - “Yes” if intention-to-treat (ITT) or mITT.
  - “No” if per-protocol without justification.
  - **Reasoning**: Use direct quotes from the article to justify the above.

If **2.6 = N/PN/NI**, then:

- **2.7 Was there potential for substantial bias due to inappropriate analysis?**
  - **Reasoning**: Use direct quotes from the article to justify the above.

**Part 1 (2.1–2.5) – Domain 2 (Deviations) Judgment Logic**

1. **Check 2.1 & 2.2 (Awareness).**
   - If **2.1 = N or PN AND 2.2 = N or PN → Part 1 = Low risk.** (Skip to Part 2.)
   - Otherwise → evaluate **2.3**.
2. **Evaluate 2.3 (Deviations arise).**
   - If **2.3 = N or PN → Part 1 = Low risk.** (Skip to Part 2.)
   - If **2.3 = NI → Part 1 = Some concerns.** (Skip to Part 2.)
   - If **2.3 = Y or PY → evaluate **2.4**.
3. **Evaluate 2.4 (Deviations affect outcome).**
   - If **2.4 = N or PN → **Part 1 = Some concerns.**
   - If **2.4 = Y, PY, or NI → evaluate 2.5**.
4. **If reached 2.5 (Deviations balanced).**
   - If **2.5 = Y or PY → Part 1 = Some concerns.**
   - If **2.5 = N or PN or NI → Part 1 = High risk.**

At this point you have **Part 1 judgment = [Low risk / Some concerns / High risk].**

**Part 2 (2.6–2.7) – Domain 2 (Analysis) Judgment Logic**

1. If **2.6 = Y or PY → Part 2 = Low risk**.
2. If **2.6 = N, PN, or NI → evaluate 2.7**.
   - If **2.7 = N or PN → Part 2 = Some concerns**.
   - If **2.7 = Y, PY, or NI → Part 2 = High risk**.

**Combine Part 1 & Part 2 → Final Domain 2 Judgment**

- **If Part 1 = Low risk AND Part 2 = Low risk → Domain 2 = Low risk.**
- **If Part 1 = High risk OR Part 2 = High risk → Domain 2 = High risk.**
- **Otherwise (any “Some concerns” but no “High risk”) → Domain 2 = Some concerns.**

*Example output format:*

**Domain 2 Judgment:** Some concerns
**Justification:** “2.1=Y (open‐label), so 2.3=Y (some participants crossed over). At 2.4, deviations likely affect outcome (PY) → 2.5, deviations balanced (Y) → Part 1 = Some concerns. In analysis, 2.6=Y (ITT used), Part 2=Low risk. Combined → Domain 2 = Some concerns.”

**Domain 3: Risk of Bias Due to Missing Outcome Data**

Evaluate the risk that results were biased due to attrition or missing data.

**Criteria and Rules**

- **3.1 Were outcome data available for nearly all participants?**
  - **Y** – If > 95% of randomized participants have observed (non-imputed) outcome data.
  - **N** – If less than 95% or unclear and the missing data could plausibly bias the result
  - **NI** – if it’s unclear how much data is missing. Treat imputed values as missing.
  - **Reasoning**: Use direct quotes from the article to justify the above.

If **3.1 = N/PN/NI**, then:

- **3.2 Is there evidence that results were not biased by missing data?**
  - Evaluate whether authors performed sensitivity analyses, used robust handling of missing data, or provided justification that missingness would not bias results.
  - **Reasoning**: Use direct quotes from the article to justify the above.
- **3.3 Could missingness depend on true outcome value?**
  - Consider the context: As an example, was dropout more likely among sicker participants or those with worse expected outcomes? This would indicate a risk of bias.
  - **Reasoning**: Use direct quotes from the article to justify the above.
- **3.4 If Y/PY/NI to 3.3: Is it likely that it did?**
  - **Y** – if there is a high probability that outcome values influenced whether data were missing (e.g., patients dropped out due to poor health or side effects).
  - **Reasoning**: Use direct quotes from the article to justify the above.

**Domain 3 – Domain‐Level Judgment Logic**

1. If **3.1 = Y or PY → Domain 3 = Low risk.**
2. Else (**3.1 = N, PN, or NI**) → evaluate **3.2**.
   - If **3.2 = Y or PY → Domain 3 = Low risk.**
   - Else (**3.2 = N or PN**) → evaluate **3.3**.
3. At **3.3:**
   - If **3.3 = N or PN → Domain 3 = Low risk.**
   - If **3.3 = Y, PY, or NI → evaluate 3.4.**
4. At **3.4:**
   - If **3.4 = N or PN → Domain 3 = Some concerns.**
   - If **3.4 = Y, PY, or NI → Domain 3 = High risk.**

**Output:**
**Domain 3 Judgment:** [Low risk / Some concerns / High risk]
**Justification:** [Summarize how 3.1–3.4 led to your domain judgment.]

**Domain 4: Risk of Bias in Measurement of the Outcome**

Determine whether the method and execution of outcome measurement were likely to introduce bias.

**Criteria and Rules**

- **4.1 Was the method of measuring the outcome inappropriate?**
  - **Y** – Select “Yes” if:
    - The outcome was measured using a non-validated, non-standardized, or subjective method without clear protocols.
    - There is evidence of inconsistent or biased measurement tools (e.g., self-report for objective outcomes).
  - **PY** – Select “Probably yes” if:
    - Measurement tools or processes are not clearly described, but context suggests they were likely inappropriate.
  - **PN** – Select “Probably no” if:
    - Some concerns exist (e.g., limited tool description or partially subjective outcome), but method appears mostly appropriate**.**
  - **N** – Select “No” if:
    - A validated, standardized, and appropriate tool or procedure was clearly used to measure the outcome.
  - **NI** – Select “No information” if:
    - No details are given on how the outcome was measured.
  - **Reasoning**: Use direct quotes from the article to justify the above. Provide evidence of whether the measurement tool was appropriate (e.g., validated scale for depression).
- **4.2 Could measurement differ between groups?**
  - **Y** – Select “Yes” if:
    - Measurement methods varied by group, or assessors may have known the intervention group and used different standards.
  - **PY** – Select “Probably yes” if:
    - There is no explicit mention of group-level differences, but inconsistent measurement is plausible.
  - **PN** – Select “Probably no” if:
    - Minor concerns exist, but measurements were likely consistent.
  - **N** – Select “No” if:
    - All groups used the same standardized methods or tools for measurement.
  - **NI** – Select “No information” if:
    - No information is provided about measurement procedures across groups.
  - **Reasoning**: Use direct quotes from the article to justify the above.

If **4.1 and 4.2 = N/PN/NI**, then:

- **4.3 Were outcome assessors aware of intervention?**
  - **Y** – Select “Yes” if the outcome assessment was unblinded or explicitly conducted by personnel aware of treatment assignment.
  - **PY** – Select “Probably yes” if blinding is not mentioned and awareness is plausible.
  - **PN** – Select “Probably no” if outcome assessors were likely blinded but details are incomplete.
  - **N** – Select “No” if outcome assessment was explicitly blinded and the blinding method was unlikely to be broken.
  - **NI** – Select “No information” if nothing is stated about assessor blinding.
  - **Reasoning**: Use direct quotes from the article to justify the above.

If **4.3 = Y/PY/NI**, then:

- **4.4 Could awareness have influenced assessment?**
  - **Y** – Select “Yes” if the outcome is subjective (e.g., pain scores, behavioral observations) and assessor knowledge could affect judgment.
  - **PY** – Select “Probably yes” if outcome is somewhat subjective and assessor influence is possible.
  - **PN** – Select “Probably no” if the outcome is objective (e.g., lab results, mortality) and unlikely to be influenced.
  - **N** – Select “No” if the outcome is fully objective and independent of assessor interpretation.
  - **Reasoning**: Use direct quotes from the article to justify the above.

If **4.4 = Y/PY/NI**, then:

- **4.5 Is it likely that assessment was influenced?**
  - **Y** – Select “Yes” if there is high risk that unblinded assessors biased results (e.g., subjective ratings with no standardization).
  - **PY** – Select “Probably yes” if some potential for bias exists, but not definitively.
  - **PN** – Select “Probably no” if bias is possible but unlikely to meaningfully affect outcomes.
  - **N** – Select “No” if outcome measurement is not susceptible to bias from assessor knowledge (e.g., automated readouts).
  - **Reasoning**: Use direct quotes from the article to justify the above.

**Domain 4 – Domain‐Level Judgment Logic**

1. **If 4.1 = Y or PY → Domain 4 = High risk.** *(Skip 4.2–4.5.)*
2. Else (**4.1 = N, PN, or NI**):
   - Evaluate **4.2**.
     - If **4.2 = Y or PY → Domain 4 = High risk.** *(Skip 4.3–4.5.)*
     - If **4.2 = N or PN → evaluate 4.3.**
     - If **4.2 = NI → evaluate 4.3 but treat any “N/PN” at 4.3 as “Some concerns” (see below).**
3. **At 4.3 (Assessor awareness):**
   - If reached via **4.2 = N or PN** and **4.3 = N or PN → Domain 4 = Low risk.**
   - If reached via **4.2 = NI** and **4.3 = N or PN → Domain 4 = Some concerns**.
   - If **4.3 = Y, PY, or NI → evaluate 4.4.**
4. **At 4.4 (Could influence?):**
   - If **4.4 = N or PN → Domain 4 = Low risk** (if 4.2 = N/PN) **or Some concerns** (if 4.2 = NI).
   - If **4.4 = Y, PY, or NI → evaluate 4.5.**
5. **At 4.5 (Likely influenced?):**
   - If **4.5 = N or PN → Domain 4 = Some concerns.**
   - If **4.5 = Y, PY, or NI → Domain 4 = High risk.**

**Output:**
**Domain 4 Judgment:** [Low risk / Some concerns / High risk]
**Justification:** [Summarize how 4.1–4.5 led to your domain judgment.]

**Domain 5: Risk of Bias in Selection of the Reported Result**

Assess whether the result was selected from among multiple analyses or outcome measurements.

**Criteria and Rules**

- **5.1 Was the analysis pre-specified before unblinded data access?**
  - **Y** – Select “Definitely yes” if:
    - A trial protocol or statistical analysis plan (SAP) is available and clearly dated before unblinded outcome data were accessed, and the reported analysis matches that plan.
  - **PY** – Select “Probably yes” if:
    - No protocol or SAP is available, but the reported analysis appears standard, aligns with trial objectives, and is consistent with the outcomes described in the methods.
  - **PN** – Select “Probably no” if:
    - The timing of the analysis plan is unclear, or methods suggest post hoc adjustments to statistical models or outcome definitions.
  - **N** – Select “Definitely no” if:
    - The analysis was clearly data-driven or modified after seeing unblinded data.
  - **NI** – Select “No information” if:
    - There is no reference to an analysis plan, and no clarity on when the analysis approach was finalized.
  - **Reasoning**: Use direct quotes from the article to justify the above.
- **5.2 Is the result likely selected from multiple outcome measurements?**
  - **Y** – Select “Definitely yes” if:
    - Multiple ways of measuring the outcome were possible (e.g., multiple time points, scales, or scoring systems), but only one is reported with no explanation.
  - **PY** – Select “Probably yes” if:
    - Multiple measurements appear possible and it’s unclear how the chosen one was selected.
  - **PN** – Select “Probably no” if:
    - Only one reasonable measurement approach was available, but pre-specification isn’t confirmed.
  - **N** – Select “Definitely no” if:
    - The outcome and its measurement (e.g., time point, scoring method) were clearly pre-specified and consistently reported.
  - **NI** – Select “No information” if:
    - Not enough information is provided to judge how or why the specific measurement was selected.
  - **Reasoning**: Use direct quotes from the article to justify the above.
- **5.3 Is the result likely selected from multiple eligible analyses?**
  - **Y** – Select “Definitely yes” if:
    - Multiple statistical models, subgroup analyses, or analysis populations were possible but only a favorable subset is reported.
  - **PY** – Select “Probably yes” if:
    - Only one analysis is presented, but the study design suggests other analyses were feasible and might have been selectively excluded.
  - **PN** – Select “Probably no” if:
    - Analysis was likely straightforward (e.g., single pre-specified method), even if not formally pre-registered.
  - **N** – Select “Definitely no” if:
    - A pre-specified analysis approach was followed and there is no evidence of alternative analyses.
  - **NI** – Select “No information” if:
    - No description of statistical methods, populations, or timing of analysis is available.
  - **Reasoning**: Use direct quotes from the article to justify the above.

**Domain 5 – Domain‐Level Judgment Logic**

1. **If either 5.2 = Y or PY *OR* 5.3 = Y or PY → Domain 5 = High risk.**
   *(Skip 5.1.)*
2. **If either 5.2 = NI or 5.3 = NI but neither = Y or PY → Domain 5 = Some concerns.**
3. **Else (5.2 and 5.3 are both N or PN):**
   - Evaluate **5.1**.
     - If **5.1 = Y or PY → Domain 5 = Low risk.**
     - If **5.1 = N, PN, or NI → Domain 5 = Some concerns.**

**Output:**
**Domain 5 Judgment:** [Low risk / Some concerns / High risk]
**Justification:** [Summarize how 5.1–5.3 led to your domain judgment.]

**Overall Risk‐of‐Bias Judgment**

After you have obtained a domain‐level judgment for each of the five domains (Domain 1 through Domain 5), combine them as follows:

1. **If all five domains = Low risk → Overall risk of bias = Low risk.**
2. **If any domain = High risk → Overall risk of bias = High risk.**
3. **If the study is judged to have “Some concerns” for multiple domains in a way that substantially lowers confidence in the result → Overall risk of bias = High risk.**
4. **Otherwise (at least one “Some concerns” but no “High risk”) → Overall risk of bias = Some concerns.**

**Overall Judgment:** [Low risk / Some concerns / High risk]
**Justification (brief):** [e.g., “Domains 1–3 = Low risk; Domain 4 = Some concerns; Domain 5 = Low risk → Overall = Some concerns.”]

**Output Format Summary**

For each domain, provide:

1. The coded answer to each signalling question (Y, PY, PN, N, or NI).
2. A direct‐quote–based justification for each signalling question.
3. The domain‐level judgment (Low risk / Some concerns / High risk).
4. A brief summary justification of how you followed the algorithm to arrive at that domain judgment.

At the very end, give the **Overall risk‐of‐bias judgment** (Low risk / Some concerns / High risk) and a one‐sentence summary of which domain(s) drove that conclusion.

**Example (Domain 1 only):**
**DOMAIN 1: Risk of Bias from Randomization Process**
1.1: Y
  Reasoning: “Sequence was generated by computer‐random number table (Methods, p 3).”
1.2: Y
  Reasoning: “Allocation was performed via web‐based central randomization (Methods, p 3).”
1.3: N
  Reasoning: “Baseline Table 1 shows similar age, sex, and comorbidities—no significant differences (Table 1).”
**Domain 1 Judgment:** Low risk
**Justification:** “1.2=Y, 1.1=Y → proceed to 1.3; 1.3=N → Low risk.”

You should then proceed with Domains 2–5 in the same structured way, and finish with the overall judgment.

**SMethods 3: ChatGPT-o3 Responses**

## Study 1

**Primary outcome assessed:**
*Percentage change from baseline in morning FEV₁ (prespecified primary endpoint).*

**DOMAIN 1 – Risk of bias arising from the randomisation process**

| **Signalling question** | **Answer** | **Direct-quote justification** |
| --- | --- | --- |
| **1.1 Was the allocation sequence random?** | **Y** | “*…with a blocking factor of 7, **according to a single,* ***computer-generated allocation schedule***” |
| **1.2 Was allocation concealed until participants were enrolled & assigned?** | **Y** | “*Patients, investigators, and coordinating-centre staff were* ***blinded to the treatment schedule until*** *all corrections to the database were completed*” |
| **1.3 Did baseline differences suggest a problem?** | **N** | “*Baseline characteristics were* ***similar among the treatment groups***” . Table 1 shows closely matched demographics and lung-function values |

**Domain 1 judgment: Low risk**
*Concealment (1.2 = Y) and random sequence (1.1 = Y) in place; baseline balanced (1.3 = N) → Low risk.*

**DOMAIN 2 – Risk of bias due to deviations from intended interventions**

| **Signalling question** | **Answer** | **Direct-quote justification** |
| --- | --- | --- |
| **2.1 Were participants aware of their assignment?** | **N** | “*Randomized,* ***double-blind, double-dummy****, placebo-controlled…*” |
| **2.2 Were carers/personnel aware?** | **N** | Same statement as 2.1 |
| *Because 2.1 = 2.2 = N → Part 1 = Low risk (branching logic).* |  |  |
| **2.6 Was an appropriate analysis used (effect of assignment)?** | **PN** | “*The analysis …* ***included all patients who had a measurement taken at baseline and at least one measurement taken after randomisation***” . Fifteen protocol-violation cases plus 10 without FEV₁ data were excluded before unblinding . |
| **2.7 Substantial bias from the analysis approach?** | **PN** | Exclusions affected < 3 % of 895 randomised patients, evenly distributed, making substantial bias unlikely. |

**Domain 2 judgment: Some concerns**
*Blinding adequate (Part 1 = Low risk). Modified ITT excluded a small proportion of randomised participants (2.6 = PN); this yields Part 2 = Some concerns → overall “Some concerns”.*

**DOMAIN 3 – Risk of bias due to missing outcome data**

| **Signalling question** | **Answer** | **Direct-quote justification** |
| --- | --- | --- |
| **3.1 Were outcome data available for nearly all?** | **Y** | Only “*10 patients lacked a baseline or a post-allocation FEV₁ measurement*” out of 895 randomised (≈ 1 %) |

**Domain 3 judgment: Low risk**
*> 95 % of randomised participants contributed FEV₁ data; attrition unlikely to bias results.*

**DOMAIN 4 – Risk of bias in measurement of the outcome**

| **Signalling question** | **Answer** | **Direct-quote justification** |
| --- | --- | --- |
| **4.1 Was the outcome measurement method inappropriate?** | **N** | “*Centralised spirometry training … according to* ***American Thoracic Society criteria*** *… data were transmitted electronically to a central database and monitored continuously for quality*” |
| **4.2 Could measurement differ between groups?** | **N** | Same standardised, blinded procedures and central QC for all arms |

**Domain 4 judgment: Low risk**
*Objective FEV₁ measured identically across groups with rigorous QC.*

**DOMAIN 5 – Risk of bias in selection of the reported result**

| **Signalling question** | **Answer** | **Direct-quote justification** |
| --- | --- | --- |
| **5.1 Data analysed per pre-specified plan?** | **PY** | Authors state “*primary objective was to compare montelukast with placebo with respect to* ***two prespecified primary endpoints: FEV₁ and daytime symptom score***” |
| **5.2 Were multiple eligible outcome measurements selected?** | **N** | Only the prespecified FEV₁ metric (percentage change) is reported; no alternative metrics presented. |
| **5.3 Were multiple eligible analyses selected?** | **N** | Single pre-stated ANOVA model described; no evidence of selective analytical choices |

**Domain 5 judgment: Low risk**
*Prespecified outcome/analysis reported; no signs of selective reporting.*

**Overall risk-of-bias judgment: Some concerns**

*Domains 1, 3, 4, and 5 = Low risk; Domain 2 = Some concerns due to the modified ITT analysis that excluded a small number of randomised participants.*

## Study 2

Below is a structured RoB 2 assessment for the RCT **“Risedronate Increases Bone Mass in an Early Postmenopausal Population: Two Years of Treatment Plus One Year of Follow-Up”**.
The primary outcome assessed is **percentage change in lumbar-spine BMD at 24 months** (the trial’s declared primary endpoint).

**DOMAIN 1 – Bias arising from the randomisation process**

| **Signalling question** | **Code** | **Reasoning** |
| --- | --- | --- |
| **1.1 Random sequence generated?** | **PY** | Trial states participants were “*randomly assigned*” after stratification by calcium intake; no further detail on sequence generation is given. |
| **1.2 Allocation concealed?** | **PN** | The article gives **no description** of any mechanism (e.g., central allocation, opaque envelopes) used to conceal the sequence before enrolment. |
| **1.3 Baseline imbalances?** | **N** | Authors report “*Baseline characteristics … were similar among treatment groups*.” |

**Domain-level judgment: High risk**
**Justification:** 1.2 = PN ⇒ per RoB 2 algorithm Domain 1 = High risk, regardless of 1.1/1.3.

**DOMAIN 2 – Bias due to deviations from intended interventions**

(effect of *assignment* to intervention)

| **Signalling question** | **Code** | **Reasoning** |
| --- | --- | --- |
| **2.1 Participants aware?** | **N** | “*This double-blind, placebo-controlled study…*” |
| **2.2 Carers/personnel aware?** | **N** | “*The blind regarding treatment assignment was maintained throughout the study.*” |
| **2.3 Deviations due to awareness?** | – | Not reached (2.1 & 2.2 = N) |
| **2.6 Appropriate analysis (ITT)?** | **Y** | Primary analysis used an *intent-to-treat* population including all randomised patients with available data. |

**Domain-level judgment: Low risk**
**Justification:** Part 1 = Low (blinding intact, no relevant deviations); Part 2 = Low (ITT used) → Domain 2 Low risk.

**DOMAIN 3 – Bias due to missing outcome data**

| **Signalling question** | **Code** | **Reasoning** |
| --- | --- | --- |
| **3.1 Nearly all data available?** | **N** | Only 62/111 (≈56 %) participants provided BMD at 24 months. |
| **3.2 Evidence that results not biased?** | **N** | No sensitivity analyses or explanations addressing impact of this attrition. |
| **3.3 Could missingness depend on true value?** | **PY** | Many withdrawals occurred after Year 1 because the trial was initially 1 yr; poorer responders might preferentially discontinue. |
| **3.4 Is this likely?** | **PY** | High attrition with no analysis suggests a plausible, possibly differential impact on outcomes. |

**Domain-level judgment: High risk**
**Justification:** 3.1 = N; 3.2 = N; 3.3 = PY; 3.4 = PY ⇒ algorithm yields High risk.

**DOMAIN 4 – Bias in measurement of the outcome**

| **Signalling question** | **Code** | **Reasoning** |
| --- | --- | --- |
| **4.1 Outcome measurement inappropriate?** | **N** | BMD measured with validated dual-energy X-ray absorptiometry (Hologic QDR 1000) and cross-calibrated scanners. |
| **4.2 Measurement differ between groups?** | **N** | Same DXA devices/protocols used across centres; fractured vertebrae excluded uniformly. |
| **4.3 Assessors aware of intervention?** | **N** | Study was double-blind; DXA technicians therefore blinded. |
| *Further questions not triggered* |  |  |

**Domain-level judgment: Low risk**
**Justification:** 4.1 = N, 4.2 = N, 4.3 = N ⇒ Low risk.

**DOMAIN 5 – Bias in selection of the reported result**

| **Signalling question** | **Code** | **Reasoning** |
| --- | --- | --- |
| **5.1 Analysis pre-specified?** | **NI** | No protocol or dated SAP is referenced. |
| **5.2 Result selected from multiple measurements?** | **PN** | Although multiple skeletal sites were measured, the lumbar-spine 24-month endpoint was clearly highlighted as *the* primary outcome in Methods; most time-points/sites are reported. |
| **5.3 Result selected from multiple analyses?** | **PN** | Only a straightforward ITT analysis is presented; no evidence of undisclosed alternative analyses. |

**Domain-level judgment: Some concerns**
**Justification:** Neither 5.2 nor 5.3 reached Y/PY, but absence of a pre-specified protocol (5.1 = NI) leaves *some concerns*.

**OVERALL RISK-OF-BIAS JUDGMENT**

**Overall judgment: High risk of bias**
**Summary:** Domains 1 and 3 are both at High risk (allocation concealment not reported; substantial, unaddressed attrition). Even with Low risk in Domains 2 & 4 and only Some concerns in Domain 5, the trial as a whole is judged High risk.

## Study 3

Below is a complete **RoB 2 (2019) assessment** for the outcome **“Insulin area-under-the-curve (AUC) from the oral-glucose-tolerance test”** in *Exercise Dose and Diabetes Risk in Overweight and Obese Children* (JAMA 2012).

**DOMAIN 1 – Bias arising from the randomization process**

| **Signalling question** | **Code** | **Why I chose this code** |
| --- | --- | --- |
| **1.1 Was the allocation sequence random?** | **Y** | “Each participant was assigned a uniform (0, 1) random number using SAS… within their respective race and sex group” |
| **1.2 Was the sequence concealed until enrolment & assignment?** | **Y** | “Assignments were **concealed until baseline testing was completed**, then communicated to the study coordinator” |
| **1.3 Did baseline differences suggest a problem?** | **N** | Table 1 shows no significant between-group differences; authors state “There were **no significant group differences (P > .05)**” |

**Domain 1 judgment:** **Low risk**
**Justification:** Concealed, computer-generated randomization (1.1 = Y, 1.2 = Y) with balanced baseline characteristics (1.3 = N) → Low risk.

**DOMAIN 2 – Bias due to deviations from intended interventions (effect of assignment)**

| **Signalling question** | **Code** | **Why I chose this code** |
| --- | --- | --- |
| **2.1 Were participants aware of their assignment?** | **Y** | Study states “**participants were not blinded** to condition” |
| **2.2 Were carers/personnel aware?** | **Y** | Same sentence notes **measurement staff were not blinded** |
| **2.3 Were there deviations arising because of this awareness?** | **PN** | Exceptional adherence (85 % attendance) and identical co-interventions reported; authors note “the trial had **exceptional adherence and retention**” |
| **2.6 Was an appropriate (ITT) analysis used?** | **Y** | Outcomes were “**analyzed by intention to treat**” using mixed models |

*(Questions 2.4, 2.5, 2.7 not triggered.)*

**Domain 2 judgment:** **Low risk**
**Justification:** Although unblinded (2.1/2.2 = Y), no meaningful protocol deviations were evident (2.3 = PN) and ITT analysis was applied (2.6 = Y). Part 1 = Low risk; Part 2 = Low risk → Domain 2 = Low risk.

**DOMAIN 3 – Bias due to missing outcome data**

| **Signalling question** | **Code** | **Why I chose this code** |
| --- | --- | --- |
| **3.1 Were outcome data available for nearly all participants?** | **N** | 94 % retention (209/222) at post-test for primary outcomes . Twenty-nine children lacked baseline OGTT data for insulin AUC |
| **3.2 Evidence that results were not biased by missing data?** | **PN** | Mixed-model maximum-likelihood handles missingness, but no sensitivity analyses reported |
| **3.3 Could missingness depend on true outcome?** | **PN** | Drop-outs were few and reasons (e.g., refused test/blood draw) are unlikely linked strongly to insulin resistance; no group imbalance |

*(3.4 not triggered.)*

**Domain 3 judgment:** **Low risk**
**Justification:** Slightly <95 % data (3.1 = N) but no differential missingness and plausible MAR handling (3.2 = PN, 3.3 = PN) → Low risk.

**DOMAIN 4 – Bias in measurement of the outcome**

| **Signalling question** | **Code** | **Why I chose this code** |
| --- | --- | --- |
| **4.1 Was the measurement method inappropriate?** | **N** | Insulin measured by **radio-immunoassay** with CV ≤ 4.5 % and AUC calculated from timed OGTT samples |
| **4.2 Could measurement differ between groups?** | **N** | Same OGTT protocol and central lab for all groups |
| **4.3 Were assessors aware of assignment?** | **Y** | Measurement staff unblinded |
| **4.4 Could awareness influence assessment?** | **N** | Outcome is an **objective biochemical assay**; assessor knowledge cannot alter machine-read insulin concentrations |

**Domain 4 judgment:** **Low risk**
**Justification:** Objective laboratory outcome with standard methods (4.1/4.2 = N). Although assessors were aware (4.3 = Y), their awareness cannot influence an automated assay (4.4 = N) → Low risk.

**DOMAIN 5 – Bias in selection of the reported result**

| **Signalling question** | **Code** | **Why I chose this code** |
| --- | --- | --- |
| **5.1 Was the analysis pre-specified?** | **PY** | Trial registered (NCT00108901) and primary outcomes/analysis plan described, but protocol not published alongside |
| **5.2 Result selected from multiple measurements?** | **PN** | Single OGTT-based insulin AUC specified; no alternative time-points/scales reported |
| **5.3 Result selected from multiple analyses?** | **PN** | Standard ITT mixed model used; subgroup and per-protocol results also presented, suggesting transparency |

**Domain 5 judgment:** **Low risk**
**Justification:** With 5.2 & 5.3 both PN and 5.1 = PY, algorithm → Low risk.

**Overall risk-of-bias judgment: Low risk**

All five domains were judged **Low risk**; therefore, the overall risk of bias for the insulin AUC outcome is **Low**.

## Study 4

**RCT assessed:** García-Morales JM et al. “Effect of a Dynamic Exercise Program in Combination With Mediterranean Diet on Quality of Life in Women With Rheumatoid Arthritis.” J Clin Rheumatol 2019. Primary outcome: change in global SF-36v2 score at 24 weeks.

**DOMAIN 1 — Risk of Bias arising from the Randomization Process**

| **Signalling question** | **Code** | **Direct-quote–based reasoning** |
| --- | --- | --- |
| **1.1 Was the allocation sequence random?** | **Y** | “*Subjects were randomly assigned, using a randomized sequence created on the website www.randomization.com*” |
| **1.2 Was the allocation sequence concealed until participants were enrolled and assigned?** | **PY** | “*An external collaborator managed the random allocation sequence, and the investigators performed the enrollment process*” – suggests concealment, but no details of the method (e.g., central system, opaque envelopes). |
| **1.3 Did baseline differences suggest a problem with the randomization process?** | **PY** | Table 1 shows significant imbalances: *“Baseline physical function p = 0.001; baseline HAQ-DI p = 0.001.”* |

**Domain 1 Judgment: Some concerns**
*Allocation sequence was random (1.1 = Y) and probably concealed (1.2 = PY), but significant baseline imbalances (1.3 = PY) → Some concerns.*

**DOMAIN 2 — Risk of Bias due to Deviations from Intended Interventions (effect of assignment)**

| **Signalling question** | **Code** | **Direct-quote–based reasoning** |
| --- | --- | --- |
| **2.1 Were participants aware of their assigned intervention?** | **Y** | Open-label lifestyle trial; exercise classes and diet sessions were delivered face-to-face. |
| **2.2 Were carers/personnel aware of assignments?** | **Y** | “*A trained physiotherapist supervised all patients who performed the DEP*.” |
| **2.3 Were there deviations from intended intervention due to the trial context?** | **PN** | Adherence monitored (“*Exercise adherence was defined if patients assisted at least 80 % of sessions*”); no cross-overs or co-interventions reported. |
| *2.4–2.5 not triggered (2.3 = PN)* |  |  |
| **2.6 Was an appropriate analysis used to estimate the effect of assignment?** | **N** | Outcomes analysed for the 130 completers only (“*144 women … 130 completed the follow-up*”); no ITT stated. |
| **2.7 Potential for substantial bias due to inappropriate analysis?** | **PN** | 10 % attrition; imbalance not reported—bias possible but probably small. |

**Domain 2 Judgment: Some concerns**
*Part 1 = Low risk (open-label but no material deviations); Part 2 = Some concerns (non-ITT analysis) → Some concerns.*

**DOMAIN 3 — Risk of Bias due to Missing Outcome Data**

| **Signalling question** | **Code** | **Direct-quote–based reasoning** |
| --- | --- | --- |
| **3.1 Were outcome data available for nearly all participants?** | **N** | 130/144 (90.3 %) provided SF-36 data. |
| **3.2 Evidence that results were not biased by missing data?** | **N** | No sensitivity analyses or imputation described. |
| **3.3 Could missingness depend on true value of the outcome?** | **PY** | Dropouts may relate to poor adherence or outcomes; no reasons given. |
| **3.4 Is it likely that it did?** | **PN** | Attrition modest and evenly distributed; substantial bias unlikely but cannot be ruled out. |

**Domain 3 Judgment: Some concerns**
*Incomplete outcome data with no sensitivity checks and some possibility outcome-related → Some concerns.*

**DOMAIN 4 — Risk of Bias in Measurement of the Outcome**

| **Signalling question** | **Code** | **Direct-quote–based reasoning** |
| --- | --- | --- |
| **4.1 Was the method of measuring the outcome inappropriate?** | **N** | “*Health-related quality of life was assessed with 36-item Short Form Health Survey v2*” (validated). |
| **4.2 Could measurement differ between groups?** | **N** | Same questionnaire and time-points for all groups. |
| **4.3 Were outcome assessors aware of intervention?** | **Y** | Participants (self-assessors) necessarily knew their allocation in this open-label design. |
| **4.4 Could awareness influence assessment?** | **Y** | HRQoL is subjective; expectations could alter responses. |
| **4.5 Was assessment likely influenced?** | **PY** | Influence plausible given subjective measure and lack of blinding. |

**Domain 4 Judgment: High risk**
*Unblinded participants self-reported a subjective primary outcome (4.3 = Y, 4.4 = Y, 4.5 = PY) → High risk.*

**DOMAIN 5 — Risk of Bias in Selection of the Reported Result**

| **Signalling question** | **Code** | **Direct-quote–based reasoning** |
| --- | --- | --- |
| **5.2 Result selected from multiple outcome measurements?** | **PN** | Only baseline and 24-week HRQoL reported; no unused alternative time points/scales. |
| **5.3 Result selected from multiple eligible analyses?** | **PN** | Single ANCOVA/within-group change analysis stated; no alternative models described. |
| **5.1 Analysis pre-specified before unblinded data access?** | **PY** | Trial registered on ClinicalTrials.gov (NCT02900898) but SAP not published. |

**Domain 5 Judgment: Low risk**
*No evidence of selective measurement/analysis (5.2 = PN, 5.3 = PN) and prospective registration (5.1 = PY) → Low risk.*

**OVERALL Risk-of-Bias Judgment: High risk**

*Domain 4 is High risk due to unblinded participant-reported subjective outcome; Domains 1–3 show Some concerns; Domain 5 is Low risk → Overall High risk of bias for the primary outcome.*

## Study 5

Below is a completed RoB 2.0 assessment for **Arntz A. (2003) “Cognitive therapy versus applied relaxation as treatment of generalized anxiety disorder.”**
Primary outcome assessed = **Self-reported anxiety on the SCL-90 Anxiety sub-scale** (identified by the authors as “the most direct index of general anxiety complaints” ).

**DOMAIN 1 – Risk of bias arising from the randomization process**

| **Signalling question** | **Answer** | **Direct quote / justification** |
| --- | --- | --- |
| **1.1 Was the allocation sequence random?** | PN | “*They were randomly allocated to condition and to the next available therapist.*”  No method for sequence generation is described. |
| **1.2 Was the allocation sequence concealed until participants were enrolled and assigned?** | NI | No statement anywhere in Methods or Procedure about concealment (central randomization, opaque envelopes, etc.). |
| **1.3 Did baseline differences suggest a problem with the randomization process?** | Y | “*Two significant differences were found: 4 of 25 CT subjects had no partner vs 9 of 20 AR … educational level was lower in the AR condition*” |

**Domain 1 judgment:** **High risk**
**Justification:** 1.2 = NI → must examine 1.3; significant baseline imbalances (1.3 = Y) elevate the domain to High risk per RoB 2 algorithm.

**DOMAIN 2 – Risk of bias due to deviations from intended interventions (effect of assignment)**

**Part 1 – Deviations**

| **Q** | **A** | **Quote / rationale** |
| --- | --- | --- |
| **2.1 Participants aware?** | Y | Psychotherapeutic trial; no blinding described; interventions delivered face-to-face over 12 sessions. |
| **2.2 Carers/personnel aware?** | Y | “*Thirteen therapists conducted both treatments.*” |
| **2.3 Deviations due to awareness?** | PN | No evidence of participants switching arms or receiving ancillary co-interventions attributable to awareness; drop-outs occurred but not linked to knowing allocation. |
| **2.4–2.5** | — | Not triggered (2.3 = PN). |

**Part 1 judgment:** Low risk (open-label, but no meaningful deviations).

**Part 2 – Analysis**

| **Q** | **A** | **Quote / rationale** |
| --- | --- | --- |
| **2.6 Appropriate analysis for effect of assignment?** | Y | “*An intention-to-treat analysis was carried out… missing values were estimated by inter/ extrapolation.*” |
| **2.7** | — | Not triggered (2.6 = Y). |

**Part 2 judgment:** Low risk.

**Domain 2 judgment:** **Low risk**
**Justification:** Both Part 1 and Part 2 were Low risk.

**DOMAIN 3 – Risk of bias due to missing outcome data**

| **Q** | **A** | **Quote / rationale** |
| --- | --- | --- |
| **3.1 Outcome data for nearly all?** | N | 8 / 45 (17.8 %) participants dropped out; two supplied follow-up data, leaving >5 % missing . |
| **3.2 Evidence results not biased?** | N | No sensitivity analysis beyond simple interpolation; no justification that missingness is ignorable. |
| **3.3 Could missingness depend on true outcome?** | Y | Reasons include dissatisfaction with treatment and worsening depression —plausibly related to poor outcomes. |
| **3.4 Is it likely?** | Y | Drop-outs driven by lack of improvement or adverse mental-health events; likely linked to higher anxiety. |

**Domain 3 judgment:** **High risk**
**Justification:** 3.1 = N; 3.2 = N; 3.3 = Y; 3.4 = Y ⇒ High risk.

**DOMAIN 4 – Risk of bias in measurement of the outcome**

| **Q** | **A** | **Quote / rationale** |
| --- | --- | --- |
| **4.1 Inappropriate measurement method?** | N | Validated scales used: “*SCL-90… alpha of anxiety scale = 0.88*” |
| **4.2 Could measurement differ between groups?** | N | Same self-report instruments given to both groups at identical time-points. |
| **4.3 Were outcome assessors aware?** | Y | Outcomes are participant self-reports; participants were unblinded. |
| **4.4 Could awareness influence assessment?** | Y | Anxiety self-ratings are subjective and easily influenced by expectations. |
| **4.5 Was it likely?** | Y | Open-label psychotherapy with expectancy effects; no blinding mitigations described. |

**Domain 4 judgment:** **High risk**
**Justification:** Unblinded, subjective self-report outcomes with high potential for bias (4.3–4.5 = Y).

**DOMAIN 5 – Risk of bias in selection of the reported result**

| **Q** | **A** | **Quote / rationale** |
| --- | --- | --- |
| **5.2 Result selected from multiple outcome measurements?** | PY | Authors analysed several scales and time-points but did not state a priori which was primary: “*Two measures were also separately analyzed because they were judged to be especially relevant…*” |
| **5.3 Result selected from multiple eligible analyses?** | PY | Both completer and ITT analyses reported; unclear whether other models were examined and omitted. |

*(5.1 not evaluated because 5.2/5.3 already raise concerns.)*

**Domain 5 judgment:** **High risk**
**Justification:** Presence of multiple possible measurements/analyses without prespecification (5.2 = PY, 5.3 = PY) → High risk per algorithm.

**OVERALL RISK-OF-BIAS JUDGMENT: High risk**

**Summary:** Domains 1, 3, 4 and 5 each carry High risk assessments, driven by baseline imbalances, attrition linked to outcome, unblinded subjective outcome measurement, and un-prespecified multiplicity of analyses.

## Study 6

**PRIMARY OUTCOME chosen for this RoB-2 assessment**
Maximum knee-extension (KE) power at 12 weeks.

**DOMAIN 1 – Bias arising from the randomisation process**

| **Question** | **Answer** | **Reasoning (direct quotes)** |
| --- | --- | --- |
| **1.1 Was the allocation sequence random?** | **Y** | “participants were assigned to treatment using a **computer-generated randomization scheme** integrated into a Web-based data-entry and -management system” |
| **1.2 Was the allocation sequence concealed until participants were enrolled and assigned?** | **Y** | Same Web-based central system implies allocation was concealed before assignment |
| **1.3 Did baseline differences suggest a problem with the randomisation process?** | **N** | “**There were no baseline differences** in any of the sociodemographic, physical-function, and health-status variables between the three groups” |

**Domain 1 judgment:** **Low risk**
**Justification:** Allocation was random (1.1 = Y) and centrally concealed (1.2 = Y); baseline covariates balanced (1.3 = N) → Low risk.

**DOMAIN 2 – Bias due to deviations from intended interventions (effect of assignment)**

| **Question** | **Answer** | **Reasoning** |
| --- | --- | --- |
| **2.1 Were participants aware of their assigned intervention?** | **Y** | Participants trained in separate PT or ST sessions; no blinding described |
| **2.2 Were carers/personnel aware?** | **Y** | “sessions were **supervised by… certified interventionists**” who delivered the specific protocol |
| **2.3 Deviations from intended intervention due to trial context?** | **N** | Interventionists “made sure that participants… completed the resistance exercises with **correct form**” with no report of cross-over or additional treatments |
| **2.6 Appropriate analysis for effect of assignment?** | **Y** | “**All follow-up data on participants were used** in the analyses” via repeated-measures ANCOVA |

*Branching logic:* 2.1/2.2 = Y → check 2.3 (N) → Part 1 = Low risk; 2.6 = Y → Part 2 = Low risk → **Domain 2 = Low risk**

**DOMAIN 3 – Bias due to missing outcome data**

| **Question** | **Answer** | **Reasoning** |
| --- | --- | --- |
| **3.1 Were outcome data available for nearly all participants?** | **N** | 12/15 PT, 11/15 ST, 13/15 control completed primary testing (≤ 87%) |
| **3.2 Evidence that results were not biased by missing data?** | **NI** | No sensitivity or imputation methods reported. |
| **3.3 Could missingness depend on true outcome?** | **NI** | Drop-outs due to medical events/time conflicts; unclear relation to muscle power |
| **3.4 If Y/PY/NI to 3.3: Is it likely?** | **PN** | Reasons given (time conflicts) unlikely to be strongly outcome-related. |

**Domain 3 judgment:** **Some concerns**
**Justification:** < 95 % outcome data (3.1 = N) with no analysis to address missingness (3.2 = NI); uncertainty about mechanism of missingness gives Some concerns.

**DOMAIN 4 – Bias in measurement of the outcome**

| **Question** | **Answer** | **Reasoning** |
| --- | --- | --- |
| **4.1 Was the method of measuring the outcome inappropriate?** | **N** | KE power measured on calibrated Keiser pneumatic machines; objective, standardised |
| **4.2 Could measurement differ between groups?** | **N** | Same machines and protocol used for all groups |
| **4.3 Were outcome assessors aware of intervention?** | **PY** | Blinding not reported; staff likely aware, but outcome objectively recorded. |
| **4.4 Could awareness influence assessment?** | **PN** | Power output is machine-read, reducing potential influence. |

*Branching logic:* 4.1 = N; 4.2 = N → assess 4.3; 4.3 = PY then 4.4 = PN → **Domain 4 = Low risk**

**DOMAIN 5 – Bias in selection of the reported result**

| **Question** | **Answer** | **Reasoning** |
| --- | --- | --- |
| **5.1 Was the analysis plan pre-specified?** | **NI** | No protocol or SAP referenced. |
| **5.2 Result selected from multiple outcome measurements?** | **PN** | Strength & power outcomes reported consistently; little scope for alternative measures. |
| **5.3 Result selected from multiple eligible analyses?** | **PN** | Single primary analysis (repeated-measures ANCOVA) described. |

*Algorithm:* 5.2 & 5.3 = PN (not Y/PY); 5.1 = NI → **Domain 5 = Some concerns**

**OVERALL RISK-OF-BIAS JUDGMENT**

**Overall judgment:** **Some concerns**
**Brief rationale:** Domains 1, 2, 4 = Low risk; Domain 3 and 5 = Some concerns (no High-risk domains) → Overall **Some concerns** for the effect of power vs strength training on KE power at 12 weeks.

## Study 7

**Primary outcome assessed:** Change in Barnes Akathisia Rating Scale (BARS) *global* score

**DOMAIN 1 – Bias arising from the randomization process**

| **Signalling question** | **Answer** | **Reasoning** |
| --- | --- | --- |
| **1.1 Was the allocation sequence random?** | **PN** | The report only states that “*Patients were randomly assigned to either the order Trz-Pla or the order Pla-Trz*” with no details of how the sequence was generated . |
| **1.2 Was the allocation sequence concealed until participants were enrolled?** | **NI** | The article gives no information on any concealment method (e.g., central randomization, sealed envelopes, identical containers). |
| **1.3 Did baseline differences suggest a problem?** | **N** | Authors note that *“At baseline, there were no statistical differences between the groups in all outcome measures (all P > 0.14)”* , and Table 2 shows similar values . |

**Domain 1 Judgment:** **Some concerns**
**Justification:** 1.2 = NI triggers reliance on 1.3; with balanced baselines (1.3 = N) the algorithm yields *Some concerns*.

**DOMAIN 2 – Bias due to deviations from intended interventions (effect of assignment)**

| **Signalling question** | **Answer** | **Reasoning** |
| --- | --- | --- |
| **2.1 Were participants aware of their intervention?** | **N** | The trial is explicitly described as “*double-blind, placebo-controlled*” . |
| **2.2 Were carers/personnel aware?** | **N** | Same double-blind statement applies to staff administering identical capsules . |
| **2.3 – 2.5 (Deviations)** | — | Not reached because 2.1 = 2.2 = N. |
| **2.6 Was an appropriate analysis used?** | **PY** | All 13 randomized participants contributed data to every analysis; no exclusions are reported, but “intention-to-treat” is not explicitly mentioned . |
| **2.7 Potential for bias from analysis?** | — | Not triggered (2.6 = PY). |

**Domain 2 Judgment:** **Low risk**
**Justification:** Blinding was maintained (Part 1 = Low risk) and all participants were analysed (Part 2 = Low risk).

**DOMAIN 3 – Bias due to missing outcome data**

| **Signalling question** | **Answer** | **Reasoning** |
| --- | --- | --- |
| **3.1 Outcome data for nearly all participants?** | **Y** | All 13 randomized patients provided outcome measurements through day 6; no drop-outs are reported . |

*(Steps 3.2–3.4 not triggered.)*

**Domain 3 Judgment:** **Low risk**
**Justification:** Complete outcome data (> 95 %) were available.

**DOMAIN 4 – Bias in measurement of the outcome**

| **Signalling question** | **Answer** | **Reasoning** |
| --- | --- | --- |
| **4.1 Was outcome measurement inappropriate?** | **N** | The validated BARS scale was used to rate akathisia . |
| **4.2 Could measurement differ between groups?** | **N** | Same scale and identical schedule applied in both periods; no indication of differential methods. |
| **4.3 Were outcome assessors aware of assignment?** | **PN** | Assessments were by a single rater (“*All psychometric assessments were performed by a single rater (S.R.)*” ). The trial is described as double-blind, but assessor blinding is not explicitly confirmed. |

(4.4–4.5 not triggered because 4.3 = PN.)

**Domain 4 Judgment:** **Low risk**
**Justification:** Valid, uniform measurement with probable assessor blinding; algorithm gives Low risk.

**DOMAIN 5 – Bias in selection of the reported result**

| **Signalling question** | **Answer** | **Reasoning** |
| --- | --- | --- |
| **5.2 Result selected from multiple measurements?** | **NI** | Only one time-point series (baseline, day 3, day 6) for BARS is reported, but the report does not clarify whether other outcome definitions were possible. |
| **5.3 Result selected from multiple analyses?** | **NI** | No protocol or SAP is cited; it is unclear whether alternate analyses were explored. |
| **5.1 Analysis pre-specified?** | **NI** | No registration or dated SAP is provided. |

**Domain 5 Judgment:** **Some concerns**
**Justification:** With 5.2 = 5.3 = NI the algorithm assigns Some concerns.

**Overall risk-of-bias judgment: Some concerns**

*Domains 1 and 5 carry “Some concerns” while Domains 2–4 are Low risk; no domain is High risk, yielding an overall judgment of Some concerns.*

## Study 8

**DOMAIN 1 – Risk of Bias Arising from the Randomization Process**

| **Signalling question** | **Code** | **Reasoning (direct quote)** |
| --- | --- | --- |
| 1.1 Was the allocation sequence random? | PY | “*The order in which subjects received the 2 different treatments was* ***randomized*** *and known only by the research pharmacist.*” – randomization is stated but the method for generating the sequence is not described. |
| 1.2 Was the allocation sequence concealed until participants were enrolled and assigned? | Y | Same sentence indicates concealment: only the pharmacist knew the order, so investigators and participants were masked. |
| 1.3 Did baseline differences suggest a problem with the randomization process? | N | Because “*subjects acting as their own control*” eliminates between-group baseline differences. |

**Domain-level judgment: Low risk**
*Allocation was concealed (1.2 = Y); sequence plausibly random (1.1 = PY); with a crossover design the same individuals received both treatments (1.3 = N) → Low risk.*

**DOMAIN 2 – Risk of Bias Due to Deviations from Intended Interventions (Effect of Assignment)**

| **Signalling question** | **Code** | **Reasoning (direct quote)** |
| --- | --- | --- |
| 2.1 Were participants aware of their assigned intervention? | PY | “*The drug order was almost always apparent to subjects … whether the treatment was psilocybin or placebo.*” |
| 2.2 Were carers/personnel aware? | PY | Same sentence indicates investigators also recognised the assignment. |
| 2.3 Were there deviations from intended intervention due to the trial context? | N | The report gives no examples of changes in care, cross-over, or non-adherence; each scheduled session was completed as planned. |
| 2.6 Was an appropriate analysis used to estimate the effect of assignment? | Y | “*Raw BDI, POMS, and STAI data were analysed using 2-way ANOVA with drug as the* ***within-subject*** *factor and day as a repeated measure.*” – appropriate for a two-period crossover. |

Part 1 (blinding/deviations) = Low risk (2.3 = N).
Part 2 (analysis) = Low risk (2.6 = Y).

**Domain-level judgment: Low risk**
*Although blinding was imperfect, no protocol deviations occurred and the within-subject analysis was appropriate.*

**DOMAIN 3 – Risk of Bias Due to Missing Outcome Data**

| **Signalling question** | **Code** | **Reasoning (direct quote)** |
| --- | --- | --- |
| 3.1 Were outcome data available for nearly all participants? | N | “*Eight subjects completed the 6-month follow-up… 11 completed at least the first 4 months…*” (for STAI trait anxiety at 3 months 11/12 ≈ 92 %). |
| 3.2 Evidence that results were not biased by missing data? | N | No sensitivity analyses or imputation are reported. |
| 3.3 Could missingness depend on true outcome value? | Y | “*Two subjects died … and 2 others became too ill to continue participating.*” – poorer outcomes likely linked to dropout. |
| 3.4 Is it likely that it did? | Y | Participants with worse health/anxiety plausibly more likely to miss follow-up. |

**Domain-level judgment: High risk**
*<95 % data, no mitigation, and health-related attrition likely related to outcomes.*

**DOMAIN 4 – Risk of Bias in Measurement of the Outcome**

| **Signalling question** | **Code** | **Reasoning (direct quote)** |
| --- | --- | --- |
| 4.1 Was the outcome measurement method inappropriate? | N | STAI trait anxiety is a validated self-report scale. |
| 4.2 Could measurement differ between groups? | N | Both sessions used identical questionnaires and timing. |
| 4.3 Were outcome assessors aware of intervention? | Y | Participants (self-assessors) could usually tell which drug they received. |
| 4.4 Could awareness influence assessment? | Y | Self-reported anxiety is subjective and easily influenced by expectation. |
| 4.5 Is it likely assessment was influenced? | Y | Strong psychoactive effects make bias very likely. |

**Domain-level judgment: High risk**
*Subjective self-ratings by unblinded participants are very likely to be biased.*

**DOMAIN 5 – Risk of Bias in Selection of the Reported Result**

| **Signalling question** | **Code** | **Reasoning (direct quote)** |
| --- | --- | --- |
| 5.2 Result selected from multiple outcome measurements? | PY | Many psychological scales and multiple time-points were collected (BDI, POMS, STAI). |
| 5.3 Result selected from multiple eligible analyses? | PY | Several analyses (within-session, 1 day, 2 weeks, monthly) reported selectively; no protocol available. |

(With PY in either 5.2 or 5.3 the domain is automatically High risk.)

**Domain-level judgment: High risk**

**Overall risk-of-bias judgment: High risk**

*Domains 3, 4, and 5 are High risk, giving an overall High-risk assessment for this crossover RCT.*

## Study 9

**DOMAIN 1: Risk of Bias from the Randomization Process**

| **Signalling question** | **Code** | **Direct-quote justification** |
| --- | --- | --- |
| **1.1** Was the allocation sequence random? | **Y** | “...from a block of drugs that had been pre-packaged for every centre by Pfizer, according to a **computer-generated randomisation code**.” |
| **1.2** Was the allocation sequence concealed until participants were enrolled and assigned? | **Y** | Investigators, pharmacists, study administrators **“were unaware of the randomisation code throughout the study.”** |
| **1.3** Did baseline differences suggest a problem with the randomization process? | **N** | Baseline Table 1 shows closely similar age, sex, lipid values and other characteristics between groups (e.g., age 61.8 vs 61.5 yrs; women 32% vs 32%). |

**Domain 1 Judgment: Low risk**
*Justification*: 1.2 = Y (concealed); 1.1 = Y (computer-generated); proceed to 1.3, which is N (well-balanced) → Low risk.

**DOMAIN 2: Risk of Bias Due to Deviations from Intended Interventions (Effect of Assignment to Intervention)**

| **Signalling question** | **Code** | **Direct-quote justification** |
| --- | --- | --- |
| **2.1** Were participants aware of their assigned intervention? | **N** | “Patients were unaware of the randomisation code throughout the study.” |
| **2.2** Were carers/personnel aware? | **N** | Same statement applies to “investigators, pharmacists, study administrators”. |
| **2.3** Were there deviations from intended intervention due to the trial context? | **N** | No deviations linked to awareness are described. |
| **2.6** Was an appropriate analysis used (effect of assignment)? | **Y** | “**Analysis was by intention to treat.**” ; “All analyses were by intention to treat and were specified in an analysis plan before unmasking.” |

*Part 1* (2.1-2.5): Both 2.1 and 2.2 = N → Low risk.
*Part 2* (2.6-2.7): 2.6 = Y → Low risk.

**Domain 2 Judgment: Low risk**
*Justification*: Blinding of participants and personnel maintained; no awareness-related deviations; ITT analysis used.

**DOMAIN 3: Risk of Bias Due to Missing Outcome Data**

| **Signalling question** | **Code** | **Direct-quote justification** |
| --- | --- | --- |
| **3.1** Were outcome data available for nearly all participants? | **Y** | “2819 (99%) of those randomised were fully assessable for mortality and morbidity at study termination.” |
| (3.2-3.4 not triggered because 3.1 = Y) |  |  |

**Domain 3 Judgment: Low risk**
*Justification*: > 95 % of randomised participants contributed outcome data.

**DOMAIN 4: Risk of Bias in Measurement of the Outcome**

| **Signalling question** | **Code** | **Direct-quote justification** |
| --- | --- | --- |
| **4.1** Was the method of measuring the outcome inappropriate? | **N** | Cardiovascular events had to be “acute and **hospital-verified**.” |
| **4.2** Could measurement differ between groups? | **N** | Same hospital verification and central ECG coding applied to all participants. |
| **4.3** Were outcome assessors aware of intervention? | **NI** | Paper does not explicitly state blinding of endpoint committee. |
| **4.4** Could any awareness influence outcome measurement? | **N** | Outcomes are objective (death, MI, stroke) and not susceptible to assessor judgment. |

Because 4.1 = N and 4.2 = N; even with 4.3 = NI, 4.4 = N (objective outcomes), algorithm → Low risk.

**Domain 4 Judgment: Low risk**
*Justification*: Objective, hospital-verified endpoints make assessor awareness unlikely to bias results.

**DOMAIN 5: Risk of Bias in Selection of the Reported Result**

| **Signalling question** | **Code** | **Direct-quote justification** |
| --- | --- | --- |
| **5.2** Result selected from multiple measurements? | **PN** | Only one definition/time-to-event composite reported for the primary outcome; no alternative measurements indicated. |
| **5.3** Result selected from multiple analyses? | **PN** | Single pre-specified Cox model reported; no evidence of unreported alternative models. |
| **5.1** Was the analysis plan pre-specified before unblinded data access? | **PY** | Analyses “were specified in an analysis plan **before unmasking**.” |

With 5.2 = PN and 5.3 = PN, check 5.1 → PY → Low risk.

**Domain 5 Judgment: Low risk**
*Justification*: Analysis plan existed pre-unblinding; no indication of selective outcome or analysis reporting.

**Overall Risk-of-Bias Judgment: Low risk**

*All five domains were assessed as Low risk; therefore the overall risk of bias for the CARDS trial primary outcome is Low risk.*

## Study 10

**Primary outcome assessed:** Ventilator-free days (VFDs) in the first 28 days after injury

**Domain 1 – Bias arising from the randomization process**

| **Signalling question** | **Response** | **Reasoning** |
| --- | --- | --- |
| 1.1 Was the allocation sequence random? | **Y** | Central, computer-generated sequence via Randomize.net with block size 6 and site stratification |
| 1.2 Was the allocation sequence concealed until participants were enrolled and assigned? | **Y** | Web-based service “concealed to investigators” |
| 1.3 Did baseline differences suggest a problem with randomization? | **N** | Groups were well balanced across all reported characteristics (Table 1) |

**Domain 1 judgment: Low risk** – Sequence was truly random and concealed; baseline covariates balanced.

**Domain 2 – Bias due to deviations from intended interventions (effect of assignment)**

| **Signalling question** | **Response** | **Reasoning** |
| --- | --- | --- |
| 2.1 Were participants aware of their assigned intervention? | **Y** | Trial was open-label; “patients … were not blinded” |
| 2.2 Were carers/personnel aware? | **Y** | Treating physicians also unblinded |
| 2.3 Were there deviations from intended intervention due to the trial context? | **PY** | Six protocol cross-overs (3 per group) shown in CONSORT diagram |
| 2.4 Were these deviations likely to affect the outcome? | **PY** | Switching operative care could plausibly change VFDs. |
| 2.5 Were deviations balanced between groups? | **Y** | Cross-overs were symmetrical (3 vs 3) |
| 2.6 Was an appropriate analysis used (effect of assignment)? | **PY** | Modified ITT excluding post-randomisation ineligibles/withdrawals |
| 2.7 Substantial bias from inappropriate analysis? | — | Not applicable (2.6 = PY ⇒ low concern) |

**Domain 2 judgment: Some concerns** – Open label with minor, balanced cross-overs; ITT largely preserved.

**Domain 3 – Bias due to missing outcome data**

| **Signalling question** | **Response** | **Reasoning** |
| --- | --- | --- |
| 3.1 Were outcome data available for nearly all participants? | **Y** | “The primary outcome was available for all patients, and there were no missing values.” |

**Domain 3 judgment: Low risk** – No missing VFD data.

**Domain 4 – Bias in measurement of the outcome**

| **Signalling question** | **Response** | **Reasoning** |
| --- | --- | --- |
| 4.1 Was the method of measuring the outcome inappropriate? | **N** | VFDs derive from objective ventilator logs. |
| 4.2 Could measurement differ between groups? | **N** | Same ICU records for both groups. |
| 4.3 Were outcome assessors aware of intervention? | **Y** | Investigators “were unable to blind assessors” |
| 4.4 Could awareness have influenced outcome assessment? | **PY** | Extubation/weaning decisions include clinical judgment. |
| 4.5 Is it likely that assessment was influenced? | **PN** | Objective ventilator data plus a standardized weaning protocol reduce risk |

**Domain 4 judgment: Some concerns** – Unblinded assessors could influence timing of extubation, but outcome largely objective.

**Domain 5 – Bias in selection of the reported result**

| **Signalling question** | **Response** | **Reasoning** |
| --- | --- | --- |
| 5.2 Result likely selected from multiple measurements? | **N** | Single definition/time-frame for VFDs reported. |
| 5.3 Result likely selected from multiple analyses? | **PN** | Only prespecified main and subgroup analyses described. |
| 5.1 Was the analysis plan pre-specified before unblinded data access? | **Y** | Protocol and SAP dated in Supplements 1–7 |

**Domain 5 judgment: Low risk** – Pre-registered plan followed; no evidence of selective reporting.

**Overall Risk-of-Bias Judgment: Some concerns**

Allocation was robust and data complete, but the open-label design with unavoidable cross-over plus unblinded outcome assessment introduces some residual uncertainty (Domains 2 & 4).

## Study 11

**DOMAIN 1: Risk of Bias from Randomization Process**
1.1 Allocation sequence random? **PY**
 Reasoning: “Randomization was accomplished prospectively **using block allocation** to ensure equal numbers in each group.” (block randomisation stated but generation method not fully described).

1.2 Allocation sequence concealed? **Y**
 Reasoning: “The random allocation was **concealed using sealed individual numbered envelopes sequestered from the assessors** in the study.”

1.3 Baseline differences? **N**
 Reasoning: Table 2 shows no significant differences in age, height, weight, pain or tenderness (all p ≥ 0.09).

**Domain 1 Judgment: Low risk**
Justification: Concealment clearly described (1.2 = Y) and sequence probably random (1.1 = PY); baseline covariates balanced (1.3 = N) → Low risk.

**DOMAIN 2: Risk of Bias due to Deviations from Intended Interventions (effect of assignment)**
2.1 Participants aware of assignment? **PN**
 Reasoning: Trial described as **“single-blind”** and only ~50 % correctly identified their group at de-briefing ➔ blinding largely held but not perfect.

2.2 Carers/personnel aware? **Y**
 Reasoning: The single treating chiropractor delivered all procedures and necessarily knew which was real or sham. “Each group was given treatment by a practising chiropractor… responsible for carrying out all interventions.”

2.3 Any deviations arising from that awareness? **N**
 Reasoning: Interventions were protocol-fixed (RM + SM vs SM + SM) with no cross-overs or extra care reported.

*(Parts 2.4–2.5 not triggered)*

2.6 Appropriate analysis of assignment? **PN**
 Reasoning: Analysis used only the 64/67 participants with usable data (“Data from 64 subjects… were available for analysis”) with no explicit ITT statement.

2.7 Substantial bias from analysis set? **PN**
 Reasoning: Loss of three participants (≈ 4 %) is small and unlikely to materially bias results; no evidence of selective exclusion.

**Domain 2 Judgment: Some concerns**
Justification: Part 1 Low risk (no harmful deviations), but analysis not clearly ITT (2.6 = PN) → Some concerns.

**DOMAIN 3: Risk of Bias Due to Missing Outcome Data**
3.1 Outcome data for nearly all? **Y**
 Reasoning: 64/67 randomised participants (95.5 %) contributed outcome data.

*(3.2–3.4 not triggered)*

**Domain 3 Judgment: Low risk**
Justification: >95 % data available; attrition minimal.

**DOMAIN 4: Risk of Bias in Measurement of the Outcome** (primary outcome = subject “registration” of group assignment)
4.1 Outcome measurement method inappropriate? **N**
 Reasoning: Participants were directly asked to identify their treatment immediately after procedures—a suitable and straightforward measure of blinding success.

4.2 Could measurement differ between groups? **N**
 Reasoning: Same question administered to both groups under identical conditions.

4.3 Outcome assessors aware of intervention? **N**
 Reasoning: The assessor was the participant themselves, who had been blinded (see 2.1).

*(4.4–4.5 not triggered)*

**Domain 4 Judgment: Low risk**
Justification: Appropriate, identical measurement across groups with blinded respondents.

**DOMAIN 5: Risk of Bias in Selection of the Reported Result**
5.2 Result selected from multiple measurements? **N**
 Reasoning: Only one primary outcome (self-reported group allocation) and one timing (immediately post-procedure) described.

5.3 Result selected from multiple analyses? **PN**
 Reasoning: Statistical approach (difference in proportions χ² test) is standard, but no protocol to confirm pre-specification.

5.1 Analysis plan pre-specified before unblinding? **NI**
 Reasoning: No protocol or SAP referenced.

**Domain 5 Judgment: Some concerns**
Justification: Although outcome choice is singular, absence of a documented pre-specified analysis plan yields some concerns.

**Overall Risk-of-Bias Judgment: Some concerns**

Domains 1, 3 and 4 are Low risk; Domains 2 and 5 present Some concerns (no High-risk domain).

## Study 12

Below is a complete RoB 2 assessment for **the primary outcome “active shoulder-flexion range-of-motion (degrees) measured immediately after the mobilisation session.”**

**DOMAIN 1 – Bias arising from the randomisation process**

| **Signalling question** | **Code** | **Reasoning** |
| --- | --- | --- |
| 1.1 Was the allocation sequence random? | **Y** | “Frozen shoulder patients were divided into two groups **using random number tables** to determine the intervention order.” |
| 1.2 Was the allocation sequence concealed until participants were enrolled and assigned? | **NI** | The paper gives **no information** on any concealment method (e.g., opaque envelopes, central randomisation). |
| 1.3 Did baseline differences suggest a problem with the randomisation process? | **N** | “There were **no differences** in the demographic characteristics among the groups (p > 0.05, Table 1).” |

**Domain 1 judgment: Some concerns**
*Allocation was clearly random (1.1 = Y) but concealment cannot be verified (1.2 = NI); with balanced baselines (1.3 = N) this yields “Some concerns” under the algorithm.*

**DOMAIN 2 – Bias due to deviations from intended interventions (effect of assignment)**

| **Signalling question** | **Code** | **Reasoning** |
| --- | --- | --- |
| 2.1 Were participants aware of their assigned intervention? | **Y** | The trial is described as **“single-blinded”**—only the assessor was blinded, so participants were aware. |
| 2.2 Were carers/personnel aware of the intervention? | **Y** | The treating physiotherapist delivered the specific manual techniques; no blinding is reported. |
| 2.3 Were there deviations from intended intervention due to the trial context? | **PN** | Each technique was delivered once under direct supervision; the paper reports no cross-overs, extra care, or non-adherence. |
| 2.4–2.5 (if reached) | — | Not applicable – 2.3 = PN. |
| 2.6 Was an appropriate analysis used to estimate the effect of assignment? | **Y** | All 54 randomised patients were analysed; pre–post change tested with paired t-tests and ANOVA, with **no exclusions**. |
| 2.7 (if reached) | — | Not applicable – 2.6 = Y. |

**Domain 2 judgment: Low risk**
*Although participants and carers were unblinded, no deviations linked to that awareness were reported (Part 1 = Low risk) and all randomised patients were analysed (Part 2 = Low risk).*

**DOMAIN 3 – Bias due to missing outcome data**

| **Signalling question** | **Code** | **Reasoning** |
| --- | --- | --- |
| 3.1 Were outcome data available for nearly all participants? | **Y** | The results tables present data for **all 54 participants pre- and post-intervention**; no drop-outs are mentioned. |

(**Questions 3.2–3.4 not triggered**)

**Domain 3 judgment: Low risk**

**DOMAIN 4 – Bias in measurement of the outcome**

| **Signalling question** | **Code** | **Reasoning** |
| --- | --- | --- |
| 4.1 Was the method of measuring the outcome inappropriate? | **N** | Shoulder ROM was measured with a **goniometer**, a standard validated tool (ref 17 cited). |
| 4.2 Could measurement differ between groups? | **N** | The same assessor, positions, and instruments were used for every participant. |
| 4.3 Were outcome assessors aware of intervention? | **N** | “Assessments were performed … **by a blinded assessor**.” |

(**Questions 4.4–4.5 not triggered**)

**Domain 4 judgment: Low risk**

**DOMAIN 5 – Bias in selection of the reported result**

| **Signalling question** | **Code** | **Reasoning** |
| --- | --- | --- |
| 5.1 Was the analysis plan pre-specified? | **NI** | No protocol or SAP is referenced. |
| 5.2 Is the result likely selected from multiple outcome measurements? | **PN** | For flexion ROM the paper specifies **one time-point only (pre vs. immediate post)**, matching the methods. |
| 5.3 Is the result likely selected from multiple eligible analyses? | **PN** | A single, straightforward analysis (paired t-test + ANOVA) is reported for the chosen outcome; alternative models or populations are not presented. |

**Domain 5 judgment: Some concerns**
*Because no protocol was available (5.1 = NI), even though outcome/analysis selection seems unlikely, the algorithm yields “Some concerns.”*

**OVERALL RISK-OF-BIAS JUDGMENT: Some concerns**

*Domains 1 and 5 carry “Some concerns” while Domains 2–4 are Low risk; no domain is High risk, so overall the study has “Some concerns” for bias for the stated primary outcome.*

## Study 13

**Primary outcome assessed:** Need for endotracheal intubation / invasive mechanical ventilation.

**DOMAIN 1: Risk of Bias from the Randomization Process**

| **Signalling question** | **Code** | **Reasoning** |
| --- | --- | --- |
| **1.1 Was the allocation sequence random?** | **Y** | “*Randomization was performed by the Chief Pharmacist, using QuickCalcs – Random number calculators…*” |
| **1.2 Was the allocation sequence concealed until participants were enrolled and assigned?** | **Y** | “*Eligible patients were allocated to receive medication in individually numbered packs. The Chief Pharmacist kept the code … until the end of the study.*” |
| **1.3 Did baseline differences suggest a problem with the randomization process?** | **N** | “*Baseline characteristics were very similar between the two groups…*” (Table 1 shows non-significant p-values). |

**Domain 1 Judgment:** **Low risk**
**Justification:** Concealment = Y; sequence random = Y → proceed to 1.3; 1.3 = N → Low risk.

**DOMAIN 2: Risk of Bias Due to Deviations from Intended Interventions**

*(effect of assignment to intervention)*

| **Signalling question** | **Code** | **Reasoning** |
| --- | --- | --- |
| **2.1 Were participants aware of their assigned intervention?** | **N** | “*Double-blind, randomized, placebo-controlled trial…*” |
| **2.2 Were carers/personnel aware of the assignments?** | **N** | Same quote as 2.1; no indication blinding was broken. |
| **2.3 If 2.1/2.2 ≠ N: Were there deviations?** | — | Not triggered (both 2.1 & 2.2 = N). |
| **2.6 Was an appropriate analysis used (effect of assignment)?** | **Y** | “*Primary efficacy analysis was on an intention-to-treat basis and included all the patients who had undergone randomization.*” |

**Domain 2 Judgment:** **Low risk**
**Justification:** Blinding intact (Part 1 = Low risk) and ITT used (Part 2 = Low risk).

**DOMAIN 3: Risk of Bias Due to Missing Outcome Data**

| **Signalling question** | **Code** | **Reasoning** |
| --- | --- | --- |
| **3.1 Were outcome data available for nearly all participants?** | **Y** | 140 randomized; 135 (96.4 %) included in analysis, primary outcome reported for all analysed participants. |

**Domain 3 Judgment:** **Low risk**
**Justification:** > 95 % of randomized participants contributed primary-outcome data.

**DOMAIN 4: Risk of Bias in Measurement of the Outcome**

| **Signalling question** | **Code** | **Reasoning** |
| --- | --- | --- |
| **4.1 Was the method of measuring the outcome inappropriate?** | **N** | Intubation/mechanical ventilation is an objective clinical event. |
| **4.2 Could measurement differ between groups?** | **PN** | No indication of different procedures; the decision is clinical but objective; trial was double-blind. |
| **4.3 Were outcome assessors aware of intervention?** | **N** | Blinding encompassed treating staff (“double-blind”). |

**Domain 4 Judgment:** **Low risk**
**Justification:** Objective outcome, identical procedures, assessors blinded.

**DOMAIN 5: Risk of Bias in Selection of the Reported Result**

| **Signalling question** | **Code** | **Reasoning** |
| --- | --- | --- |
| **5.2 Result likely selected from multiple outcome measurements?** | **PN** | Only one clear way to define primary outcome (need for intubation); no alternative measures described. |
| **5.3 Result likely selected from multiple eligible analyses?** | **PN** | Single ITT analysis reported; no unexplained subgroup or model selection. |
| **5.1 Was the analysis plan pre-specified?** | **PY** | Trial was registered before enrolment (REBEC # U1111-1250-356) but separate SAP not provided. |

**Domain 5 Judgment:** **Low risk**
**Justification:** 5.2 & 5.3 = PN (not Y/PY); 5.1 = PY → Low risk.

**Overall Risk-of-Bias Judgment: Low risk**

All five domains were judged Low risk; no high-risk concerns identified.

## Study 14

**Revised Cochrane Risk-of-Bias (RoB 2) Assessment – Primary Outcome: Average post-randomization weight-gain (g / kg / day) to 40 weeks’ CGA**

**DOMAIN 1 – Bias arising from the randomization process**

| **Signalling question** | **Answer** | **Reasoning** |
| --- | --- | --- |
| **1.1 Was the allocation sequence random?** | **Y** | “*…randomized, using random numbers, generated using a* ***web-based random number generator***” |
| **1.2 Was the allocation sequence concealed until participants were enrolled & assigned?** | **Y** | “*…placed in a* ***serially numbered, sealed, opaque envelope*** *and … opened after taking informed consent*” |
| **1.3 Did baseline differences suggest a problem with randomization?** | **N** | Table 1 shows key characteristics (gestational age, birth-weight, sex, IUGR) balanced; only caesarean rate differed (93 % vs 79.7 %, p = 0.02) but unlikely to influence neonatal weight-gain. |

**Domain 1 Judgment: Low risk**
*Allocation sequence was random (1.1 = Y) and adequately concealed (1.2 = Y); baseline covariates essentially balanced (1.3 = N) → Low risk.*

**DOMAIN 2 – Bias due to deviations from intended interventions (effect of assignment)**

| **Signalling question** | **Answer** | **Reasoning** |
| --- | --- | --- |
| **2.1 Were participants aware of their assignment?** | **Y** | Open-label ward-based care; no blinding of mothers is possible. |
| **2.2 Were carers/personnel aware?** | **Y** | Nurses supervised KMC ward; study describes no blinding. |
| **2.3 Were there deviations from intended intervention due to the trial context?** | **N** | No cross-overs or non-protocol co-interventions reported; both groups received identical feeding protocol. |
| **2.6 Was an appropriate analysis used (effect of assignment)?** | **Y** | “*Intention-to-treat analysis was performed.*” |

*(2.4, 2.5, 2.7 not triggered)*

**Domain 2 Judgment: Low risk**
*Although unblinded (2.1 / 2.2 = Y), no protocol-deviation linked to awareness was reported (2.3 = N) and analysis was ITT (2.6 = Y) → Part 1 Low, Part 2 Low → Overall Low risk.*

**DOMAIN 3 – Bias due to missing outcome data**

| **Signalling question** | **Answer** | **Reasoning** |
| --- | --- | --- |
| **3.1 Were outcome data available for nearly all participants?** | **Y** | 140 randomized ➔ 136 analysed (97 % complete: flowchart shows *3* KMC + *1* CMC lost). |

*(3.2 – 3.4 not triggered)*

**Domain 3 Judgment: Low risk**
*> 95 % outcome data obtained; little scope for bias.*

**DOMAIN 4 – Bias in measurement of the outcome**

| **Signalling question** | **Answer** | **Reasoning** |
| --- | --- | --- |
| **4.1 Was the method of measuring the outcome inappropriate?** | **N** | Weight measured on **electronic scale (± 5 g)**; standard anthropometry described. |
| **4.2 Could measurement differ between groups?** | **N** | Same instruments & schedule applied to both groups. |
| **4.3 Were outcome assessors aware of intervention?** | **N** | “*The physician assessing the growth outcomes was* ***blinded*** *to the intervention group.*” |

*(4.4-4.5 not triggered)*

**Domain 4 Judgment: Low risk**
*Objective outcome, identical procedures, blinded assessor → Low risk.*

**DOMAIN 5 – Bias in selection of the reported result**

| **Signalling question** | **Answer** | **Reasoning** |
| --- | --- | --- |
| **5.2 Result likely selected from multiple outcome measurements?** | **PN** | Primary outcome (weight-gain to 40 wks) pre-specified; although growth can be assessed many ways, authors report all planned anthropometric endpoints. |
| **5.3 Result likely selected from multiple analyses?** | **PN** | Simple t-tests/chi-square; no evidence of multiple modelling or subgroup-driven selection. |
| **5.1 Was analysis plan pre-specified?** | **NI** | No protocol/SAP referenced. |

**Domain 5 Judgment: Some concerns**
*With 5.2 & 5.3 = PN (no clear multiplicity) we rely on 5.1; absence of a pre-dated analysis plan (5.1 = NI) yields Some concerns.*

**Overall Risk-of-Bias Judgment: Some concerns**

*Domains 1-4 are Low risk; Domain 5 has Some concerns regarding pre-specification of analyses → Overall* ***Some concerns****.*

## Study 15

**Primary outcome assessed:** Overall survival
**Trial assessed:** STAMPEDE multi-arm, multistage RCT comparing SOC, SOC + zoledronic acid, SOC + docetaxel, SOC + zoledronic acid + docetaxel.

**DOMAIN 1 – Risk of bias arising from the randomisation process**

| **Signalling question** | **Answer** | **Reasoning (direct quotes)** |
| --- | --- | --- |
| 1.1 Was the allocation sequence random? | **Y** | “Patients were randomised centrally using a computerised algorithm, developed and maintained by the trials unit. Minimisation with a random element of 80% was used” |
| 1.2 Was the allocation sequence concealed until participants were enrolled and assigned? | **Y** | Same centrally-controlled computer algorithm (no local access to the schedule) |
| 1.3 Did baseline differences suggest a problem with the randomisation process? | **N** | Baseline Table 1 shows near-identical distributions for age, PSA, metastatic status, Gleason grade, comorbidity, etc. across all four groups – no clinically important imbalances reported |

**Domain 1 judgement: Low risk**
*Allocation was centrally concealed (1.2 = Y) and explicitly random (1.1 = Y); baseline covariates were balanced (1.3 = N) → Low risk.*

**DOMAIN 2 – Risk of bias due to deviations from intended interventions (effect of assignment)**

| **Signalling question** | **Answer** | **Reasoning** |
| --- | --- | --- |
| 2.1 Were participants aware of their assigned intervention? | **Y** | “There was no blinding to treatment allocation” |
| 2.2 Were carers / trial personnel aware? | **Y** | Same statement (open-label) |
| 2.3 Were there deviations from intended intervention arising from the trial context? | **PN** | High adherence reported: 77 % of SOC + Doc and 71 % of SOC + ZA + Doc received all six planned cycles; non-starts or early stops were mostly toxicity- or preference-related, not due to open-label status |
| 2.4 If yes/PY to 2.3, were these deviations likely to affect outcome? | *Not triggered* |  |
| 2.5 Were deviations balanced between groups? | *Not triggered* |  |
| 2.6 Was an appropriate analysis used (e.g. ITT)? | **Y** | “All patients are included in the efficacy analyses according to allocated treatment on an intention-to-treat basis” |
| 2.7 If 2.6 = N/PN/NI, is there potential for substantial bias? | *Not triggered* |  |

**Domain 2 judgement: Low risk**
*Open-label (2.1, 2.2 = Y) but no material, outcome-related protocol deviations (2.3 = PN) and ITT analysis used (2.6 = Y) → Part 1 = Low, Part 2 = Low → Low risk.*

**DOMAIN 3 – Risk of bias due to missing outcome data**

| **Signalling question** | **Answer** | **Reasoning** |
| --- | --- | --- |
| 3.1 Were outcome data available for nearly all participants? | **Y** | For overall survival, vital status was known for 96-97 % of randomised men; e.g., in the SOC arm only 75/1184 (6 %) were alive but without a data point in the preceding year . Median follow-up 43 months with reverse–censoring approach |
| 3.2–3.4 | *Not triggered* (3.1 = Y) |  |

**Domain 3 judgement: Low risk**
*Very small loss to follow-up for an objective outcome; no evidence that missingness biased results.*

**DOMAIN 4 – Risk of bias in measurement of the outcome**

| **Signalling question** | **Answer** | **Reasoning** |
| --- | --- | --- |
| 4.1 Was the outcome measurement method inappropriate? | **N** | Overall survival determined from death registry/hospital records – an objective, standard measure. |
| 4.2 Could measurement differ between groups? | **N** | Same national and site procedures applied to all groups. |
| 4.3 Were outcome assessors aware of intervention? | **PN** | Cause-of-death reviewers were masked, but overall survival itself is objective and needs no judgement |
| 4.4 Could any awareness influence assessment? | **PN** | Death is not susceptible to observer bias. |
| 4.5 Is it likely that assessment was influenced? | *Not triggered* |  |

**Domain 4 judgement: Low risk**
*Objective outcome measured identically for all participants; any lack of blinding cannot plausibly influence survival ascertainment.*

**DOMAIN 5 – Risk of bias in selection of the reported result**

| **Signalling question** | **Answer** | **Reasoning** |
| --- | --- | --- |
| 5.1 Was the analysis that produced the result pre-specified? | **PY** | Protocol and trial registration noted; primary survival analysis follows the stated Cox/log-rank plan |
| 5.2 Was the result likely selected from multiple outcome measurements? | **N** | Overall survival is single, clearly defined (“time from randomisation to death from any cause”) |
| 5.3 Was the result likely selected from multiple eligible analyses? | **PN** | One primary ITT analysis reported; sensitivity analyses consistent, no evidence of selective reporting. |

**Domain 5 judgement: Low risk**
*Single pre-specified primary outcome and standard analysis; little scope for selective reporting.*

**Overall risk-of-bias judgement: Low risk**

*All five domains judged Low risk → overall confidence in the survival effect estimates is high.*

## Study 16

**Primary outcome assessed:** Exclusive breastfeeding (EBF) status at 6 months postpartum

**DOMAIN 1 — Risk of bias arising from the randomization process**

| **Signalling question** | **Code** | **Direct‐quote justification** |
| --- | --- | --- |
| **1.1 Was the allocation sequence random?** | PY | “*…they were randomized into three groups by using* ***block randomization***” – a random method is named but not fully described. |
| **1.2 Was the allocation sequence concealed until participants were enrolled and assigned?** | NI | The article gives **no information** about any concealment method (e.g., sealed envelopes or central randomization). |
| **1.3 Did baseline differences suggest a problem with the randomization process?** | PY | Several important baseline characteristics differ significantly across groups (e.g., maternal age **p < 0.001**, occupation, income, education) . |

**Domain 1 judgment:** **High risk**
**Justification:** 1.2 = NI → need 1.3. Because notable imbalances were present (1.3 = PY), the algorithm yields **High risk**.

**DOMAIN 2 — Risk of bias due to deviations from intended interventions (effect of assignment)**

| **Signalling question** | **Code** | **Direct‐quote justification** |
| --- | --- | --- |
| **2.1 Were participants aware of their assigned intervention?** | Y | Mothers either *“did not listen to music”* or *“listened to music during cesarean section …”* — obvious, open-label. |
| **2.2 Were carers or trial personnel aware of the intervention?** | Y | Operating-room staff provided the ear-phones/music; no blinding is mentioned . |
| **2.3 Were there deviations from intended intervention due to the trial context?** | N | No cross-overs or other deviations are reported. |
| **2.6 Was an appropriate analysis used to estimate the effect of assignment?** | N | The trial excluded 22/207, 21/206, etc., post-randomization “due to failure to follow-up” and analysed 185 per group, i.e., not an ITT analysis . |
| **2.7 Potential for substantial bias from the analysis?** | PN | Attrition was ≈10 % and balanced; impact probably limited. |

**Domain 2 judgment:** **Some concerns**
**Justification:** Awareness was unblinded but no deviations occurred (Part 1 = Low risk). The analysis was not ITT (Part 2 = Some concerns) → overall **Some concerns**.

**DOMAIN 3 — Risk of bias due to missing outcome data**

| **Signalling question** | **Code** | **Direct‐quote justification** |
| --- | --- | --- |
| **3.1 Were outcome data available for nearly all participants?** | N | 620 randomized but only 555 analysed (185 × 3); e.g., *“21/206 cases were excluded … failure to follow-up”* ; loss > 5 %. |
| **3.2 Evidence that results were not biased by missing data?** | N | No sensitivity or imputation analyses reported. |
| **3.3 Could missingness depend on true outcome?** | PY | Mothers lost to follow-up may have stopped EBF; plausible relationship. |
| **3.4 Is it likely that this occurred?** | PY | Follow-up relied on telephone contact; non-responders could differ in breastfeeding success. |

**Domain 3 judgment:** **High risk**
**Justification:** 3.1 = N → 3.2 = N → 3.3 = PY → 3.4 = PY → **High risk**.

**DOMAIN 4 — Risk of bias in measurement of the outcome**

| **Signalling question** | **Code** | **Direct‐quote justification** |
| --- | --- | --- |
| **4.1 Was the method of measuring the outcome inappropriate?** | N | EBF status is a standard outcome; mothers were followed up at prespecified time-points “*7 days, 14 days … and 6 months*” . |
| **4.2 Could measurement differ between groups?** | N | Same follow-up schedule applied to all groups. |
| **4.3 Were outcome assessors aware of the intervention?** | NI | The paper does not say who collected follow-up data or whether they were blinded. |
| **4.4 Could such awareness influence the outcome?** | PY | EBF is self-reported and subject to interviewer or respondent bias. |
| **4.5 Is it likely that assessment was influenced?** | PN | Any influence is possible but probably small because the outcome is participant-reported rather than assessor-rated. |

**Domain 4 judgment:** **Some concerns**
**Justification:** 4.3 = NI with 4.2 = N → proceed; 4.4 = PY and 4.5 = PN → **Some concerns**.

**DOMAIN 5 — Risk of bias in selection of the reported result**

| **Signalling question** | **Code** | **Direct‐quote justification** |
| --- | --- | --- |
| **5.2 Result selected from multiple outcome measurements?** | PN | All planned EBF time-points (7 d to 6 m) are reported in Table 3 . |
| **5.3 Result selected from multiple eligible analyses?** | PN | Only simple group comparisons (Chi-square) are presented; no alternate models reported. |
| **5.1 Analysis pre-specified?** | PN | No protocol/SAP referenced; timing unclear. |

**Domain 5 judgment:** **Some concerns**
**Justification:** 5.2 & 5.3 = PN → look at 5.1 (PN) → **Some concerns**.

**OVERALL risk-of-bias judgment**

**Overall judgment: High risk**
**Brief justification:** High risk in Domain 1 (randomization) and Domain 3 (missing data) outweigh “Some concerns” in Domains 2, 4, 5.

## Study 17

**Primary outcome evaluated**

*Numeric Rating Scale (NRS) pain score during patient positioning for spinal anaesthesia.*

**DOMAIN 1: Risk of Bias from the Randomization Process**

| **Signalling question** | **Judgment** | **Reasoning (direct quote)** |
| --- | --- | --- |
| **1.1 Was the allocation sequence random?** | **PN** | “Patients were **randomly assigned, using a sealed envelope method**, into 1 of 2 groups” ― the term *randomly* is used, but no description of how the sequence was generated. |
| **1.2 Was the allocation sequence concealed until participants were enrolled and assigned?** | **PN** | The same sentence reports sealed envelopes but gives **no details** on opacity, sequential numbering, or central control. |
| **1.3 Did baseline differences suggest a problem with randomization?** | **N** | “Both groups of patients were **comparable regarding demographic data, type of fracture …**” (Table 1). |

**Domain 1 judgment: High risk**
**Justification:** 1.2 = PN ⇒ by the algorithm the domain is High risk regardless of 1.1/1.3.

**DOMAIN 2: Risk of Bias due to Deviations from Intended Interventions**

*(effect of assignment to intervention)*

| **Signalling question** | **Judgment** | **Reasoning** |
| --- | --- | --- |
| **2.1 Were participants aware of their assigned intervention?** | **Y** | “**Each patient was aware of his/her group allocation** because we considered a placebo injection … not acceptable.” |
| **2.2 Were carers or trial personnel aware of the intervention assignments?** | **Y** | The clinicians who administered IV fentanyl vs FICB necessarily knew the allocation; no blinding is reported for them. |
| **2.3 Were there deviations from intended intervention due to the trial context?** | **PN** | No cross-overs or protocol violations are mentioned; groups received the interventions as planned. |
| *2.4–2.5* | *Not triggered* | 2.3 = PN. |
| **2.6 Was an appropriate analysis used to estimate the effect of assignment?** | **PN** | The analysis section reports only that “Data were analyzed using SPSS …”, with no statement of intention-to-treat or similar. |
| **2.7 Was there potential for substantial bias due to inappropriate analysis?** | **PN** | One participant withdrew (41/42 analysed); the exclusion is unlikely to materially bias results. |

**Domain 2 judgment: Some concerns**
**Justification:** Part 1 Low risk (2.3 = PN); Part 2 Some concerns (2.6 = PN, 2.7 = PN) ⇒ overall Some concerns.

**DOMAIN 3: Risk of Bias due to Missing Outcome Data**

| **Signalling question** | **Judgment** | **Reasoning** |
| --- | --- | --- |
| **3.1 Were outcome data available for nearly all participants?** | **Y** | 42 randomized, 41 analysed (≈ 98%). |

*3.2–3.4 not required.*

**Domain 3 judgment: Low risk**
**Justification:** > 95 % complete outcome data.

**DOMAIN 4: Risk of Bias in Measurement of the Outcome**

| **Signalling question** | **Judgment** | **Reasoning** |
| --- | --- | --- |
| **4.1 Was the method of measuring the outcome inappropriate?** | **N** | Pain was assessed with the 0–10 **Numeric Rating Scale**, a validated measure in hip-fracture patients. |
| **4.2 Could measurement of the outcome differ between groups?** | **N** | Both groups used identical NRS assessments at the same time-points. |
| **4.3 Were outcome assessors aware of intervention assignments?** | **N** | “The study was **blind from the aspect of the anesthesiologist who recorded the NRS** … he/she entered the OR after the analgesic intervention had taken place.” |

*4.4–4.5 not triggered.*

**Domain 4 judgment: Low risk**
**Justification:** Appropriate, uniform measurement with blinded assessors.

**DOMAIN 5: Risk of Bias in Selection of the Reported Result**

| **Signalling question** | **Judgment** | **Reasoning** |
| --- | --- | --- |
| **5.2 Result selected from multiple outcome measurements?** | **PN** | Pain was consistently measured with NRS; no alternative scales reported. |
| **5.3 Result selected from multiple eligible analyses?** | **PN** | Only one primary analysis method is described; no evidence of multiple models. |
| **5.1 Was the analysis plan pre-specified?** | **PY** | Trial registered on ClinicalTrials.gov (NCT02037633), implying prespecification, but no dated SAP is provided. |

**Domain 5 judgment: Low risk**
**Justification:** 5.2 = PN & 5.3 = PN → evaluate 5.1; 5.1 = PY ⇒ Low risk.

**Overall Risk-of-Bias Judgment: High risk**

**Rationale:** Domain 1 is High risk owing to inadequate information on sequence generation and concealment; although other domains are Low risk or Some concerns, a single High-risk domain mandates an Overall judgment of High risk.

## Study 18

**Revised Cochrane RoB 2 assessment
RCT evaluated:** Dobkin et al., 2020 – Telephone-based CBT vs. TAU for depression in Parkinson’s disease
**Primary outcome assessed:** Change in Hamilton Depression Rating Scale (HAM-D) score

**Domain 1 – Bias arising from the randomisation process**

| **Signalling question** | **Answer** | **Direct evidence** |
| --- | --- | --- |
| **1.1 Was the allocation sequence random?** | **Y** | “M.A.G. assigned participants to groups **using multiple-password-protected randomization software** to which he retained sole access” |
| **1.2 Was the allocation sequence concealed until participants were enrolled and assigned?** | **Y** | The statistician “retained sole access” and was “**blind to all other baseline data**” |
| **1.3 Did baseline differences suggest a problem with the randomisation process?** | **N** | “**The groups’ baseline clinical and demographic factors did not differ** (table 1)” |

**Domain 1 judgement: Low risk**
**Justification:** Allocation was computer-generated and concealed (1.1 = Y, 1.2 = Y); groups were well balanced at baseline (1.3 = N) → Low risk.

**Domain 2 – Bias due to deviations from intended interventions (effect of *assignment*)**

| **Signalling question** | **Answer** | **Direct evidence** |
| --- | --- | --- |
| **2.1 Were participants aware of their assigned intervention?** | **Y** | Participants received weekly telephone CBT sessions; no blinding of participants is reported |
| **2.2 Were carers or trial personnel aware of the intervention?** | **Y** | Therapists delivered CBT; no blinding reported |
| **2.3 Were there deviations from intended intervention due to the trial context?** | **PN** | Paper does not describe any cross-overs or differential co-interventions; TAU continued as usual, and sessions were protocolised. |
| **2.4–2.5 (only if 2.3 = Y/PY)** | — | Not triggered |
| **2.6 Was an appropriate analysis used to estimate the effect of assignment?** | **Y** | “Data were analysed … **using an intention-to-treat approach**” |
| **2.7 (Potential bias from inappropriate analysis)** | — | Not triggered |

**Domain 2 judgement: Low risk**
**Justification:** Although participants and therapists were unblinded (2.1–2.2 = Y), no meaningful protocol deviations related to that awareness were reported (2.3 = PN). Analysis was ITT (2.6 = Y). Part 1 = Low, Part 2 = Low → Domain 2 = Low risk.

**Domain 3 – Bias due to missing outcome data**

| **Signalling question** | **Answer** | **Direct evidence** |
| --- | --- | --- |
| **3.1 Were outcome data available for nearly all participants?** | **N** | 63 of 72 randomised participants completed the study (**88 % retained**) |
| **3.2 Evidence that results were not biased by missing data?** | **PY** | Mixed-effects models under an ITT framework were used to include all available data |
| **3.3 Could missingness depend on true outcome value?** | **PN** | Retention high and balanced; no indication that dropout related to depression severity. |
| **3.4 Is it likely that it did?** | — | Not triggered |

**Domain 3 judgement: Low risk**
**Justification:** Although data were missing for >5 % (3.1 = N), the ITT mixed-model analysis reduces bias (3.2 = PY) and no evidence suggests outcome-dependent attrition (3.3 = PN) → Low risk.

**Domain 4 – Bias in measurement of the outcome**

| **Signalling question** | **Answer** | **Direct evidence** |
| --- | --- | --- |
| **4.1 Was the method of measuring the outcome inappropriate?** | **N** | Primary outcome measured with the **validated HAM-D** scale |
| **4.2 Could measurement differ between groups?** | **N** | Same instrument and schedule used across groups. |
| **4.3 Were outcome assessors aware of intervention?** | **N** | “**Condition-blind raters assessed outcomes**” |
| **4.4–4.5 (only if 4.3 = Y/PY/NI)** | — | Not triggered |

**Domain 4 judgement: Low risk**
**Justification:** Validated measure, identical procedures, and blinded assessors → Low risk.

**Domain 5 – Bias in selection of the reported result**

| **Signalling question** | **Answer** | **Direct evidence** |
| --- | --- | --- |
| **5.2 Result selected from multiple outcome measurements?** | **PN** | Only HAM-D reported as primary; all scheduled time-points presented. |
| **5.3 Result selected from multiple eligible analyses?** | **PN** | Single pre-specified mixed-model analysis described; no alternative analyses presented. |
| **5.1 Was the analysis pre-specified before unblinded data access?** | **PY** | Authors state that “Primary outcomes and inclusion/exclusion criteria were **clearly defined a priori**”; protocol and statistical analysis plan are available on request |

**Domain 5 judgement: Low risk**
**Justification:** No evidence of selective measurement or analysis (5.2/5.3 = PN) and analysis appears pre-specified (5.1 = PY) → Low risk.

**Overall risk-of-bias judgement: Low risk**

All five domains were judged at Low risk; therefore, the overall risk of bias for the HAM-D outcome in this trial is **Low**.

## Study 19

**Primary outcome assessed:** *Time-to-PCR-negativity (Days-to-Negative, DTN)*

**DOMAIN 1 – Risk of Bias arising from the Randomisation Process**

| **Signalling question** | **Answer** | **Direct-quote justification** |
| --- | --- | --- |
| **1.1 Was the allocation sequence random?** | **Y** | “**Randomisation:** Computers were used to generate random numbers which were employed for the allocation.” |
| **1.2 Was the allocation sequence concealed until participants were enrolled and assigned?** | **PY** | “The study medications were in labelled envelopes prepared by pharmacy, held by the nursing staff and administered by them without the knowledge of the clinical research team… The dispensing pharmacist was the **ONLY** one with knowledge of the group allocation.” |
| **1.3 Did baseline differences suggest a problem with the randomisation process?** | **Y** | Table 1 shows significant imbalances in baseline viral-load proxies (Ct ORF *p = 0.049*, Ct N *p = 0.02*). |

**Domain 1 judgment:** *Some concerns*
**Justification:** Allocation was probably concealed (1.2 = PY) and the sequence was random (1.1 = Y), but meaningful baseline imbalance was present (1.3 = Y).

**DOMAIN 2 – Risk of Bias due to Deviations from Intended Interventions (effect of assignment)**

| **Signalling question** | **Answer** | **Direct-quote justification** |
| --- | --- | --- |
| **2.1 Were participants aware of their assigned intervention?** | **N** | “Neither the patients nor the investigators were aware of what was administered until the code was broken at the end of the study.” |
| **2.2 Were carers/personnel aware of the intervention assignments?** | **N** | Same statement as above; drugs dispensed by pharmacist, care team blinded. |
| 2.3–2.5 | *Not triggered* (blinding judged adequate) |  |
| **2.6 Was an appropriate analysis used to estimate the effect of assignment?** | **PN** | No explicit intention-to-treat statement; analysis tables include *N = 60* although *62* were randomised (one withdrawal, two missing tests). |
| **2.7 Is there potential for substantial bias due to inappropriate analysis?** | **PN** | Excluding two participants is unlikely to materially bias the result, but the absence of an ITT declaration introduces some uncertainty. |

**Domain 2 judgment:** *Some concerns*
**Justification:** Blinding adequate (Part 1 = Low risk). Analysis not clearly ITT (2.6 = PN) → Part 2 = Some concerns → overall *Some concerns*.

**DOMAIN 3 – Risk of Bias due to Missing Outcome Data**

| **Signalling question** | **Answer** | **Direct-quote justification** |
| --- | --- | --- |
| **3.1 Were outcome data available for nearly all participants?** | **Y** | “Sixty-three patients… were randomised… There was **one withdrawal**, thus **sixty-two** patients completed the study. Tests were **not completed in two cases**.” (≥ 95 % data available). |

**Domain 3 judgment:** *Low risk*
**Justification:** > 95 % of randomised participants had observed primary-outcome data.

**DOMAIN 4 – Risk of Bias in Measurement of the Outcome**

| **Signalling question** | **Answer** | **Direct-quote justification** |
| --- | --- | --- |
| **4.1 Was the method of measuring the outcome inappropriate?** | **N** | “COVID-19 test was by **RT-PCR** testing for three genes using a GeneXpert machine.” |
| **4.2 Could measurement differ between groups?** | **N** | Same RT-PCR procedure for all arms. |
| **4.3 Were outcome assessors aware of intervention?** | **N** | “Virological assays were done in a separate building on samples **labelled only with the patients’ trial number**.” |

**Domain 4 judgment:** *Low risk*
**Justification:** Objective lab outcome measured identically across arms by blinded staff.

**DOMAIN 5 – Risk of Bias in Selection of the Reported Result**

| **Signalling question** | **Answer** | **Direct-quote justification** |
| --- | --- | --- |
| **5.2 Is the result likely selected from multiple outcome measurements?** | **PN** | Only one primary metric of virological clearance (DTN) is described and reported. |
| **5.3 Is the result likely selected from multiple eligible analyses?** | **PN** | Analysis methods (ANOVA, Kaplan-Meier, Cox) align with stated objectives; no unreported alternative analyses indicated. |
| **5.1 Was the analysis plan pre-specified?** | **PY** | Trial registered (*ISRCTN40302986*) but protocol/SAP not presented in the article. |

**Domain 5 judgment:** *Low risk*
**Justification:** No evidence of selective outcome or analytic reporting; lack of a public SAP gives only minor doubt.

**OVERALL RISK-OF-BIAS JUDGMENT**

**Overall judgment:** *Some concerns*
**Brief rationale:** Domains 1 and 2 show “Some concerns” (baseline imbalance and absence of explicit ITT), while Domains 3-5 are Low risk; no domain is High risk.

## Study 20

**Primary outcome assessed:** Percentage change in body weight at 6 months (pre-specified primary end point in the article).

**DOMAIN 1 – Bias arising from the randomization process**

| **Signalling question** | **Answer** | **Direct quote / justification** |
| --- | --- | --- |
| 1.1 Was the allocation sequence random? | **PY** | “Randomization was performed by a *stratified random sampling* procedure by sex, age, BMI and HbA1c level.” |
| 1.2 Was the allocation sequence concealed until participants were enrolled and assigned? | **PN** | The *Randomization and Blinding* section describes the sequence but gives **no information on any concealment method** (e.g., central allocation or sealed envelopes). |
| 1.3 Did baseline differences suggest a problem with the randomization process? | **N** | Baseline table shows closely balanced age, sex, BMI and other key variables across groups. |

**Domain 1 judgement: High risk**
*Algorithm:* 1.2 = PN → Domain 1 = High risk (no further steps).

**DOMAIN 2 – Bias due to deviations from intended interventions (effect of assignment)**

| **Signalling question** | **Answer** | **Direct quote / justification** |
| --- | --- | --- |
| 2.1 Were participants aware of their assigned intervention? | **Y** | “Participants were **not blinded**.” |
| 2.2 Were carers/personnel aware of the intervention assignments? | **Y** | Dietitians delivered and monitored the dietary prescriptions; no blinding is reported. |
| 2.3 Were there deviations from intended intervention due to the trial context? | **N** | Adherence was monitored but no cross-over or additional co-interventions are reported; deviations reflect normal non-adherence, not awareness. |
| 2.6 Was an appropriate analysis used to estimate the effect of assignment? | **Y** | “We conducted an **intention-to-treat analysis**, which included data from all 75 participants who underwent randomization.” |

*(2.1/2.2 = Y ⇒ assess 2.3; 2.3 = N ⇒ Part 1 Low risk. 2.6 = Y ⇒ Part 2 Low risk)*

**Domain 2 judgement: Low risk**
Part 1 = Low risk and Part 2 = Low risk → Domain 2 = Low risk.

**DOMAIN 3 – Bias due to missing outcome data**

| **Signalling question** | **Answer** | **Direct quote / justification** |
| --- | --- | --- |
| 3.1 Were outcome data available for nearly all participants? | **N** | Six of 75 participants (8%) were lost to follow-up (attrition ≤ 12 % per arm). |
| 3.2 Is there evidence that results were not biased by missing data? | **Y** | “Sensitivity analysis **using multiple imputation** … conclusions for body weight and HbA1c did not change.” |

*(3.1 = N → evaluate 3.2; 3.2 = Y → Domain 3 = Low risk)*

**Domain 3 judgement: Low risk**

**DOMAIN 4 – Bias in measurement of the outcome**

| **Signalling question** | **Answer** | **Direct quote / justification** |
| --- | --- | --- |
| 4.1 Was the method of measuring the outcome inappropriate? | **N** | Body weight was measured objectively at *seven scheduled clinic visits*. |
| 4.2 Could measurement differ between groups? | **N** | All groups attended the same research centre on identical schedules. |
| 4.3 Were outcome assessors aware of intervention? | **Y** | No assessor blinding is reported. |
| 4.4 Could awareness have influenced outcome assessment? | **PN** | Weight is an objective digital-scale measure, unlikely to be influenced by assessor judgement. |

*(4.1 N & 4.2 N; 4.3 Y but 4.4 PN ⇒ Low risk)*

**Domain 4 judgement: Low risk**

**DOMAIN 5 – Bias in selection of the reported result**

| **Signalling question** | **Answer** | **Direct quote / justification** |
| --- | --- | --- |
| 5.2 Result selected from multiple outcome measurements? | **N** | The article specifies **one primary outcome (6-month body-weight change)**; no alternative time-points or scales reported for that outcome. |
| 5.3 Result selected from multiple eligible analyses? | **N** | Only the pre-specified ITT analysis is presented; no unexplained subgroup or per-protocol analyses for the primary outcome. |
| 5.1 Was the analysis plan pre-specified? | **Y** | “The full trial protocol and statistical analysis plan are provided in Supplement 1.” |

*(5.2 = N and 5.3 = N → evaluate 5.1; 5.1 = Y ⇒ Domain 5 = Low risk)*

**Domain 5 judgement: Low risk**

**Overall risk-of-bias judgement: High risk**

*Rationale:* Domain 1 is High risk because allocation concealment was not described (1.2 = PN). All other domains are Low risk, but **any single High-risk domain yields an overall High-risk rating in RoB 2.**

## Study 21

**RCT assessed**: *Comparative Evaluation of Indirect Pulp Therapy in Young Permanent Teeth using Biodentine and Theracal: A Randomized Clinical Trial* (Rahman & Goswami, 2021)

**DOMAIN 1 ― Risk of Bias *arising from the randomization process***

| **Signalling Question** | **Judgment** | **Direct-quote reasoning** |
| --- | --- | --- |
| **1.1 Was the allocation sequence random?** | **Y** | “*Simple randomization was done by asking the patients to choose one of the three differently colored balls which would determine the intervening material to be placed for IPT*.” |
| **1.2 Was the allocation sequence concealed until participants were enrolled and assigned?** | **Y** | The coloured-ball draw occurred *after* enrolment; assignment was unknown to investigators until the ball was picked: same sentence as above . |
| **1.3 Did baseline differences suggest a problem?** | **NI** | No baseline table comparing the three groups is presented; only overall sample characteristics are given . |

**Domain 1 Judgment: Low risk**
*Sequence generation was random (1.1 = Y) and concealed (1.2 = Y). With no information on baseline imbalance (1.3 = NI) the algorithm yields Low risk.*

**DOMAIN 2 ― Risk of Bias *due to deviations from intended interventions* (effect of assignment)**

| **Signalling Question** | **Judgment** | **Direct-quote reasoning** |
| --- | --- | --- |
| **2.1 Were participants aware of their assignment?** | **N** | “*The participant was blinded from the intervening material used*.” |
| **2.2 Were carers/personnel aware?** | **Y** | “*The clinician doing the excavation was* ***not*** *blinded since different materials require different manipulating instructions*.” |
| **2.3 Were there deviations due to the trial context?** | **N** | No cross-overs or protocol deviations are reported in the Methods or Results sections. |
| **2.4–2.5** | — | (not reached – 2.3 = N) |
| **2.6 Was an appropriate analysis used (ITT)?** | **N** | Outcomes at 24 mo were analysed on 54/60 teeth; drop-outs were excluded and no ITT analysis is described . |
| **2.7 Potential for substantial bias from analysis?** | **PN** | Exclusions were small (10 % per group) and balanced , so major bias is unlikely. |

**Domain 2 Judgment: Some concerns**
*Blinding of operators was impossible (2.2 = Y) but no deviations occurred (Part 1 = Low risk). However, analysis was not ITT (2.6 = N) giving Part 2 = Some concerns → overall “Some concerns”.*

**DOMAIN 3 ― Risk of Bias *due to missing outcome data***

| **Signalling Question** | **Judgment** | **Direct-quote reasoning** |
| --- | --- | --- |
| **3.1 Were outcome data available for nearly all participants?** | **N** | “*Two teeth each from Biodentine, Theracal and Dycal groups (10 % each) were dropped from the study because the patients failed to report for the follow up*.” (54/60 = 90 %) |
| **3.2 Evidence that results were not biased by missing data?** | **N** | No sensitivity analysis or imputation is reported. |
| **3.3 Could missingness depend on true outcome?** | **PN** | Drop-outs were evenly split across groups with no indication they were related to treatment failure . |
| **3.4 Is it likely?** | — | (not reached – 3.3 = PN) |

**Domain 3 Judgment: Low risk**
*Although 10 % of data were missing (3.1 = N), losses were small, balanced and unlikely related to outcomes (3.3 = PN) → Low risk.*

**DOMAIN 4 ― Risk of Bias *in measurement of the outcome***

| **Signalling Question** | **Judgment** | **Direct-quote reasoning** |
| --- | --- | --- |
| **4.1 Outcome measurement inappropriate?** | **N** | Success was defined by standard clinical signs plus radiographic criteria described in detail . |
| **4.2 Could measurement differ between groups?** | **N** | The same predefined criteria and timing were applied to all groups . |
| **4.3 Were assessors aware of intervention?** | **N** | “*The investigator assessing the signs and symptoms was unaware of the intervening material placed*.” |

**Domain 4 Judgment: Low risk**
*Validated, identical assessments with blinded evaluators.*

**DOMAIN 5 ― Risk of Bias *in selection of the reported result***

| **Signalling Question** | **Judgment** | **Direct-quote reasoning** |
| --- | --- | --- |
| **5.2 Result selected from multiple measurements?** | **PN** | Only one logical definition of “success” is reported at all scheduled time-points. |
| **5.3 Result selected from multiple analyses?** | **PN** | Single chi-square analysis; no alternative models presented. |
| **5.1 Analysis pre-specified?** | **PY** | Trial registration is reported: “*registered under CTRI (CTRI/2018/02/014782)*” , but protocol/SAP not provided. |

**Domain 5 Judgment: Low risk**
*With 5.2 = PN and 5.3 = PN, a probably pre-specified analysis (5.1 = PY) leads to Low risk.*

**Overall risk-of-bias judgment: Some concerns**

*All domains were Low risk except Domain 2 (analysis deviations) which had Some concerns; no domain was High risk → overall “Some concerns”.*

## Study 22

**Risk-of-bias (RoB 2) assessment for the HOPE trial – primary outcome
“Proportion of laboratory-confirmed COVID-19 within 6 months”**

**Domain 1 – Bias arising from the randomisation process**

| **Q-no** | **Judgment** | **Reasoning** |
| --- | --- | --- |
| **1.1** Was the allocation sequence random? | **Y** | Investigators used “*a computer-generated allocation sequence with permuted blocks of varying sizes*” . |
| **1.2** Was the allocation sequence concealed until participants were enrolled and assigned? | **PY** | The sequence was implemented through *centralised randomisation* , strongly implying concealment, although safeguards are not explicitly described. |
| **1.3** Did baseline differences suggest a problem with randomisation? | **N** | Baseline characteristics (age, sex, role, comorbidities, etc.) were “*comparable between the two groups*” (Table 1) . |

**Domain 1 judgement: Low risk**
*Allocation was computer-generated (1.1 = Y) and probably concealed (1.2 = PY); baseline covariates were balanced (1.3 = N).*

**Domain 2 – Bias due to deviations from intended interventions (effect of assignment)**

| **Q-no** | **Judgment** | **Reasoning** |
| --- | --- | --- |
| **2.1** Were participants aware of their assignment? | **Y** | Trial described as *open-label* . |
| **2.2** Were carers/personnel aware? | **Y** | Same open-label statement applies . |
| **2.3** Were there deviations arising because of the experimental context? | **N** | High adherence: “*Compliance with the intervention was high (87.4%);*” protocol violations in the control arm were discouraged and not reported . |
| **2.6** Was an appropriate analysis used (effect of assignment)? | **Y** | *“Analysis of the primary and secondary outcomes was performed in the intention-to-treat population.”* |

Part 1 → Low risk (2.1–2.3)  Part 2 → Low risk (2.6)

**Domain 2 judgement: Low risk**
*Although unblinded, no meaningful deviations occurred and ITT analysis was used.*

**Domain 3 – Bias due to missing outcome data**

| **Q-no** | **Judgment** | **Reasoning** |
| --- | --- | --- |
| **3.1** Were outcome data available for nearly all participants? | **Y** | Only **2/416** (0.5 %) were lost to follow-up; 414 were analysed . |

**Domain 3 judgement: Low risk**

**Domain 4 – Bias in measurement of the outcome**

| **Q-no** | **Judgment** | **Reasoning** |
| --- | --- | --- |
| **4.1** Was the outcome measurement method inappropriate? | **N** | Outcome required **laboratory confirmation by PCR or antibody test**, an objective standard . |
| **4.2** Could measurement differ between groups? | **N** | Same testing approach applied to both groups. |
| **4.3** Were outcome assessors aware of intervention? | **N** | Laboratory staff were not described as unblinded, but the test result is objective and not readily influenced. |

**Domain 4 judgement: Low risk**

**Domain 5 – Bias in selection of the reported result**

| **Q-no** | **Judgment** | **Reasoning** |
| --- | --- | --- |
| **5.2** Result selected from multiple outcome measurements? | **N** | Only one prespecified definition (PCR/antibody at 6 months) reported. |
| **5.3** Result selected from multiple analyses? | **N** | Single ITT analysis presented. |
| **5.1** Was the analysis plan pre-specified? | **Y** | *“The trial protocol … and statistical analysis plan were published a priori.”* |

**Domain 5 judgement: Low risk**

**Overall risk-of-bias judgement: Low risk**

All five domains were rated **Low risk**, giving high confidence that the estimated effect of hydroxychloroquine + PPE versus PPE alone on laboratory-confirmed COVID-19 infection is not meaningfully biased.

## Study 23

Below is a complete **RoB 2 (22 Aug 2019) assessment** for the primary outcome of **“incidence of breakthrough pain requiring clinician top-up”** in
*A randomized comparison of automated intermittent mandatory boluses with a basal infusion in combination with patient-controlled epidural analgesia for labor and delivery* (2010).

**DOMAIN 1 – Risk of bias arising from the randomization process**

| **Signalling question** | **Answer** | **Direct-quote–based justification** |
| --- | --- | --- |
| **1.1 Was the allocation sequence random?** | **Y** | “*Parturients were randomly allocated into two groups using* ***sealed opaque envelopes and computer-generated random number tables*** *by an independent assistant*” |
| **1.2 Was the allocation sequence concealed until participants were enrolled and assigned?** | **Y** | Same sentence as above explicitly describes **sealed opaque envelopes**, meeting robust concealment criteria. |
| **1.3 Did baseline differences suggest a problem with randomization?** | **N** | “*Baseline characteristics and pre-block obstetric data were* ***similar for parturients in both groups (Table 1).***” |

**Domain 1 judgment: Low risk**
*Justification: 1.2 = Y (concealed); 1.1 = Y (computer-generated); 1.3 = N → Low risk.*

**DOMAIN 2 – Risk of bias due to deviations from intended interventions**

(effect of **assignment** to intervention)

| **Signalling question** | **Answer** | **Direct-quote–based justification** |
| --- | --- | --- |
| **2.1 Were participants aware of their assigned intervention during the trial?** | **N** | “*Neither the* ***parturients*** *nor the anesthesiologists who monitored and collected post-block data were* ***aware of group assignments***.” |
| **2.2 Were carers or trial personnel aware of the intervention assignments?** | **N** | Same quotation indicates the treating anesthesiologists collecting outcomes were blinded. |
| 2.3 If Y/PY/NI to 2.1 or 2.2 → Were there deviations arising from the trial context? | — | Not applicable (both 2.1 and 2.2 = N). |
| **2.6 Was an appropriate analysis used to estimate the effect of assignment?** | **Y** | “*All* ***62 recruited parturients completed the study***” (no exclusions); analyses compared groups as randomized (e.g., Student t test, χ², Kaplan–Meier). |

*Part 1 judgment = Low risk (blinded, no deviations).*
*Part 2 judgment = Low risk (all randomized participants analysed).*

**Domain 2 judgment: Low risk**
*Justification: Part 1 Low + Part 2 Low → Domain 2 Low risk.*

**DOMAIN 3 – Risk of bias due to missing outcome data**

| **Signalling question** | **Answer** | **Direct-quote–based justification** |
| --- | --- | --- |
| **3.1 Were outcome data available for nearly all participants?** | **Y** | “*All 62 recruited parturients* ***completed the study.***” |

*Because > 95 % of participants had observed outcome data, later questions are not triggered.*

**Domain 3 judgment: Low risk**
*Justification: 3.1 = Y.*

**DOMAIN 4 – Risk of bias in measurement of the outcome**

| **Signalling question** | **Answer** | **Direct-quote–based justification** |
| --- | --- | --- |
| **4.1 Was the method of measuring the outcome inappropriate?** | **N** | The outcome (“incidence of breakthrough pain requiring physician top-up”) was prospectively defined and based on a **standardised VAS ≥ 3 cm plus documented top-up** procedure described under Methods. |
| **4.2 Could measurement of the outcome differ between groups?** | **N** | Both groups used identical patient-controlled epidural pumps and identical criteria for breakthrough pain/top-up. |
| **4.3 Were outcome assessors aware of the intervention?** | **N** | Assessors (monitoring anesthesiologists) were expressly blinded (see Domain 2 quotation). |

**Domain 4 judgment: Low risk**
*Justification: 4.1 = N; 4.2 = N → 4.3 considered; 4.3 = N → Low risk.*

**DOMAIN 5 – Risk of bias in selection of the reported result**

| **Signalling question** | **Answer** | **Direct-quote–based justification** |
| --- | --- | --- |
| **5.2 Is the result likely selected from multiple outcome measurements?** | **PN** | Only one definition of breakthrough pain (VAS ≥ 3 cm with clinician top-up) is presented; paper does not hint at alternate measurements. No protocol to verify. |
| **5.3 Is the result likely selected from multiple eligible analyses?** | **PN** | Standard unadjusted between-group analyses are reported; but absence of a registered SAP leaves some uncertainty. |
| **5.1 Was the analysis plan pre-specified before unblinded data access?** | **NI** | The article provides **no reference to a trial registry, protocol, or SAP**. |

**Domain 5 judgment: Some concerns**
*Justification: 5.2 = PN & 5.3 = PN (neither Y/PY); with 5.1 = NI → Some concerns.*

**Overall risk-of-bias judgment: Some concerns**

*Domains 1–4 = Low risk; Domain 5 = Some concerns (no pre-registered analysis) → Overall = Some concerns.*

## Study 24

**Risk-of-Bias (RoB 2.0, 22 Aug 2019) assessment**

**Trial assessed:** *Docosahexaenoic Acid and Bronchopulmonary Dysplasia in Preterm Infants* (N = 1 273)
**Primary outcome evaluated:** Incidence of physiological bronchopulmonary dysplasia (BPD) at 36 weeks’ post-menstrual age or discharge.

**Domain 1 – Bias arising from the randomisation process**

| **Signalling question** | **Judgment** | **Reasoning** |
| --- | --- | --- |
| **1.1** Was the allocation sequence random? | **Y** | “A **computer-generated randomisation schedule** that used balanced variable blocks of 2, 4, and 6 in a ratio of 1:2:1 was prepared by an independent statistician.” |
| **1.2** Was allocation sequence concealed until assignment? | **Y** | Trial emulsions were *pre-packed* to match the sequence; “**participants…clinical and research personnel were unaware of the randomisation assignments**. The intervention and control emulsions were identical in viscosity, colour, packaging, and labelling.” |
| **1.3** Did baseline differences suggest a problem? | **N** | “**Baseline characteristics…were similar in the two treatment groups** (Table 1).” |

**Domain 1 judgment:** **Low risk**
*Allocation was random (1.1 = Y), concealed (1.2 = Y) and groups were balanced (1.3 = N).*

**Domain 2 – Bias due to deviations from intended interventions (effect of assignment)**

| **Signalling question** | **Judgment** | **Reasoning** |
| --- | --- | --- |
| **2.1** Participants aware of assignment? | **N** | Blinding stated as above; identical emulsions. |
| **2.2** Carers / trial personnel aware? | **N** | Same blinding statement applies to carers/research staff. |
| **2.3–2.5** Deviations due to awareness, effect on outcome, balance? | Not triggered | Both 2.1 & 2.2 = N → no risk-increasing deviations considered. |
| **2.6** Appropriate analysis (effect of assignment)? | **Y** | “**Analyses were performed on an intention-to-treat basis according to a prespecified statistical analysis plan.**” |
| **2.7** Substantial bias from inappropriate analysis? | Not triggered | 2.6 = Y. |

**Domain 2 judgment:** **Low risk**
*Effective blinding with ITT analysis; no evidence of bias-causing deviations.*

**Domain 3 – Bias due to missing outcome data**

| **Signalling question** | **Judgment** | **Reasoning** |
| --- | --- | --- |
| **3.1** Were outcome data available for nearly all participants? | **N** | Primary-outcome data for 1 149/1 273 (90.3%). |
| **3.2** Evidence that results were not biased by missing data? | **PY** | Authors used **multiple imputation by chained equations** for all missing BPD data (excluding deaths) and pre-specified this approach. |
| 3.3 & 3.4 | Not triggered | 3.2 = PY (adequate handling). |

**Domain 3 judgment:** **Low risk**
*< 95 % complete, but prespecified multiple-imputation strategy makes bias unlikely.*

**Domain 4 – Bias in measurement of the outcome**

| **Signalling question** | **Judgment** | **Reasoning** |
| --- | --- | --- |
| **4.1** Was outcome method inappropriate? | **N** | BPD defined physiologically via standardized oxygen-saturation test or need for support—accepted clinical standard. |
| **4.2** Could measurement differ between groups? | **N** | Same protocol applied to all infants; no indication of group-specific procedures. |
| **4.3** Were assessors aware of intervention? | **N** | Outcome assessors were among the blinded clinical/research personnel. |

**Domain 4 judgment:** **Low risk**
*Validated, objective measurement with blinded assessors for both groups.*

**Domain 5 – Bias in selection of the reported result**

| **Signalling question** | **Judgment** | **Reasoning** |
| --- | --- | --- |
| **5.1** Was the analysis pre-specified? | **Y** | Prespecified statistical analysis plan referenced. |
| **5.2** Result selected from multiple outcome measurements? | **N** | Single primary outcome/time-point defined prospectively; no alternate measures reported. |
| **5.3** Result selected from multiple analyses? | **N** | Only the planned ITT model (with prespecified covariates) reported; no evidence of selective modelling. |

**Domain 5 judgment:** **Low risk**

**Overall risk-of-bias judgement**

**Overall:** **Low risk of bias**
**Justification:** All five domains were rated *Low risk*; therefore the trial’s primary-outcome estimate is judged to be at low risk of bias under RoB 2.0.

## Study 25

**Primary outcome assessed: Brachial-artery flow-mediated dilation (FMD)**

**DOMAIN 1 Risk of bias arising from the randomization process**

| **Signalling question** | **Code** | **Reasoning (direct quotes)** |
| --- | --- | --- |
| 1.1 Was the allocation sequence random? | **PY** | “Participants were **randomly assigned** to either a control or a RT group. Randomization was **stratified and balanced by age range … and baseline body fat percentage**” . No method of sequence generation is reported, but the language implies purposeful randomisation. |
| 1.2 Was the allocation sequence concealed until participants were enrolled and assigned? | **PN** | The article gives **no description of any concealment mechanism** (central service, sealed envelopes, etc.); concealment cannot be verified. |
| 1.3 Did baseline differences suggest a problem with randomisation? | **N** | Baseline Table 1 shows similar age, BMI, fat mass, blood pressure, etc., with **all P > 0.10** . |

**Domain 1 Judgment: High risk**
*Because allocation concealment is not described (1.2 = PN), the algorithm classifies the domain as High risk despite balanced baseline characteristics.*

**DOMAIN 2 Risk of bias due to deviations from intended interventions (effect of assignment)**

| **Signalling question** | **Code** | **Reasoning** |
| --- | --- | --- |
| 2.1 Were participants aware of their assigned intervention? | **Y** | RT participants attended supervised resistance-training sessions; controls received only educational brochures—blinding was impossible . |
| 2.2 Were carers / trial personnel aware of intervention? | **Y** | Fitness specialists supervised RT sessions, so personnel necessarily knew the allocation . |
| 2.3 Were there deviations from intended intervention that arose because of the trial context? | **N** | No cross-overs or compensatory behaviours are reported; RT adherence averaged **90 % (93 ± 3 of 104 sessions)** and controls remained non-training . |
| 2.6 Was an appropriate analysis used to estimate the effect of assignment? | **Y** | All **30 randomised women (15 + 15)** were “studied before and after a 1-yr RT intervention” with repeated-measures ANOVA; no exclusions are noted . |

*Part 1 = Low risk (2.1/2.2 = Y but 2.3 = N); Part 2 = Low risk (2.6 = Y).*

**Domain 2 Judgment: Low risk**
*Open-label design did not lead to protocol deviations, and all participants were analysed.*

**DOMAIN 3 Risk of bias due to missing outcome data**

| **Signalling question** | **Code** | **Reasoning** |
| --- | --- | --- |
| 3.1 Were outcome data available for nearly all participants? | **Y** | The flow diagram implicit in the text indicates **all 30 women provided pre- and post-intervention FMD data** (no losses reported) . |

**Domain 3 Judgment: Low risk**
*Complete outcome data (> 95 %) were available; no further questions triggered.*

**DOMAIN 4 Risk of bias in measurement of the outcome**

| **Signalling question** | **Code** | **Reasoning** |
| --- | --- | --- |
| 4.1 Was the method of measuring the outcome inappropriate? | **N** | FMD was assessed with established ultrasound methodology previously validated in the laboratory . |
| 4.2 Could measurement differ between groups? | **N** | The identical protocol and equipment were used for both groups . |
| 4.3 Were outcome assessors aware of intervention? | **N** | “Digital image analysis was performed by the same trained reader, **who was blinded to group assignments**.” |

**Domain 4 Judgment: Low risk**
*Validated, standardised outcome measurement with blinded assessor.*

**DOMAIN 5 Risk of bias in selection of the reported result**

| **Signalling question** | **Code** | **Reasoning** |
| --- | --- | --- |
| 5.2 Result selected from multiple outcome measurements? | **PN** | FMD was the only endothelial-function measure designated and both peak FMD and AUC were reported; selective reporting is unlikely. |
| 5.3 Result selected from multiple eligible analyses? | **PN** | A single, standard repeated-measures ANOVA is presented; no alternative analyses are hinted at. |
| 5.1 Was the analysis plan pre-specified? | **PY** | No protocol/SAP is cited, but the analysis aligns with stated objectives. |

*Because 5.2 and 5.3 are not affirmative and 5.1 = PY, the algorithm assigns Low risk.*

**Domain 5 Judgment: Low risk**

**Overall risk-of-bias judgment: High risk**

*The trial is at High risk of bias because Domain 1 (randomisation process) is High risk owing to unreported allocation-concealment procedures. All other domains were Low risk.*

## Study 26

**Filled RoB 2.0 assessment**

**Study:** de Jonghe et al., 2014 — Melatonin vs placebo for preventing delirium after hip-fracture surgery
 **Primary outcome:** Incidence of delirium within the first 8 days of admission.

**DOMAIN 1 – Bias arising from the randomisation process**

| **Signalling question** | **Response** | **Reasoning** |
| --- | --- | --- |
| **1.1 Was the allocation sequence random?** | **Y** | “Randomisation was stratified by study centre, with fixed blocks of 10 patients… an independent statistician generated a randomisation schedule.” |
| **1.2 Was the allocation sequence concealed until participants were enrolled and assigned?** | **Y** | “The trial pharmacist maintained the randomisation list… investigators, other staff members and patients remained blinded until after the last patient had completed the study.” |
| **1.3 Did baseline differences suggest a problem with the randomisation process?** | **N** | Baseline table shows groups well balanced; only minor differences (benzodiazepine use, prior delirium) that were adjusted for. |

**Domain 1 judgment:** **Low risk** – sequence was random and concealed; no important baseline imbalances.

**DOMAIN 2 – Bias due to deviations from intended interventions (effect of assignment)**

| **Signalling question** | **Response** | **Reasoning** |
| --- | --- | --- |
| **2.1 Were participants aware of their assigned intervention?** | **N** | Double-blind trial; participants remained blinded. |
| **2.2 Were carers and trial personnel aware?** | **N** | Staff and investigators blinded until data lock. |
| **2.3 Were there deviations from intended interventions that arose because of the experimental context?** | **N** | No deviations reported; identical-appearing tablets dispensed by pharmacy. |
| **2.6 Was an appropriate analysis used to estimate the effect of assignment?** | **PN** | Authors stated intention-to-treat, yet excluded 66/444 (15 %) after randomisation (withdrawals, deaths, baseline delirium), giving a modified ITT. |
| **2.7 Was there potential for substantial bias due to inappropriate analysis?** | **PN** | Exclusions reduce precision, but direction of effect unlikely to change; authors discuss loss of power rather than bias. |

**Domain 2 judgment:** **Some concerns** – blinding adequate, but modified ITT introduces some analytical concern.

**DOMAIN 3 – Bias due to missing outcome data**

| **Signalling question** | **Response** | **Reasoning** |
| --- | --- | --- |
| **3.1 Were data for this outcome available for all or nearly all participants randomised?** | **N** | Primary-outcome data missing for 66/444 (14.9 %). |
| **3.2 Is there evidence that the result was not biased by missing outcome data?** | **PN** | No sensitivity analyses to explore impact of missing cases. |
| **3.3 Could missingness in the outcome depend on its true value?** | **PY** | Exclusions included early delirium, deaths and transfers—events plausibly related to outcome. |
| **3.4 Did the authors model or adjust for missing data?** | **N** | No imputation or modelling reported. |

**Domain 3 judgment:** **High risk** – substantial, outcome-related missing data with no adjustment.

**DOMAIN 4 – Bias in measurement of the outcome**

| **Signalling question** | **Response** | **Reasoning** |
| --- | --- | --- |
| **4.1 Was the method of measuring the outcome appropriate?** | **Y** | Delirium diagnosed daily using DSM-IV criteria plus Delirium Observation Screening Scale. |
| **4.2 Could measurement of the outcome have differed between groups?** | **N** | Standardised, blinded assessments across groups. |
| **4.3 Were outcome assessors aware of the intervention received?** | **N** | Assessors remained blinded until study completion. |

**Domain 4 judgment:** **Low risk**

**DOMAIN 5 – Bias in selection of the reported result**

| **Signalling question** | **Response** | **Reasoning** |
| --- | --- | --- |
| **5.1 Were data analysed in accordance with a prespecified plan?** | **PY** | Trial registered (NTR1576) but no public statistical analysis plan provided. |
| **5.2 Were multiple eligible outcome measurements within the outcome domain?** | **N** | Only a single definition of incident delirium reported. |
| **5.3 Were multiple eligible analyses of the data conducted?** | **N** | Only primary and predefined adjusted analyses presented. |

**Domain 5 judgment:** **Some concerns**

**OVERALL RISK-OF-BIAS JUDGEMENT: High risk**

Because Domain 3 is at High risk, the overall risk of bias for the primary outcome is **High** according to RoB 2 rules.

## Study 27

**PRIMARY OUTCOME ASSESSED:** cumulative incidence of IgE-mediated allergic disease (eczema, asthma, allergic rhinitis or food allergy) during the first 3 years of life.

**DOMAIN 1 — Bias arising from the randomization process**

| **Signalling question** | **Judgement** | **Reasoning** |
| --- | --- | --- |
| 1.1 Was the allocation sequence random? | **PN** | The paper states that children “were **randomly allocated** to receive fish-oil or control capsules” with no description of sequence generation . |
| 1.2 Was the allocation sequence concealed until participants were enrolled and assigned? | **NI** | No methods describing central randomization, sealed envelopes, etc., are reported. |
| 1.3 Did baseline differences suggest a problem with the randomization process? | **NI** | Baseline demographics are said to have been “previously reported,” and no table of baseline characteristics appears in this article . |

**Domain 1 judgement: Some concerns** *Allocation concealment not described (1.2 = NI). Without that information, even though no imbalances are evident, the algorithm yields “Some concerns.”*

**DOMAIN 2 — Bias due to deviations from intended interventions (effect of assignment)**

| **Signalling question** | **Judgement** | **Reasoning** |
| --- | --- | --- |
| 2.1 Were participants aware of their assignment? | **N** | The trial was “**double-blinded**; all capsules were similar in size, shape and colour” . |
| 2.2 Were carers or trial personnel aware? | **N** | Same double-blind statement applies . |
| 2.3–2.5 Deviations because of awareness? | — | Skipped (2.1 & 2.2 = N ⇒ Part 1 = Low risk). |
| 2.6 Was an appropriate analysis used (ITT)? | **Y** | “**All analyses were performed according to the intention-to-treat principle**” . |
| 2.7 Potential for substantial bias due to analysis? | — | Skipped (2.6 = Y). |

**Domain 2 judgement: Low risk** *Double-blinding prevented performance deviations; ITT analysis appropriate.*

**DOMAIN 3 — Bias due to missing outcome data**

| **Signalling question** | **Judgement** | **Reasoning** |
| --- | --- | --- |
| 3.1 Were outcome data available for nearly all participants? | **N** | 638 / 706 children (≈ 90 %) attended the 3-y visit; similar loss at 1 y . |
| 3.2 Evidence that results were not biased by missing data? | **Y** | Authors **used multiple imputation (50 datasets)** and state the missing-at-random assumption was reasonable . |
| 3.3 Could missingness depend on true outcome? | — | Skipped (3.2 = Y). |

**Domain 3 judgement: Low risk** *Although attrition exceeded 5 %, comprehensive multiple-imputation ITT analysis and explicit discussion of missing-data assumptions mitigate bias.*

**DOMAIN 4 — Bias in measurement of the outcome**

| **Signalling question** | **Judgement** | **Reasoning** |
| --- | --- | --- |
| 4.1 Was the method of measuring the outcome inappropriate? | **N** | Outcomes were diagnosed at standardized medical assessments plus skin-prick testing . |
| 4.2 Could measurement differ between groups? | **N** | Same diagnostic procedures used for all children. |
| 4.3 Were outcome assessors aware of assignment? | **N** | “**All were blinded to treatment group allocation**” (physicians and research nurses) . |

**Domain 4 judgement: Low risk** *Validated, objective methods; blinded assessors; uniform procedures.*

**DOMAIN 5 — Bias in selection of the reported result**

| **Signalling question** | **Judgement** | **Reasoning** |
| --- | --- | --- |
| 5.2 Result selected from multiple outcome measurements? | **NI** | The paper reports several allergic outcomes but does not describe how time-points/definitions were chosen. |
| 5.3 Result selected from multiple analyses? | **PN** | Both adjusted and unadjusted analyses are shown; no unreported alternative analyses suggested. |
| 5.1 Was the analysis plan pre-specified? | **NI** | Trial and follow-up are registered (ACTRN numbers given) , but no dated SAP is provided. |

**Domain 5 judgement: Some concerns** *Lack of publicly available pre-specified analysis plan and unclear multiplicity leave some room for selective reporting.*

***OVERALL RISK-OF-BIAS JUDGEMENT: Some concerns***

*Domain 1 (randomization) and Domain 5 (reporting) both carry “Some concerns,” while Domains 2–4 are low risk; no domain is high risk.*

## Study 28

**Primary outcome assessed:** Knee Society *total* score at the final (mean 13.2 y) follow-up.

**DOMAIN 1 – *Bias arising from the randomization process***

| **Signalling question** | **Answer** | **Direct quote / rationale** |
| --- | --- | --- |
| **1.1 Was the allocation sequence random?** | NI | The study is described only as “*prospective, randomized*” with no details of sequence generation . |
| **1.2 Was the allocation sequence concealed until participants were enrolled and assigned?** | NI | The article gives **no information** on any concealment mechanism. |
| **1.3 Did baseline differences suggest a problem with randomization?** | N | Pre-operative Knee Society totals were virtually identical (25 ± 8 vs 26 ± 9; *p* = 0.731) , and no other imbalances are reported. |

**Domain 1 judgement: Some concerns** *Allocation concealment is unreported (1.2 = NI). With balanced baselines (1.3 = N) the algorithm yields “Some concerns”.*

**DOMAIN 2 – *Bias due to deviations from intended interventions (effect of assignment)***

| **Signalling question** | **Answer** | **Direct quote / rationale** |
| --- | --- | --- |
| **2.1 Were participants aware of their assigned intervention?** | NI | Blinding of patients is never mentioned. |
| **2.2 Were carers/personnel aware?** | NI | No statement on surgeon or assessor blinding to insert type. |
| **2.3 Were there deviations from the intended intervention arising because of the trial context?** | NI | No deviations or adherence data described. |
| **2.6 Was an appropriate analysis used to estimate the effect of assignment?** | NI | Follow-up cohort shrank from 319 to 300 patients; the authors analysed only those seen at ≥12 y, without specifying ITT: “*300 patients (600 knees) were included in the present follow-up study*” . |
| **2.7 Was there potential for substantial bias due to inappropriate analysis?** | Y | Excluding 19/319 patients (6 %) who were lost or died before 12 y could bias functional outcomes, and no sensitivity analyses are given. |

*Part 1* (questions 2.1–2.5): **Some concerns** (insufficient information).
 *Part 2* (analysis): **High risk** (2.6 = NI → 2.7 = Y).

**Domain 2 judgement: High risk** *Lack of information on blinding/deviations plus a non-ITT analysis likely to bias results.*

**DOMAIN 3 – *Bias due to missing outcome data***

| **Signalling question** | **Answer** | **Direct quote / rationale** |
| --- | --- | --- |
| **3.1 Were outcome data available for almost all participants?** | N | 300/319 patients (94 %) provided 13-y data; nine lost before 5 y and eight lost/died before 12 y . |
| **3.2 Evidence that results were not biased by missing data?** | N | No sensitivity analyses or comparisons of characteristics of those lost. |
| **3.3 Could missingness depend on the true outcome?** | Y | Deaths and losses after major surgery plausibly relate to health/function. |
| **3.4 Is it likely?** | NI | Reasons not provided. |

**Domain 3 judgement: High risk** *(3.1 = N, 3.2 = N, 3.3 = Y, 3.4 = NI → High risk by algorithm).*

**DOMAIN 4 – *Bias in measurement of the outcome***

| **Signalling question** | **Answer** | **Direct quote / rationale** |
| --- | --- | --- |
| **4.1 Was the outcome measurement method inappropriate?** | N | Knee Society score is a validated instrument . |
| **4.2 Could measurement differ between groups?** | N | Both groups assessed with the same scale at identical visits. |
| **4.3 Were outcome assessors aware of intervention?** | NI | Blinding not stated for clinical scorers; only radiograph reader is described (“*One observer, who had not been involved in the surgical procedure, assessed the radiographs*”) . |
| **4.4 Could awareness influence assessment?** | Y | Knee Society score involves subjective components (pain, stair-climbing). |
| **4.5 Is it likely that assessment was influenced?** | PY | Same surgeon centre; plausible but not certain. |

**Domain 4 judgement: High risk** *(Assessor awareness unclear + subjective outcome; 4.3 = NI, 4.4 = Y, 4.5 = PY → High risk).*

**DOMAIN 5 – *Bias in selection of the reported result***

| **Signalling question** | **Answer** | **Direct quote / rationale** |
| --- | --- | --- |
| **5.2 Result selected from multiple outcome measurements?** | Y | Numerous outcomes (Knee Society, WOMAC, VAS, survival) at multiple time-points; only 13-y results highlighted, without explaining selection. |
| **5.3 Result selected from multiple eligible analyses?** | PY | Bilateral-knee data could be analysed per-patient or per-knee; only per-knee shown. |
| (5.1 not reached) |  |  |

**Domain 5 judgement: High risk** *(5.2 = Y triggers High risk).*

**Overall risk-of-bias judgement: High risk**

*At least one domain (2, 3, 4, 5) is at High risk; therefore the overall study risk of bias for the Knee Society score outcome is* ***High****.*

## Study 29

**Randomized Controlled Trial evaluated** Gazal G, Mackie IC. “A comparison of paracetamol, ibuprofen or their combination for pain relief following extractions in children under general anaesthesia.” *Int J Paediatric Dentistry* 2007;17:169-177. Primary outcome assessed here: **post-extraction pain at 15 min, measured with the CHEOPS scale**.

**DOMAIN 1 – Bias arising from the randomization process**

| **Signalling question** | **Answer** | **Reasoning** |
| --- | --- | --- |
| 1.1 Was the allocation sequence random? | **Y** | “Using **computer-generated random numbers**, slips of paper were labelled … and placed in sequentially numbered envelopes.” |
| 1.2 Was the allocation sequence concealed until participants were enrolled & assigned? | **PY** | Envelopes were *“sequentially numbered”* and opened only after eligibility, by a dentist not involved in the study, *“ensur[ing] that both the patients and investigator were blinded.”* Opaqueness or sealing was not reported. |
| 1.3 Did baseline differences suggest a problem with randomization? | **N** | Authors state baseline sex, age, weight, number of extractions and timing **“were generally similar… There was no significant statistical difference on any of the assessment parameters.”** |

**Domain 1 judgment: Low risk** *Allocation was probably concealed (1.2 = PY) and clearly random (1.1 = Y), with balanced baseline characteristics (1.3 = N) → Low risk.*

**DOMAIN 2 – Bias due to deviations from intended interventions (effect of assignment)**

| **Signalling question** | **Answer** | **Reasoning** |
| --- | --- | --- |
| 2.1 Were participants aware of their assignment? | **N** | Study states blinding of “patients” to group allocation. |
| 2.2 Were carers/personnel aware? | **N** | The envelopes were opened by a dentist not otherwise involved; investigators were blinded. |
| — *Because 2.1 = 2.2 = N, Part 1 = Low risk (skip 2.3-2.5)* |  |  |
| 2.6 Was an appropriate analysis used to estimate effect of assignment? | **PN** | Eleven children excluded *after* randomization; analysis included only 201/212 (94.8 %). No ITT mentioned. |
| 2.7 Potential for substantial bias from analysis? | **PN** | Exclusions small and unrelated to analgesic effectiveness (medical unsuitability), so large bias unlikely. |

**Domain 2 judgment: Some concerns** *Part 1 = Low risk; Part 2 has PN for ITT (2.6) but PN for substantial bias (2.7) → Some concerns.*

**DOMAIN 3 – Bias due to missing outcome data**

| **Signalling question** | **Answer** | **Reasoning** |
| --- | --- | --- |
| 3.1 Were outcome data available for nearly all participants? | **PN** | 201/212 (94.8 %) provided pain data – slightly below the 95 % threshold. |
| 3.2 Evidence that results not biased by missing data? | **N** | No sensitivity analyses or justification reported. |
| 3.3 Could missingness depend on true outcome value? | **N** | Exclusions were for medical reasons before GA (heart disease, colds, overweight, anxiety) – unlikely linked to postoperative pain. |

**Domain 3 judgment: Low risk** *Although just under 95 % complete, missingness was small and clearly unrelated to pain outcomes (3.3 = N) → Low risk.*

**DOMAIN 4 – Bias in measurement of the outcome**

| **Signalling question** | **Answer** | **Reasoning** |
| --- | --- | --- |
| 4.1 Was the method of measuring the outcome inappropriate? | **N** | Pain measured with the validated CHEOPS scale; distress with a published five-face scale. |
| 4.2 Could measurement differ between groups? | **N** | “One researcher … made all the observations” using identical procedures for every child. |
| 4.3 Were outcome assessors aware of intervention? | **NI** | Assessor independence noted, but blinding to group not explicitly stated. |
| 4.4 Could assessor awareness have influenced measurement? | **Y** | Pain and distress are subjective; knowledge of treatment could influence scoring. |
| 4.5 Was such influence likely? | **PY** | If unblinded, a single assessor could (consciously or not) score groups differently. |

**Domain 4 judgment: High risk** *Assessor blinding is unreported (4.3 = NI); with subjective outcomes (4.4 = Y) and plausible influence (4.5 = PY) → High risk.*

**DOMAIN 5 – Bias in selection of the reported result**

| **Signalling question** | **Answer** | **Reasoning** |
| --- | --- | --- |
| 5.2 Result likely selected from multiple outcome measurements? | **PN** | Only distress and CHEOPS pain at two prespecified time-points were measured and reported. |
| 5.3 Result likely selected from multiple analyses? | **PN** | Standard parametric tests used; no evidence of extensive alternative modelling. |
| 5.1 Was the analysis plan pre-specified? | **NI** | No protocol or SAP cited. |

**Domain 5 judgment: Some concerns** *With PN for 5.2 & 5.3 but no prespecified plan (5.1 = NI) → Some concerns.*

**OVERALL RISK-OF-BIAS JUDGMENT**

**Overall: High risk of bias** *Domains 1 & 3 = Low risk; Domain 2 & 5 = Some concerns; Domain 4 = High risk → overall High risk of bias, chiefly driven by unclear assessor blinding with subjective outcomes.*

## Study 30

**Revised Cochrane Risk of Bias (RoB 2) – Primary outcome: incidence of moderate-to-severe interstitial fibrosis/tubular atrophy (IFTA) at 3 years**

**DOMAIN 1 – Bias arising from the randomization process**

| **Signalling question** | **Answer** | **Reasoning** |
| --- | --- | --- |
| 1.1 Was the allocation sequence random? | **Y** | “Patients were randomized … using a **random-number table**” |
| 1.2 Was the allocation sequence concealed until participants were enrolled and assigned? | **Y** | “**Sequentially numbered sealed envelopes** were used for allocation concealment.” |
| 1.3 Did baseline differences suggest a problem with the randomization process? | **PY** | An imbalance existed: basiliximab induction 43 % vs 24 % (P = 0.039) ; authors note “a lower quality of donors in the valganciclovir group” . |

**Domain 1 judgment:** *Some concerns* *Justification:* Allocation sequence was random and concealed (1.1 = Y, 1.2 = Y). A statistically significant baseline imbalance raises **probable** concern (1.3 = PY) → Some concerns.

**DOMAIN 2 – Bias due to deviations from intended interventions (effect of assignment)**

| **Signalling question** | **Answer** | **Reasoning** |
| --- | --- | --- |
| 2.1 Were participants aware of their assigned intervention? | **Y** | “parallel group, **open-label**, randomized controlled trial” |
| 2.2 Were carers or trial personnel aware of intervention assignments? | **Y** | Same quote as 2.1 (open-label). |
| 2.3 Were there deviations from intended intervention due to the trial context? | **NI** | No deviations or cross-overs are described; the report is silent on this point. |
| 2.4 If Y/PY to 2.3: Were these deviations likely to affect the outcome? | — | Not triggered (2.3 = NI). |
| 2.5 Were these deviations balanced between groups? | — | Not triggered. |
| 2.6 Was an appropriate analysis used (ITT)? | **Y** | “Data were analyzed according to the **intention-to-treat principle**.” |
| 2.7 If N/PN/NI to 2.6: Potential for substantial bias? | — | Not triggered. |

**Domain 2 judgment:** *Some concerns* *Justification:* Because the trial was open-label and deviation information is **not reported** (2.3 = NI) → Part 1 = Some concerns. ITT analysis used (Part 2 = Low risk). Combined → Some concerns.

**DOMAIN 3 – Bias due to missing outcome data**

| **Signalling question** | **Answer** | **Reasoning** |
| --- | --- | --- |
| 3.1 Were outcome data available for nearly all participants? | **N** | 119 randomized, 101 biopsied ⇒ 85 % observed (primary outcome). |
| 3.2 Evidence that results were not biased by missing data? | **N** | No sensitivity analysis or discussion of missing-data impact is reported. |
| 3.3 Could missingness depend on true outcome value? | **PY** | Main reasons for missing biopsy were **death or graft loss** , events plausibly linked to more severe graft pathology. |
| 3.4 Is it likely that it did? | **PY** | Because biopsy could not be performed in those with graft loss/death, it is *probably* associated with worse fibrosis. |

**Domain 3 judgment:** *High risk* *Justification:* <95 % data (3.1 = N) → no mitigating analysis (3.2 = N) → missingness probably related to outcome (3.3 = PY, 3.4 = PY) → High risk.

**DOMAIN 4 – Bias in measurement of the outcome**

| **Signalling question** | **Answer** | **Reasoning** |
| --- | --- | --- |
| 4.1 Was the method of measuring the outcome inappropriate? | **N** | Banff-classified histology on protocol biopsy is standard. |
| 4.2 Could measurement differ between groups? | **N** | Same biopsy procedures for both groups . |
| 4.3 Were outcome assessors aware of intervention? | **N** | “biopsies were evaluated … by a single pathologist **blinded to the study group**” |

**Domain 4 judgment:** *Low risk* *Justification:* Appropriate, blinded, identical measurement across groups (4.1 = N, 4.2 = N, 4.3 = N).

**DOMAIN 5 – Bias in selection of the reported result**

| **Signalling question** | **Answer** | **Reasoning** |
| --- | --- | --- |
| 5.2 Result selected from multiple outcome measurements? | **PN** | Only one definition/time-point (36 month biopsy) reported for the primary outcome. |
| 5.3 Result selected from multiple eligible analyses? | **PN** | Primary analysis pre-specified as ITT; no evidence of multiple alternative models. |
| 5.1 Was the analysis plan pre-specified? | **PY** | Trial was prospectively registered (ACTRN12610000016033) but protocol/SAP not published. |

**Domain 5 judgment:** *Low risk* *Justification:* No signs of selective measurement/analysis (5.2 = PN, 5.3 = PN) and analysis appears pre-specified (5.1 = PY) → Low risk.

**Overall risk-of-bias judgment: High risk**

*Because Domain 3 showed high risk from missing outcome data, the trial’s overall risk of bias is judged* ***high****.*

## Study 31

**DOMAIN 1 – Bias arising from the randomisation process**

| **Signalling question** | **Answer** | **Reasoning (direct quotation)** |
| --- | --- | --- |
| **1.1 Sequence random?** | **Y** | “*Randomization … was performed … using a randomization list generated by randomizer.org.*” |
| **1.2 Sequence concealed?** | **PY** | “*The blinding of the study was carried out by homologating the tablets and the packaging. The packages were identified by kit number; both the patient and the principal investigator were unaware of the allocation of participants.*” – concealment strongly implied but exact mechanism not detailed |
| **1.3 Baseline imbalance?** | **N** | Table 1 shows groups similar for age, sex, BMI and comorbidities; only a small difference in baseline O₂-saturation (89 % vs 93 %) which authors do not deem clinically important |

**Domain 1 judgement: Low risk** *Concealment probably adequate (1.2 = PY); sequence random (1.1 = Y); no meaningful baseline imbalances (1.3 = N) → Low risk.*

**DOMAIN 2 – Bias due to deviations from intended interventions (effect of assignment)**

| **Signalling question** | **Answer** | **Reasoning** |
| --- | --- | --- |
| **2.1 Participants aware?** | **N** | Trial described as “*double-blind*” |
| **2.2 Carers aware?** | **N** | Same double-blind statement. |
| *Because 2.1 = 2.2 = N → Part 1 = Low risk (skip 2.3–2.5).* |  |  |
| **2.6 Appropriate analysis?** | **Y** | Analyses performed in “*all treated patients (ATP), a group of* ***all the randomised patients*** *that received at least one dose …*” – every randomised participant received treatment, so ATP = ITT |
| *Part 2 therefore Low risk.* |  |  |

**Domain 2 judgement: Low risk** *Both blinding and ITT analysis adequate.*

**DOMAIN 3 – Bias due to missing outcome data**

| **Signalling question** | **Answer** | **Reasoning** |
| --- | --- | --- |
| **3.1 Outcome data for almost all?** | **Y** | Flow-chart states “*No patients were excluded from the statistical analysis since the data obtained was sufficient to be included in the final analysis.*” |

**Domain 3 judgement: Low risk** *All 20 randomised participants contributed outcome data.*

**DOMAIN 4 – Bias in measurement of the outcome**

| **Signalling question** | **Answer** | **Reasoning** |
| --- | --- | --- |
| **4.1 Outcome method inappropriate?** | **N** | Viral load quantified by standard RT-qPCR with synthetic RNA standard curve |
| **4.2 Measurement differ between groups?** | **N** | Same RT-qPCR protocol applied to all samples. |
| **4.3 Assessors aware of assignment?** | **NI** | Laboratory blinding not stated. |
| **4.4 Could awareness influence measurement?** | **N** | RT-qPCR provides objective copy numbers; assessor influence unlikely. |

**Domain 4 judgement: Low risk** *Validated, objective assay; even if lab staff were unblinded, it would not affect the machine-read result.*

**DOMAIN 5 – Bias in selection of the reported result**

| **Signalling question** | **Answer** | **Reasoning** |
| --- | --- | --- |
| **5.2 Result selected from multiple measurements?** | **PN** | Days 0, 2, 5, 7, 9 and “end of study” time-points were **pre-specified in the Methods** |
| **5.3 Result selected from multiple analyses?** | **PN** | Only one analysis strategy (group comparison with t-test / Fisher) is presented; no alternative models hinted. |
| **5.1 Analysis pre-specified?** | **NI** | Trial registered (NCT04625985) but protocol/SAP not provided |

**Domain 5 judgement: Some concerns** *Pre-specified time-points reduce selective-reporting risk (5.2 = PN), but lack of publicly available SAP leaves some residual uncertainty.*

**OVERALL RISK-OF-BIAS JUDGEMENT: Some concerns**

*Domains 1–4 are Low risk; Domain 5 shows Some concerns because the statistical analysis plan was not visible.*

## Study 32

**Target outcome assessed:** *Body-weight change at 14 weeks (primary outcome)*

**DOMAIN 1 – Bias arising from the randomization process**

| **Signalling Q** | **Judgment** | **Justification (direct quotes)** |
| --- | --- | --- |
| **1.1 Was the allocation sequence random?** | **Y** | “Randomization was performed by the statistician … using block sizes of 2 in the software program R” |
| **1.2 Was the allocation sequence concealed until participants were enrolled and assigned?** | **PN** | The article gives **no description of any central service, opaque envelopes, or other concealment method**; concealment is entirely unreported. |
| **1.3 Did baseline differences suggest a problem with the randomization?** | **N** | Baseline Table 1 shows very similar age, sex and BMI distributions across groups (e.g., BMI 39.2 ± 6.8 vs 40.1 ± 6.6) |

**Domain-level judgment:** **High risk** **Logic:** 1.2 = PN ⇒ Domain 1 = High risk (algorithm step 1).

**DOMAIN 2 – Bias due to deviations from intended interventions (effect of assignment)**

| **Signalling Q** | **Judgment** | **Justification** |
| --- | --- | --- |
| **2.1 Were participants aware of their assignment?** | **Y** | Behavioural diet trial; no blinding stated. “Participants were instructed to follow their assigned eating schedule…” |
| **2.2 Were carers/personnel aware?** | **Y** | Dietitians delivered eating-window counselling; blinding impossible and not claimed. |
| **2.3 Did awareness lead to deviations?** | **N** | No protocol-deviating co-interventions are reported; lower adherence in eTRE is *part of* the intervention, not caused by awareness. |
| **2.6 Was an appropriate analysis used?** | **Y** | “All analyses were intention-to-treat” |

*Part 1 = Low risk (2.3 = N); Part 2 = Low risk (2.6 = Y).*

**Domain-level judgment:** **Low risk**

**DOMAIN 3 – Bias due to missing outcome data**

| **Signalling Q** | **Judgment** | **Justification** |
| --- | --- | --- |
| **3.1 Were outcome data available for almost all?** | **N** | Only 59/90 (66 %) completed all week-14 measurements |
| **3.2 Evidence that results not biased by missing data?** | **PY** | Authors used linear mixed models and “multiple imputation by chained equations” for missing values , and attrition was balanced (20 % vs 24 %). |
| **3.3 Could missingness depend on true value?** | **PN** | Drop-outs mainly COVID-related or logistical (Figure 1) , not obviously tied to weight outcome. |

**Domain-level judgment:** **Low risk** (3.2 = PY ⇒ Low risk per algorithm).

**DOMAIN 4 – Bias in measurement of the outcome**

| **Signalling Q** | **Judgment** | **Justification** |
| --- | --- | --- |
| **4.1 Was method of measuring outcome inappropriate?** | **N** | Weight measured with clinic scale every 2 weeks; standard objective method |
| **4.2 Could measurement differ between groups?** | **N** | Same clinic procedures for both arms. |
| **4.3 Were assessors aware of intervention?** | **Y** | Staff not reported as blinded. |
| **4.4 Could awareness influence assessment?** | **PN** | Body-weight reading is objective; unlikely to be influenced by knowledge of group. |

**Domain-level judgment:** **Low risk** (objective outcome; any assessor awareness unlikely to bias).

**DOMAIN 5 – Bias in selection of the reported result**

| **Signalling Q** | **Judgment** | **Justification** |
| --- | --- | --- |
| **5.2 Multiple outcome measurements?** | **N** | Body-weight change at 14 weeks was a pre-specified co-primary outcome. |
| **5.3 Multiple eligible analyses?** | **PN** | Only ITT analysis reported; SAP referenced. |
| **5.1 Analysis pre-specified?** | **PY** | “The trial protocol and statistical analysis plan appear in Supplement 2 and 3” |

**Domain-level judgment:** **Low risk** (5.2 = N & 5.3 = PN ⇒ check 5.1; 5.1 = PY → Low risk).

**OVERALL RISK-OF-BIAS JUDGMENT: High risk**

**Rationale:** Domain 1 rated High risk because allocation concealment was not reported; all other domains Low risk. A single High-risk domain makes the overall risk of bias High.

## Study 33

Below is a fully worked-through RoB 2 (version 22 Aug 2019) assessment for the **primary outcome (change in number of migraines per 30 days at month 10)** in Holroyd 2010 (BMJ 341:c4871).

**DOMAIN 1 – Bias arising from the randomisation process**

| **Signalling question** | **Judgement** | **Direct quote / reasoning** |
| --- | --- | --- |
| **1.1 Was the allocation sequence random?** | **Y** | “*A statistician … generated the randomisation sequence by* ***computer*** *…*” |
| **1.2 Was the allocation sequence concealed until participants were enrolled and assigned?** | **Y** | “…supplied **sealed opaque envelopes** … the site manager opened the next envelope in the sequence.” |
| **1.3 Did baseline differences suggest a problem with randomisation?** | **N** | Table 1 shows similar age, sex and migraine frequency across the four groups; authors do not report any significant imbalances. |

**Domain 1 judgement: Low risk** *1.2=Y and 1.1=Y → proceed to 1.3; 1.3=N → Low risk.*

**DOMAIN 2 – Bias due to deviations from intended interventions (effect of assignment)**

| **Signalling question** | **Judgement** | **Direct quote / reasoning** |
| --- | --- | --- |
| **2.1 Were participants aware of their assigned intervention?** | **PY** | Drug component double-blind, but “*Treatment conditions were blinded* ***only for the preventive drug*** *… not for the administration of behavioural migraine management.*” |
| **2.2 Were carers/trial personnel aware?** | **PY** | Behavioural therapists necessarily knew which patients received the behavioural programme. |
| **2.3 Were there deviations from intended intervention due to the trial context?** | **N** | High adherence to both drug (≈90 % of days) and behavioural homework (≈70 %) is reported; no cross-overs described. |
| **2.6 Was an appropriate analysis used to estimate the effect of assignment?** | **Y** | “*Efficacy analyses were* ***intention-to-treat*** *… included all randomised participants (n = 232).*” |

**Domain 2 judgement: Low risk** *Although blinding was partial (2.1/2.2 = PY), no deviations occurred (2.3=N) and ITT was used (2.6=Y) → Part 1 = Low risk, Part 2 = Low risk → Domain 2 Low risk.*

**DOMAIN 3 – Bias due to missing outcome data**

| **Signalling question** | **Judgement** | **Direct quote / reasoning** |
| --- | --- | --- |
| **3.1 Were outcome data available for nearly all participants?** | **N** | Attrition to month 10 was 35 % (81/232). |
| **3.2 Is there evidence that results were not biased by missing data?** | **Y** | Mixed-model repeated-measures (MMRM) with maximum-likelihood estimation and two sensitivity analyses were performed. |

**Domain 3 judgement: Low risk** *3.1=N but 3.2=Y → Low risk.*

**DOMAIN 4 – Bias in measurement of the outcome**

| **Signalling question** | **Judgement** | **Direct quote / reasoning** |
| --- | --- | --- |
| **4.1 Was the method of measuring the outcome inappropriate?** | **N** | Headache frequency recorded daily in a handheld electronic diary—validated method. |
| **4.2 Could measurement differ between groups?** | **N** | All groups used the same electronic diary; no indication of differential procedures. |
| **4.3 Were outcome assessors aware of intervention?** | **N** | Outcome derived automatically from participant-entered diaries; no separate assessor. |

**Domain 4 judgement: Low risk** *4.1=N and 4.2=N; assessor blinding not relevant → Low risk.*

**DOMAIN 5 – Bias in selection of the reported result**

| **Signalling question** | **Judgement** | **Direct quote / reasoning** |
| --- | --- | --- |
| **5.2 Result selected from multiple outcome measurements?** | **PN** | Only one definition (migraines/30 days) is reported for the primary outcome; no alternative scales/time-points suggested. |
| **5.3 Result selected from multiple eligible analyses?** | **PN** | A single ITT analysis with predefined covariates is presented; no unreported subgroup analyses mentioned. |
| **5.1 Was the analysis plan pre-specified?** | **NI** | The paper cites ClinicalTrials.gov registration (NCT00910689) but does not provide a dated SAP. |

**Domain 5 judgement: Some concerns** *5.2 & 5.3 = PN, but 5.1 = NI → Some concerns.*

**OVERALL RISK-OF-BIAS JUDGEMENT: Some concerns**

Domains 1–4 are Low risk; Domain 5 shows Some concerns due to lack of a public, dated statistical analysis plan, so overall the study carries **some concerns** about risk of bias but no domain at high risk.

## Study 34

**DOMAIN 1: Risk of Bias from the Randomization Process**

| **Signalling question** | **Answer** | **Quote‐based reasoning** |
| --- | --- | --- |
| **1.1 Was the allocation sequence random?** | **Y** | “subjects were randomised (**by table of random numbers**) in double-blind fashion” (Methods) |
| **1.2 Was the allocation sequence concealed until participants were enrolled and assigned?** | **NI** | The paper gives no description of central allocation, sealed envelopes, or any other concealment method. |
| **1.3 Did baseline differences suggest a problem with the randomization process?** | **NI** | Baseline Table 1 is presented only for the whole cohort; the authors do **not** report any between-group comparison. |

**Domain 1 Judgment: Some concerns** *Allocation was clearly random (1.1 = Y) but concealment is not described (1.2 = NI). With no group-specific baseline data (1.3 = NI) the algorithm yields “Some concerns”.*

**DOMAIN 2: Risk of Bias Due to Deviations from Intended Interventions (Effect of Assignment)**

| **Signalling question** | **Answer** | **Quote‐based reasoning** |
| --- | --- | --- |
| **2.1 Were participants aware of their assigned intervention?** | **PN** | Trial is described as “**double-blind**” and authors note “None of the subjects reported awareness of pacing” (Results). |
| **2.2 Were carers/personnel aware?** | **PN** | Also described as double-blind with no statement of staff unblinding. |
| *Because 2.1 & 2.2 are both N/PN → Part 1 Low risk. 2.3–2.5 not triggered.* |  |  |
| **2.6 Was an appropriate analysis used (ITT or equivalent)?** | **N** | Outcomes are reported for **25 of 34** randomised participants; nine drop-outs were excluded from the main analyses. |
| **2.7 Is there potential for substantial bias due to the analysis?** | **Y** | Excluding 26 % of participants (including deaths and cross-overs) could have materially biased effect estimates. No sensitivity/ITT analysis is reported. |

**Domain 2 Judgment: High risk** *Part 1 Low risk (blinding likely maintained). Part 2 High risk (per-protocol analysis with substantial attrition, 2.7 = Y). High-risk component → Domain 2 = High risk.*

**DOMAIN 3: Risk of Bias Due to Missing Outcome Data**

| **Signalling question** | **Answer** | **Quote‐based reasoning** |
| --- | --- | --- |
| **3.1 Were outcome data available for nearly all participants?** | **N** | Only **25/34** (74 %) supplied outcome data. |
| **3.2 Evidence that results were not biased by missing data?** | **N** | No imputation, no sensitivity analyses, and no discussion of missing-data impact. |
| **3.3 Could missingness depend on true outcome?** | **Y** | Drop-outs included **syncope**, **physician-directed cross-over**, deaths, and stroke—all plausibly related to fall-risk outcomes. |
| **3.4 Is it likely that it did?** | **Y** | The clinical reasons (syncope, serious events) strongly suggest outcome-dependent missingness. |

**Domain 3 Judgment: High risk** *3.1 = N → evaluate 3.2 (N) → 3.3 = Y → 3.4 = Y → High risk.*

**DOMAIN 4: Risk of Bias in Measurement of the Outcome**

| **Signalling question** | **Answer** | **Quote‐based reasoning** |
| --- | --- | --- |
| **4.1 Was the method of measuring the outcome inappropriate?** | **N** | Outcomes were captured with “**daily fall diaries returned at weekly intervals**”―a standard, validated approach in geriatric fall research. |
| **4.2 Could measurement differ between groups?** | **N** | All participants used identical diaries and mailing procedures irrespective of pacing mode. |
| **4.3 Were outcome assessors aware of intervention?** | **PN** | Diaries were self-completed; the trial was “double-blind”, so assessor awareness was *probably* absent, though details are sparse. |

**Domain 4 Judgment: Low risk** *4.1 = N, 4.2 = N, 4.3 = PN ⇒ assessor blinding probably adequate; algorithm → Low risk.*

**DOMAIN 5: Risk of Bias in Selection of the Reported Result**

| **Signalling question** | **Answer** | **Quote‐based reasoning** |
| --- | --- | --- |
| **5.2 Result likely selected from multiple outcome measurements?** | **PN** | Only one primary outcome (“number of falls”) was specified and reported; other potential fall metrics (e.g., injurious falls) were described as secondary. |
| **5.3 Result likely selected from multiple eligible analyses?** | **PN** | No subgroup or alternative model selection is mentioned, but the absence of a protocol leaves some uncertainty. |
| **5.1 Was the analysis pre-specified?** | **NI** | The article does not reference a protocol or SAP dated before data lock. |

**Domain 5 Judgment: Some concerns** *Both 5.2 & 5.3 are PN (not Y/PY). With 5.1 = NI the algorithm gives “Some concerns”.*

**Overall Risk-of-Bias Judgment: High risk**

*Domains 2 and 3 are High risk; therefore the trial is at overall high risk of bias despite low risk in outcome measurement.*

## Study 35

**DOMAIN 1 – Risk of bias arising from the randomization process**

1.1 Y – “...using a **computer-generated password-protected randomization scheme** in forced blocks of 20”
 1.2 PY – Separate research assistant assigned groups, but no explicit central/opaque method described
 1.3 Y – Control group had **more females, longer pain duration, and smaller mouth opening** (all P ≤ .03)
 **Domain 1 judgment: Some concerns** – Concealment probably adequate but baseline imbalances observed.

**DOMAIN 2 – Risk of bias due to deviations from intended interventions**

2.1 N – Participants were masked
 2.2 N – Injector/outcome assessor also masked; syringes prepared separately
 2.6 Y – Analysis *“performed by intention to treat.”* **Domain 2 judgment: Low risk**

**DOMAIN 3 – Risk of bias due to missing outcome data**

3.1 Y – Only **2 of 42 participants (4.8 %)** withdrew; outcomes carried forward
 **Domain 3 judgment: Low risk**

**DOMAIN 4 – Risk of bias in measurement of the outcome**

4.1 N – Primary outcome used validated 0-10 NRS; MIO measured with TheraBite device
 4.2 N – Same instruments used for both groups (no indication of differences)
 4.3 N – Assessors were masked along with participants
 **Domain 4 judgment: Low risk**

**DOMAIN 5 – Risk of bias in selection of the reported result**

5.2 PN – Single obvious way to measure primary outcome; multiple time-points reported consistently
 5.3 PN – Single ITT analysis; no unexplained subgroup/selective analyses
 5.1 PY – Trial registered (NCT01706172) but no dated SAP visible
 **Domain 5 judgment: Low risk**

**Overall risk-of-bias judgment: Some concerns**

Only Domain 1 raised “some concerns” (baseline imbalances & partial information on concealment); all other domains were judged low risk.

## Study 36

**Primary outcome evaluated:** Covid-19–related hospitalisation or death from any cause by day 29.

**Domain 1 – Bias arising from the randomisation process**

| **Signalling question** | **Response** | **Reasoning (direct evidence)** |
| --- | --- | --- |
| **1.1 Was the allocation sequence random?** | **PY** | Paper states patients were “*randomly assigned, in a 1:1 ratio*” to antibody or placebo, but the method of generating the sequence is not described . |
| **1.2 Was the allocation sequence concealed until participants were enrolled and assigned?** | **NI** | No information on central allocation, use of IWRS, opaque envelopes, etc., is provided in the main article. |
| **1.3 Did baseline differences suggest a problem with randomisation?** | **N** | Baseline characteristics (age, sex, BMI, comorbidities, symptom duration, viral-load Ct) are well balanced between groups (Table 1) . |

**Domain 1 Judgement:** **Some concerns** **Justification:** Allocation concealment not reported (1.2 = NI). With random sequence generation unclear (1.1 = PY) but balanced baselines (1.3 = N), the algorithm yields “Some concerns”.

**Domain 2 – Bias due to deviations from intended interventions**

*(Effect of assignment to intervention)*

| **Signalling question** | **Response** | **Reasoning** |
| --- | --- | --- |
| **2.1 Were participants aware of their assigned intervention?** | **N** | Trial described as *double-blind, placebo-controlled* . |
| **2.2 Were carers/trial personnel aware of the assignments?** | **N** | Same statement of double-blinding applies. |
| **2.6 Was an appropriate analysis used to estimate the effect of assignment?** | **PN** | Primary analysis included only the 1,035 participants who received an infusion; 14 randomised but not infused were excluded (Figure 1) , so analysis was modified-ITT rather than full ITT. |
| **2.7 If not, was there potential for substantial bias?** | **PN** | Exclusions were <2 % and balanced across arms; unlikely to materially change outcome. |

**Domain 2 Judgement:** **Some concerns** Analysis population excluded a small minority of randomised participants, introducing limited but non-negligible risk.

**Domain 3 – Bias due to missing outcome data**

| **Signalling question** | **Response** | **Reasoning** |
| --- | --- | --- |
| **3.1 Were data available for all, or nearly all, participants?** | **Y** | Only 3/1,035 participants (<0.3 %) were lost to follow-up (2 in active, 1 in placebo) before day 29 . |

*Sub-questions 3.2–3.4 not triggered.*

**Domain 3 Judgement:** **Low risk**

**Domain 4 – Bias in measurement of the outcome**

| **Signalling question** | **Response** | **Reasoning** |
| --- | --- | --- |
| **4.1 Was the method of measuring the outcome appropriate?** | **Y** | Hospitalisation ≥24 h or death are objective, clinically verified endpoints . |
| **4.2 Could measurement have differed between groups?** | **N** | Outcome ascertainment is routine and independent of allocation. |
| **4.3 Were outcome assessors aware of intervention?** | **N** | Double-blind design prevents assessor awareness . |

**Domain 4 Judgement:** **Low risk**

**Domain 5 – Bias in selection of the reported result**

| **Signalling question** | **Response** | **Reasoning** |
| --- | --- | --- |
| **5.1 Was the analysis plan pre-specified and available?** | **Y** | Statistical analysis plan and protocol are publicly posted at NEJM.org; trial registered (NCT04427501) . |
| **5.2 Were there multiple eligible outcome measurements?** | **N** | Primary endpoint clearly prespecified and uniquely defined. |
| **5.3 Were there multiple eligible analyses of the data?** | **N** | Single primary analysis presented; multiplicity controlled with prespecified family-wise procedure . |

**Domain 5 Judgement:** **Low risk**

**Overall Risk of Bias for the primary outcome: Some concerns**

The trial is well-conducted and blinded with minimal missing data and objective outcomes. Remaining issues stem from (i) lack of reported details on allocation concealment and sequence generation, and (ii) exclusion of 14 un-infused randomised participants from the primary analysis, yielding “Some concerns” overall according to RoB 2 rules.

## Study 37

**Proportion of participants with *Streptococcus mutans* ≥ 10⁵ CFU /ml at 6 months (T2)**

**Domain 1 – Bias arising from the randomisation process**

| **Signalling question** | **Answer** | **Reasoning** |
| --- | --- | --- |
| **1.1 Was the allocation sequence random?** | **PN** | Authors state that “patients … were **randomly assigned** to one of the treatment groups” but give **no description of the random‐sequence generation method** . |
| **1.2 Was the allocation sequence concealed until participants were enrolled and assigned?** | **NI** | No information on any concealment mechanism (e.g. central randomisation, sealed envelopes) is provided in the paper. |
| **1.3 Did baseline differences suggest a problem with randomisation?** | **N** | The Results section reports “**No difference was found in the demographics and oral hygiene indices between the two groups**” , and Table 1 shows closely similar baseline characteristics. |

**Domain-level judgement: Some concerns** *Allocation concealment not reported (1.2 = NI). Baseline balanced, but lack of concealment prevents a low-risk rating.*

**Domain 2 – Bias due to deviations from intended interventions (effect of assignment)**

**Part 1 – deviations**

| **Signalling question** | **Answer** | **Reasoning** |
| --- | --- | --- |
| **2.1 Were participants aware of their assigned intervention?** | **Y** | Orthodontic appliances (self-ligating vs conventional brackets) are visible; no blinding is claimed. |
| **2.2 Were carers/personnel aware of assignments?** | **PY** | The orthodontist “did not know the bracket group assignment at the time of the first saliva collection” , implying *initial* blinding only; after bonding the operator necessarily knows which bracket was placed. |
| **2.3 Were there deviations from intended intervention due to the trial context?** | **PN** | Paper does not mention any cross-overs or protocol deviations. |

*Part 1 judgement → Low risk (awareness present, but no deviations attributable to that awareness).*

**Part 2 – analysis**

| **Signalling question** | **Answer** | **Reasoning** |
| --- | --- | --- |
| **2.6 Was an appropriate analysis used?** | **Y** | All 20 participants per group are analysed at each time-point; no exclusions or per-protocol filtering are reported (tables give n = 20 throughout). |

*Part 2 judgement → Low risk.*

**Domain-level judgement: Low risk** *Open-label, but no evidence of differential deviations and a complete “as randomised” analysis.*

**Domain 3 – Bias due to missing outcome data**

| **Signalling question** | **Answer** | **Reasoning** |
| --- | --- | --- |
| **3.1 Were outcome data available for nearly all participants?** | **Y** | The study retained **all 60 randomised subjects**; results are reported for the full 20 + 20 + 20 sample at every time-point (Tables 1–2) . |

**Domain-level judgement: Low risk**

**Domain 4 – Bias in measurement of the outcome**

| **Signalling question** | **Answer** | **Reasoning** |
| --- | --- | --- |
| **4.1 Was the outcome measurement method inappropriate?** | **N** | *S. mutans* counts were determined with the commercially validated **CRT bacteria test** on selective agar plates, following manufacturer instructions . |
| **4.2 Could measurement differ between groups?** | **N** | The same saliva-collection protocol and CRT kit were applied to **all** groups at each visit. |
| **4.3 Were outcome assessors aware of intervention?** | **N** | “**All laboratory procedures were carried out without the personnel knowing the allocation of saliva samples to bracket groups**” . |

**Domain-level judgement: Low risk**

**Domain 5 – Bias in selection of the reported result**

| **Signalling question** | **Answer** | **Reasoning** |
| --- | --- | --- |
| **5.1 Was the analysis plan pre-specified?** | **NI** | No protocol or statistical analysis plan is referenced. |
| **5.2 Result selected from multiple outcome measurements?** | **PN** | The outcome was measured at T0, T1, T2 and **all three time-points are reported**; no unreported alternative scales are suggested. |
| **5.3 Result selected from multiple analyses?** | **PN** | Only straightforward group comparisons and regression analyses are presented; no evidence of selective reporting. |

**Domain-level judgement: Some concerns** *Absence of a publicly available, dated analysis plan prevents a low-risk rating.*

**Overall risk-of-bias judgement: Some concerns**

Although outcome measurement, missing data, and deviations from intervention are at low risk of bias, **uncertainty about allocation concealment and about pre-specification of the statistical analysis** means the trial cannot be judged at overall low risk.

## Study 38

**DOMAIN 1 – Bias arising from the randomization process**

| **Signalling question** | **Code** | **Quote-based reasoning** |
| --- | --- | --- |
| 1.1 Was the allocation sequence random? | **Y** | “*…using a* ***computerized random number generator with random block sizes***” |
| 1.2 Was the allocation sequence concealed until participants were enrolled and assigned? | **PY** | “*…each received the allocations* ***sealed in closed, sequentially numbered envelopes***” |
| 1.3 Did baseline differences suggest a problem with randomization? | **N** | “*There were* ***no relevant differences in most of the baseline characteristics*** *between the two arms*” (Table I) |

**Domain 1 Judgment:** **Low risk** *Allocation was generated randomly (1.1 = Y) and probably concealed (1.2 = PY); groups were balanced (1.3 = N) → Low risk.*

**DOMAIN 2 – Bias due to deviations from intended interventions (effect of assignment)**

| **Signalling question** | **Code** | **Quote-based reasoning** |
| --- | --- | --- |
| 2.1 Were participants aware of their assigned intervention? | **Y** | Distinct open procedures (multiple PEI needle injections vs one RFA probe) were described with **no mention of participant blinding** |
| 2.2 Were carers/personnel aware? | **Y** | Same rationale: operators performed visibly different procedures; blinding not reported |
| 2.3 Were there deviations from intended intervention due to the trial context? | **N** | “*Patients were evaluated … and,* ***if needed, were treated once again by the same technique***” – re-treatment pre-specified, not ad-hoc |
| 2.6 Was an appropriate analysis used to estimate the effect of assignment? | **Y** | “*An* ***intention-to-treat*** *analysis was carried out for the primary end-point*” |

*Part 1 (2.1–2.5) = Low risk (open-label but no protocol-deviating behaviour); Part 2 = Low risk (ITT).*

**Domain 2 Judgment:** **Low risk** *Open label yet no intervention-related deviations and ITT used.*

**DOMAIN 3 – Bias due to missing outcome data**

| **Signalling question** | **Code** | **Quote-based reasoning** |
| --- | --- | --- |
| 3.1 Were outcome data available for nearly all participants? | **N** | At 1 year, 62/70 (RFA) and 51/69 (PEI) were assessed; however the ITT analysis **included all 139 randomised patients** |
| 3.2 Evidence that results were not biased by missing data? | **Y** | Missing participants were counted as **non-CR in the ITT** primary analysis, a conservative approach |

**Domain 3 Judgment:** **Low risk** *Although some data were missing (3.1 = N), the conservative ITT analysis (3.2 = Y) makes bias unlikely.*

**DOMAIN 4 – Bias in measurement of the outcome**

| **Signalling question** | **Code** | **Quote-based reasoning** |
| --- | --- | --- |
| 4.1 Was the method of measuring the outcome inappropriate? | **N** | CR defined by *“computed tomography/magnetic resonance detection of a non-enhanced area of necrosis/scar”* |
| 4.2 Could measurement differ between groups? | **N** | Same imaging protocol described for both arms |
| 4.3 Were outcome assessors aware of intervention? | **Y** | “*Evaluations of CT by a* ***‘blinded’ observer were considered not feasible***” |
| 4.4 Could awareness influence assessment? | **PY** | Judging residual arterial enhancement is partially subjective; awareness might bias borderline cases. |
| 4.5 Was assessment likely influenced? | **PN** | Imaging criteria are largely objective; major misclassification unlikely. |

**Domain 4 Judgment:** **Some concerns** *Unblinded radiologists (4.3 = Y) with a semi-subjective outcome yield some, but not high, risk (4.5 = PN).*

**DOMAIN 5 – Bias in selection of the reported result**

| **Signalling question** | **Code** | **Quote-based reasoning** |
| --- | --- | --- |
| 5.2 Result selected from multiple outcome measurements? | **N** | Only one prespecified primary outcome (1-year CR) is reported. |
| 5.3 Result selected from multiple eligible analyses? | **PN** | Both ITT and per-protocol shown; ITT defined a priori; little scope for selective reporting. |
| 5.1 Analysis plan pre-specified? | **PY** | Trial was **registered (NCT00355212)** and approved in 2000, implying a prespecified plan |

**Domain 5 Judgment:** **Low risk** *No evidence of selective outcome or analysis reporting; registration supports prespecification.*

**Overall Risk-of-Bias Judgment: Some concerns**

*Domains 1–3 & 5 = Low risk; Domain 4 = Some concerns → Overall = Some concerns.*

## Study 39

**RoB 2.0 assessment for the primary outcome = *State anxiety (STAI-Y1)* Trial: “Effects of Mindfulness-Based Stress Reduction on Medical and Premedical Students”

**DOMAIN 1 – Risk of bias arising from the randomisation process**

| **Signalling question** | **Answer** | **Reasoning** |
| --- | --- | --- |
| 1.1 Was the allocation sequence random? | **NI** | The paper states participants were “randomly assigned to an intervention group or a wait-list control group” but gives no information on the method used to generate the sequence . |
| 1.2 Was the allocation sequence concealed until participants were enrolled? | **NI** | No description of any concealment mechanism (central randomisation, opaque envelopes, etc.) is provided in the report. |
| 1.3 Did baseline differences suggest a problem with randomisation? | **N** | The authors report χ² tests showing no significant differences in gender, ethnicity or medical-school status between groups . |

**Domain 1 judgement: Some concerns** *Allocation concealment is not reported (1.2 = NI). With no baseline imbalance (1.3 = N) the algorithm yields “Some concerns”.*

**DOMAIN 2 – Risk of bias due to deviations from intended interventions (effect of assignment)**

| **Signalling question** | **Answer** | **Reasoning** |
| --- | --- | --- |
| 2.1 Were participants aware of their assignment? | **Y** | An 8-week elective mindfulness course versus wait-list is inherently open-label; no blinding is claimed. |
| 2.2 Were carers/personnel aware? | **Y** | Class facilitators obviously knew they were delivering the intervention; no blinding described. |
| 2.3 Were there deviations from intended intervention due to the trial context? | **N** | The report does not mention cross-overs or compensatory behaviours; each arm followed its scheduled activities. |
| 2.4 Were these deviations likely to affect outcome? | – | Not applicable (2.3 = N). |
| 2.5 Were deviations balanced between groups? | – | Not applicable. |
| 2.6 Was an appropriate analysis used (ITT/mITT)? | **PN** | Five of 78 randomised participants (6 %) provided no post-test data and were excluded from analysis; ITT is not mentioned . |
| 2.7 If not, was the analysis likely to substantially bias results? | **PN** | Attrition is modest and similar between arms (1 in intervention, 4 in control); bias is possible but probably small. |

*Part 1 (2.1–2.5) = Low risk (open-label but no important deviations).* *Part 2 (2.6–2.7) = Some concerns.*

**Domain 2 judgement: Some concerns** *Lack of clear ITT analysis introduces some analytical concern.*

**DOMAIN 3 – Risk of bias due to missing outcome data**

| **Signalling question** | **Answer** | **Reasoning** |
| --- | --- | --- |
| 3.1 Were outcome data available for nearly all participants? | **PN** | 73/78 (94 %) provided STAI post-test scores (≥95 % threshold not quite met) . |
| 3.2 Evidence that results not biased by missing data? | **N** | No sensitivity analysis or justification regarding the impact of the five missing cases is offered. |
| 3.3 Could missingness depend on true outcome value? | **NI** | Reasons for four control-group non-responders are not reported. |
| 3.4 Is it likely that missingness depended on the outcome? | **NI** | Insufficient information. |

**Domain 3 judgement: High risk** *Because <95 % data were available and there is no information to dismiss outcome-dependent missingness, algorithm leads to High risk.*

**DOMAIN 4 – Risk of bias in measurement of the outcome**

| **Signalling question** | **Answer** | **Reasoning** |
| --- | --- | --- |
| 4.1 Was the outcome measurement method inappropriate? | **N** | State anxiety was measured with the validated STAI-Y1 questionnaire. |
| 4.2 Could measurement differ between groups? | **N** | Both groups completed identical self-report forms at identical time-points. |
| 4.3 Were outcome assessors aware of intervention? | **Y** | For self-report outcomes the participant is the assessor, and participants were unblinded. |
| 4.4 Could awareness have influenced outcome measurement? | **Y** | State-anxiety ratings are subjective and easily influenced by expectations. |
| 4.5 Is it likely that assessment was influenced? | **PY** | Expectation and social-desirability effects are plausible, though not demonstrated. |

**Domain 4 judgement: High risk** *Self-reported, subjective outcome with unblinded assessors gives high risk.*

**DOMAIN 5 – Risk of bias in selection of the reported result**

| **Signalling question** | **Answer** | **Reasoning** |
| --- | --- | --- |
| 5.1 Was the analysis pre-specified? | **NI** | No protocol or dated SAP is cited. |
| 5.2 Result selected from multiple outcome measurements? | **PN** | STAI-Y1 is a standard single measure; no alternative scales/time-points for state anxiety were mentioned. |
| 5.3 Result selected from multiple eligible analyses? | **PN** | Analysis appears straightforward (MANOVA then univariate tests) with no evidence of selective model choice. |

**Domain 5 judgement: Some concerns** *Absence of a pre-registered analysis plan leaves some uncertainty.*

**Overall risk-of-bias judgement: High risk**

*The study shows high risk in Domains 3 (missing data) and 4 (measurement of outcome), and some concerns elsewhere; therefore the overall risk of bias for the state-anxiety outcome is High.*

## Study 40

**PRIMARY OUTCOME ASSESSED: Short Falls Efficacy Scale-International (Short FES-I)**

**DOMAIN 1 – Bias arising from the randomisation process**

| **Signalling question** | **Code** | **Reasoning** |
| --- | --- | --- |
| 1.1 Was the allocation sequence random? | **Y** | “An online **computer-generated random allocation** was done by an independent randomisation centre with stratification for sex.” |
| 1.2 Was the allocation sequence concealed until participants were enrolled and assigned? | **PY** | Allocation was performed by an **independent randomisation centre**, suggesting central concealment, but the mechanism (e.g. web portal/telephone) is not explicitly described. |
| 1.3 Did baseline differences suggest a problem with randomisation? | **N** | “**Other baseline characteristics did not differ significantly** … between groups at T0.” |

**Domain 1 Judgement:** **Low risk** *1.2 = PY and 1.1 = Y → proceed to 1.3; 1.3 = N → Low risk.*

**DOMAIN 2 – Bias due to deviations from intended interventions (effect of assignment)**

| **Signalling question** | **Code** | **Reasoning** |
| --- | --- | --- |
| 2.1 Were participants aware of their assignment? | **Y** | “…the **patients themselves were aware of the intervention arm** they were allocated to.” |
| 2.2 Were carers/personnel aware of assignments? | **PY** | Therapists delivered eight individual sessions; no blinding is reported, so awareness is very likely. |
| 2.3 Were there deviations from intended intervention due to the trial context? | **N** | The report describes high adherence and no crossover or protocol deviations linked to awareness. |
| 2.4 If Y/PY to 2.3: could these affect outcome? | — | (Not triggered) |
| 2.5 If Y/PY to 2.3: were deviations balanced? | — | (Not triggered) |
| 2.6 Was an appropriate analysis used (ITT)? | **Y** | Results were based on **intention-to-treat analyses** with multiple imputation. |
| 2.7 Potential for substantial bias from analysis? | — | (Not triggered) |

**Domain 2 Judgement:** **Low risk** *Part 1: Although participants/personnel were unblinded (2.1/2.2 = Y/PY), no treatment-deviations were reported (2.3 = N) → Part 1 Low risk. Part 2: ITT used (2.6 = Y) → Low risk. Combined → Low risk.*

**DOMAIN 3 – Bias due to missing outcome data**

| **Signalling question** | **Code** | **Reasoning** |
| --- | --- | --- |
| 3.1 Were outcome data available for nearly all participants? | **N** | 115 randomised; **96 (83 %)** provided outcome data at T2. |
| 3.2 Evidence that results not biased by missing data? | **Y** | Authors performed **multiple imputation** for all outcomes and reported pooled ITT results. |
| 3.3 Could missingness depend on true outcome value? | — | (Not assessed because 3.2 = Y) |
| 3.4 Is it likely that it did? | — | (Not triggered) |

**Domain 3 Judgement:** **Low risk** *Although <95 % data (3.1 = N), robust multiple-imputation ITT analyses were applied (3.2 = Y) → Low risk.*

**DOMAIN 4 – Bias in measurement of the outcome**

| **Signalling question** | **Code** | **Reasoning** |
| --- | --- | --- |
| 4.1 Was the outcome measurement method inappropriate? | **N** | Short FES-I is a **validated scale** for fear of falling. |
| 4.2 Could measurement differ between groups? | **N** | Same instrument administered at identical time points for both groups; no alternative methods described. |
| 4.3 Were outcome assessors aware of assignment? | **N** | “The trained assessors … were **blind to patients’ allocation**.” |
| 4.4–4.5 | — | (Not triggered) |

**Domain 4 Judgement:** **Low risk** *Validated instrument, identical procedures, blinded assessors.*

**DOMAIN 5 – Bias in selection of the reported result**

| **Signalling question** | **Code** | **Reasoning** |
| --- | --- | --- |
| 5.2 Result selected from multiple measurements? | **PN** | Only the pre-specified Short FES-I (primary outcome) is reported; no alternative scales/time-points omitted. |
| 5.3 Result selected from multiple analyses? | **PN** | A single ANCOVA ITT model is described; no unexplained alternative analyses reported. |
| 5.1 Was the analysis pre-specified before unblinded data access? | **PN** | Trial was registered (ISRCTN79191813) , but no dated protocol/SAP is cited, so pre-specification cannot be verified. |

**Domain 5 Judgement:** **Some concerns** *5.2 and 5.3 = PN (no clear evidence of selective reporting) → evaluate 5.1; 5.1 = PN (uncertain pre-specification) → Some concerns.*

**OVERALL RISK-OF-BIAS JUDGEMENT: Some concerns**

*Domains 1-4 = Low risk; Domain 5 = Some concerns (unclear pre-specified analysis plan) → Overall risk of bias judged “Some concerns.”*

## Study 41

**Completed RoB 2.0 assessment for the Awakening and Breathing Controlled (ABC) trial
 (primary outcome = 28-day ventilator-free days)**

**DOMAIN 1 Risk of bias arising from the randomisation process**

| **Signalling question** | **Answer** | **Reasoning** |
| --- | --- | --- |
| **1.1 Was the allocation sequence random?** | **Y** | “A **computer-generated, permuted-block randomisation scheme** was stratified according to study centre” |
| **1.2 Was the allocation sequence concealed until participants were enrolled and assigned?** | **Y** | “Each assignment was designated on a tri-folded piece of paper enclosed in a **consecutively numbered, sealed, opaque envelope**” |
| **1.3 Did baseline differences suggest a problem with randomisation?** | **N** | “**The two groups were similar at baseline** (Table 1)” |

**Domain 1 judgement – Low risk** *1.2 = Y and 1.1 = Y ⇒ proceed to 1.3; 1.3 = N ⇒ Low risk.*

**DOMAIN 2 Risk of bias due to deviations from intended interventions (effect of assignment)**

| **Signalling question** | **Answer** | **Reasoning** |
| --- | --- | --- |
| **2.1 Were participants aware of their assigned intervention?** | **Y** | “**Research personnel and intensive-care staff were not blinded** to patient allocation because blinding is not possible in a study of this kind” |
| **2.2 Were carers/personnel aware of the intervention assignments?** | **Y** | Same quote as 2.1 |
| **2.3 Were there deviations from intended intervention due to the trial context?** | **PN** | No explicit deviations attributable to awareness are reported; sedation practice differences (e.g., sedatives held before SBT in 31 % of controls) were allowed by protocol and unlikely to reflect systematic non-adherence |
| **2.6 Was an appropriate analysis used to estimate the effect of assignment?** | **Y** | “**Data were analysed with an intention-to-treat approach**” |

Part 1 = Low risk (2.3 = PN); Part 2 = Low risk (2.6 = Y) → **Domain 2 judgement – Low risk**

**DOMAIN 3 Risk of bias due to missing outcome data**

| **Signalling question** | **Answer** | **Reasoning** |
| --- | --- | --- |
| **3.1 Were outcome data available for nearly all participants?** | **Y** | 336 randomised vs 335 analysed; only 3 (<1 %) lost to follow-up (Figure 2) |

**Domain 3 judgement – Low risk** *(>95 % data available; no further concerns).*

**DOMAIN 4 Risk of bias in measurement of the outcome**

| **Signalling question** | **Answer** | **Reasoning** |
| --- | --- | --- |
| **4.1 Was the method of measuring the outcome inappropriate?** | **N** | Ventilator-free days were calculated from objective ventilator logs; method clearly defined |
| **4.2 Could measurement differ between groups?** | **N** | Same protocol and ICU record systems used for both groups |
| **4.3 Were outcome assessors aware of intervention?** | **Y** | As per 2.1 (unblinded staff) |
| **4.4 Could awareness have influenced outcome assessment?** | **PN** | Outcome (days alive and off ventilator) is objective and extracted from routine records, so influence unlikely. |

Because 4.1 = N, 4.2 = N, 4.3 = Y but 4.4 = PN ⇒ **Domain 4 judgement – Low risk**

**DOMAIN 5 Risk of bias in selection of the reported result**

| **Signalling question** | **Answer** | **Reasoning** |
| --- | --- | --- |
| **5.2 Result selected from multiple outcome measurements?** | **N** | Only one definition of ventilator-free days (48 h unassisted, 28-day window) is described and reported |
| **5.3 Result selected from multiple eligible analyses?** | **N** | Primary analysis prespecified as ITT with non-parametric CI; no alternative analyses presented |
| **5.1 Was analysis plan prespecified?** | **PY** | Trial registered (NCT00097630) and primary endpoint stated a priori, but no dated SAP provided |

5.2 = N and 5.3 = N ⇒ evaluate 5.1; 5.1 = PY ⇒ **Domain 5 judgement – Low risk**

**Overall risk-of-bias judgement – Low risk**

All five domains are at low risk; no domain shows “some concerns” or “high risk”. Therefore the overall confidence in the effect estimate for ventilator-free days is **Low risk of bias**.

## Study 42

**RoB 2.0 assessment – Use of BioGlue Surgical Adhesive in Hypospadias Repair** *Primary outcome assessed: incidence of urethro-cutaneous fistula*

**DOMAIN 1 – Risk of bias arising from the randomization process**

| **Signalling question** | **Code** | **Reasoning** |
| --- | --- | --- |
| 1.1 Was the allocation sequence random? | **PN** | The paper states only that “**All patients were randomly divided into 2 equal groups**” with no description of how the sequence was generated . |
| 1.2 Was the allocation sequence concealed until participants were enrolled and assigned? | **PN** | The article provides **no information** on any concealment method (e.g., sealed envelopes, central randomization); none is mentioned in Methods. |
| 1.3 Did baseline differences suggest a problem with the randomization process? | **N** | Authors report that **“Demographic data and primary meatus location were similar in both groups (table 1)”** . |

**Domain 1 judgment: High risk** *Justification*: 1.2 = PN ⇒ per algorithm this alone gives **High risk**, irrespective of 1.1 or 1.3.

**DOMAIN 2 – Risk of bias due to deviations from intended interventions (effect of assignment)**

| **Signalling question** | **Code** | **Reasoning** |
| --- | --- | --- |
| 2.1 Were participants aware of their assigned intervention? | **PY** | No blinding is described; treatments differ surgically (“BioGlue … applied … Patients in Group II were operated on utilizing a routine technique”) . |
| 2.2 Were carers/personnel aware of the intervention? | **Y** | **“The operations were performed by a single surgeon (B.C.)”** – the operating surgeon necessarily knew group assignment . |
| 2.3 Were there deviations from intended intervention that arose because of the experimental context? | **PN** | No protocol deviations, cross-overs, or additional co-interventions are reported. |
| 2.4 If deviations occurred, were they likely to affect the outcome? | — | Not triggered (2.3 = PN). |
| 2.5 Were deviations balanced between groups? | — | Not triggered. |
| 2.6 Was an appropriate analysis used to estimate the effect of assignment? | **Y** | All 40 randomized patients were analysed with chi-square/Fisher tests; no exclusions reported (“GraphPad … chi square and Fisher test”) . |
| 2.7 Was there potential for substantial bias due to inappropriate analysis? | — | Not triggered (2.6 = Y). |

**Domain 2 judgment: Low risk** *Justification*: Part 1 Low risk (2.3 = PN) and Part 2 Low risk (2.6 = Y) ⇒ Domain 2 Low risk.

**DOMAIN 3 – Risk of bias due to missing outcome data**

| **Signalling question** | **Code** | **Reasoning** |
| --- | --- | --- |
| 3.1 Were outcome data available for nearly all participants? | **Y** | Results reported for all 40 patients; fistula counts (4 vs 3) sum to the full sample (20 + 20) . |
| 3.2–3.4 | — | Not triggered (3.1 = Y). |

**Domain 3 judgment: Low risk** *Justification*: Complete follow-up (>95 %) for the primary outcome.

**DOMAIN 4 – Risk of bias in measurement of the outcome**

| **Signalling question** | **Code** | **Reasoning** |
| --- | --- | --- |
| 4.1 Was the method of measuring the outcome inappropriate? | **N** | Fistula presence determined by postoperative clinical examination – a standard, objective method (“… urethro-cutaneous fistula, suture line breakdown and meatal stenosis were recorded”) . |
| 4.2 Could measurement differ between groups? | **N** | Same follow-up schedule applied to all patients (“patients … come back after 3 months, after 1 year…”) . |
| 4.3 Were outcome assessors aware of intervention? | **Y** | Follow-up likely performed by the operating surgeon (single-surgeon study) . |
| 4.4 Could assessor awareness influence outcome assessment? | **PN** | Fistula detection is mainly objective (visible leak), so influence is unlikely though possible. |
| 4.5 Is it likely assessment was influenced? | **PN** | As above, any bias is improbable to meaningfully change detection of a physical fistula. |

**Domain 4 judgment: Low risk** *Justification*: Although assessors were unblinded (4.3 = Y), the outcome is objective and influence unlikely (4.4 & 4.5 = PN) ⇒ Low risk.

**DOMAIN 5 – Risk of bias in selection of the reported result**

| **Signalling question** | **Code** | **Reasoning** |
| --- | --- | --- |
| 5.1 Was the analysis pre-specified? | **NI** | No protocol or SAP is referenced; timing not stated. |
| 5.2 Result selected from multiple outcome measurements? | **PN** | Fistula was measured at a single postoperative time frame; no alternate scales reported. |
| 5.3 Result selected from multiple eligible analyses? | **PN** | Only basic between-group comparison is presented; no indication of multiple models. |

**Domain 5 judgment: Some concerns** *Justification*: 5.2 & 5.3 = PN → evaluate 5.1; with 5.1 = NI ⇒ **Some concerns**.

**Overall risk-of-bias judgment: High risk**

**Rationale**: Domain 1 is **High risk** (unclear allocation concealment). Despite other domains being Low or Some concerns, a single High-risk domain yields an Overall judgment of **High risk** for bias in the estimated effect of BioGlue on urethro-cutaneous fistula incidence.

## Study 43

**PRIMARY OUTCOME ASSESSED** Anti-S IgG Immune-Status Ratio (ISR) 4 weeks ± 7 days after booster dose

**DOMAIN 1 – Bias arising from the randomization process**

| **Signalling question** | **Code** | **Reasoning** |
| --- | --- | --- |
| 1.1 Was the allocation sequence random? | **Y** | “The balanced block randomization list was generated using the block size of 4 and sample size through the research institute’s web-based software.” |
| 1.2 Was the allocation sequence concealed until participants were enrolled and assigned? | **PY** | Vaccine administrators used the list but “all other team members were blinded to the randomization list.” Exact concealment method (e.g., central system) not described. |
| 1.3 Did baseline differences suggest a problem? | **N** | The authors report “no statistically significant differences between the two randomized groups’ baseline characteristics.” (Table 1) |

**Domain 1 Judgment: Low risk** *1.2=PY; 1.1=Y → proceed to 1.3; 1.3=N → Low risk.*

**DOMAIN 2 – Bias due to deviations from intended interventions (effect of assignment)**

| **Signalling question** | **Code** | **Reasoning** |
| --- | --- | --- |
| 2.1 Were participants aware of their assignment? | **N** | Trial described as “participant and observer-blinded.” |
| 2.2 Were carers or trial personnel aware? | **PY** | Vaccine administrators enrolled and dosed participants using the list; other staff were blinded. |
| 2.3 Were there deviations from intended intervention due to the trial context? | **N** | All randomized patients received the allocated booster; no cross-overs reported. |
| 2.6 Was an appropriate analysis used (effect of assignment)? | **Y** | “In a randomized clinical trial, the intention-to-treat (ITT) is the primary analysis… ITT and per-protocol analyses will invariably produce identical results.” |

*Part 1 (2.1-2.5) = Low risk (2.3=N). Part 2 (2.6-2.7) = Low risk (2.6=Y).*

**Domain 2 Judgment: Low risk**

**DOMAIN 3 – Bias due to missing outcome data**

| **Signalling question** | **Code** | **Reasoning** |
| --- | --- | --- |
| 3.1 Were outcome data available for nearly all participants? | **Y** | Only “Two of the thirty-one blood samples collected in the heterologous arm were lost”; 59/61 (96.7 %) had data. |

**Domain 3 Judgment: Low risk** *(>95 % outcome availability).*

**DOMAIN 4 – Bias in measurement of the outcome**

| **Signalling question** | **Code** | **Reasoning** |
| --- | --- | --- |
| 4.1 Was the method of measuring the outcome inappropriate? | **N** | ISR measured with a validated semi-quantitative immunoassay (ChemoBind SARS-CoV-2 Neutralizing Antibody Test Kit). |
| 4.2 Could measurement differ between groups? | **N** | Same assay and schedule applied to both groups. |
| 4.3 Were outcome assessors aware of the intervention? | **N** | “Laboratory assessors [were] blinded to the randomization list.” |

**Domain 4 Judgment: Low risk**

**DOMAIN 5 – Bias in selection of the reported result**

| **Signalling question** | **Code** | **Reasoning** |
| --- | --- | --- |
| 5.2 Result selected from multiple outcome measurements? | **PN** | Primary outcome, assay, and single time-point were pre-specified; no alternative measurements presented. |
| 5.3 Result selected from multiple eligible analyses? | **PN** | Only ITT (and identical PP) analyses reported; no unexplained subgroup or model selection. |
| 5.1 Was the analysis plan pre-specified? | **PY** | Trial was registered (IRCT20140818018842N24), but protocol/SAP not provided; analyses align with stated objectives. |

*5.2 and 5.3 = PN → consider 5.1. With 5.1=PY → Low risk.*

**Domain 5 Judgment: Low risk**

**OVERALL RISK-OF-BIAS JUDGMENT: Low risk**

*All five domains were judged Low risk, giving high confidence that the estimated effect on anti-S IgG ISR is not meaningfully biased.*

## Study 44

**Risk-of-Bias (RoB 2, 22 Aug 2019) assessment for the primary outcome
 “Radiological reflux score at Day 2”**

**DOMAIN 1 – Bias arising from the randomization process**

| **Signalling question** | **Answer** | **Reasoning** |
| --- | --- | --- |
| 1.1 Was the allocation sequence random? | **PN** | The study says participants “were **randomly allocated** to 2 different arms” but gives **no description of the sequence generation method** . |
| 1.2 Was the allocation sequence concealed until participants were enrolled and assigned? | **PN** | Allocation used “**sealed envelopes** containing information about the type of stent to be used,” with no details on opacity, numbering or tamper-proofing . |
| 1.3 Did baseline differences suggest a problem? | **N** | Baseline Table 2 shows age, sex, tumour size and histology are similar, and authors state **“No statistical differences were noted between the groups”** . |

**Domain 1 judgment: High risk** *Justification*: Allocation concealment is probably inadequate (1.2 = PN); per RoB 2 algorithm this alone makes Domain 1 high risk.

**DOMAIN 2 – Bias due to deviations from intended interventions (effect of assignment)**

| **Signalling question** | **Answer** | **Reasoning** |
| --- | --- | --- |
| 2.1 Were participants aware of their assignment? | **N** | “**Patients were blinded to the type of stent received**” . |
| 2.2 Were carers/personnel aware? | **PY** | Endoscopists inserted visibly different stents; blinding is not mentioned: “All procedures were performed … by experienced endoscopists” . |
| 2.3 Were there deviations due to the trial context? | **PN** | No departures from the single-procedure intervention are reported. |
| 2.6 Was an appropriate analysis used (ITT)? | **PN** | Two participants lost before outcome assessment “were **excluded from the study**” ; no intention-to-treat analysis is reported. |
| 2.7 Substantial bias from inappropriate analysis? | **PY** | Excluding 5 % of randomised participants (all from one arm) could influence the Day-2 outcome. |

**Domain 2 judgment: High risk** *Justification*: Part 1 Low risk (2.3 = PN), but Part 2 High risk (2.6 = PN & 2.7 = PY) ⇒ Domain 2 = High risk.

**DOMAIN 3 – Bias due to missing outcome data**

| **Signalling question** | **Answer** | **Reasoning** |
| --- | --- | --- |
| 3.1 Were outcome data available for nearly all? | **Y** | 38 / 40 (95 %) had Day-2 radiology; only two early losses . |

**Domain 3 judgment: Low risk** *Justification*: ≥95 % of randomised participants contributed data; missingness is minimal.

**DOMAIN 4 – Bias in measurement of the outcome**

| **Signalling question** | **Answer** | **Reasoning** |
| --- | --- | --- |
| 4.1 Was outcome measurement inappropriate? | **N** | A predefined **quantitative radiological score (0–9)** was used . |
| 4.2 Could measurement differ between groups? | **N** | Same radiological procedure for all. |
| 4.3 Were outcome assessors aware? | **N** | “**The radiologist interpreting the images was blinded to the type of stent received**” . |

**Domain 4 judgment: Low risk** *Justification*: Valid, uniform, blinded assessment.

**DOMAIN 5 – Bias in selection of the reported result**

| **Signalling question** | **Answer** | **Reasoning** |
| --- | --- | --- |
| 5.2 Result selected from multiple measurements? | **PN** | Radiological reflux was measured only once (Day 2); no other scales/time-points described. |
| 5.3 Result selected from multiple analyses? | **PN** | Only the Wilcoxon comparison specified for this outcome; no alternative models presented. |
| 5.1 Analysis pre-specified? | **NI** | No protocol or SAP referenced; timing of analysis plan unclear. |

**Domain 5 judgment: Some concerns** *Justification*: No evidence of selective measurement/analysis, but absence of a publicly available pre-specification yields some concerns.

**Overall risk-of-bias judgment: High risk**

*Domains 1 and 2 are each at High risk; therefore the overall risk of bias for the primary outcome is High.*

## Study 45

**Revised Cochrane RoB 2.0 evaluation – primary outcome: “time from induction to vaginal delivery”**

**DOMAIN 1 – Bias arising from the randomisation process**

| **Signalling question** | **Answer** | **Reasoning** |
| --- | --- | --- |
| 1.1 Was the allocation sequence random? | **Y** | “The allocation of treatment was generated using **a computer random schedule with a block size of six** at the start of the study” |
| 1.2 Was the allocation sequence concealed until participants were enrolled and assigned? | **Y** | “Assignment was concealed by placement in **consecutively numbered, opaque, sealed envelopes** drawn in consecutive order by an administrator, who was unaware of which agent was allocated until the envelope was opened” |
| 1.3 Did baseline differences suggest a problem with randomisation? | **N** | “**The two groups were similar regarding baseline characteristics**… age, body mass index, gestational age, smoking, indication for IOL, and Bishop score” |

**Domain 1 judgement: Low risk** *Sequence was random (1.1 = Y), concealed (1.2 = Y) and baseline covariates balanced (1.3 = N).*

**DOMAIN 2 – Bias due to deviations from intended interventions (effect of assignment)**

| **Signalling question** | **Answer** | **Reasoning** |
| --- | --- | --- |
| 2.1 Were participants aware of their assignment? | **Y** | “Blinding of the participant… was not possible” |
| 2.2 Were carers/personnel aware? | **Y** | Same sentence as above |
| 2.3 Were there deviations from the intended intervention because of the experimental context? | **PY** | 4/100 women randomised to MVI **received oral misoprostol instead** |
| 2.4 Were these deviations likely to affect the outcome? | **PN** | Crossover involved **4 %** of the MVI arm; effect on median time to delivery is probably minimal. |
| 2.5 Were deviations balanced between groups? | **N** | The crossover occurred **only in the MVI arm**; none reported in the oral arm |
| — **Part-1 judgement** | **Some concerns** | Open-label trial with a small, unbalanced crossover, but impact probably minor. |
| 2.6 Was an appropriate analysis used (e.g. ITT)? | **NI** | Authors state “196 women left for the analysis” (98 %) but **do not specify ITT/per-protocol** |
| 2.7 Risk of substantial bias from inappropriate analysis? | **PN** | No evidence of per-protocol‐only analysis; results reported for almost all randomised women. |

**Domain 2 judgement: Some concerns** *Open-label design and a small, one-sided crossover create some uncertainty; analysis approach not explicitly described as ITT.*

**DOMAIN 3 – Bias due to missing outcome data**

| **Signalling question** | **Answer** | **Reasoning** |
| --- | --- | --- |
| 3.1 Were outcome data available for nearly all participants? | **Y** | 199 randomised → **196 analysed (98 %)** |

**Domain 3 judgement: Low risk** *Outcome data available for > 95 % of participants.*

**DOMAIN 4 – Bias in measurement of the outcome**

| **Signalling question** | **Answer** | **Reasoning** |
| --- | --- | --- |
| 4.1 Was the method of measuring the outcome inappropriate? | **N** | Outcome is an **objective time interval** recorded in routine obstetric charts |
| 4.2 Could measurement differ between groups? | **N** | Same hospital records and timing rules used for both groups. |
| 4.3 Were outcome assessors aware of the intervention? | **Y** | Open-label trial; delivery ward staff knew assignments |
| 4.4 Could awareness have influenced outcome assessment? | **PN** | Time to delivery is automatically clocked by documented start/end times, leaving little room for assessor discretion. |

**Domain 4 judgement: Low risk** *Objective, consistently recorded outcome unlikely to be biased by lack of blinding.*

**DOMAIN 5 – Bias in selection of the reported result**

| **Signalling question** | **Answer** | **Reasoning** |
| --- | --- | --- |
| 5.2 Result selected from multiple outcome measurements? | **PN** | Only one plausible way to measure “time to vaginal delivery” is reported. |
| 5.3 Result selected from multiple analyses? | **PN** | Primary analysis clearly described; no alternative models presented. |
| 5.1 Was the analysis pre-specified? | **PY** | Trial **registered (NCT02918110) and protocol developed following CONSORT** – but SAP not shown. |

**Domain 5 judgement: Low risk** *No evidence of selective measurement or analysis; registration suggests plans were made a-priori.*

**Overall risk-of-bias judgement: Some concerns**

*Only Domain 2 shows “Some concerns”; all other domains are Low risk. The overall confidence in the primary outcome estimate is therefore rated as* ***Some concerns*** *rather than high risk.*

## Study 46

**Primary outcome assessed:** change in serum cystatin C after 12 weeks (stated as the study’s primary endpoint)

**DOMAIN 1 – Bias arising from the randomization process**

| **Signalling question** | **Answer** | **Reasoning** |
| --- | --- | --- |
| 1.1 Was the allocation sequence random? | **Y** | “Generated from random numbers in a 1:1 manner based on a *computer-generated randomization sequence*” |
| 1.2 Was the allocation sequence concealed until participants were enrolled and assigned? | **Y** | “Allocation concealment by *opaque sequentially numbered sealed envelope*” |
| 1.3 Did baseline differences suggest a problem with randomization? | **N** | “*Both groups were well-matched in baseline clinical and laboratory parameters*” |

**Domain 1 judgment: Low risk** *Allocation was random (1.1 = Y) and concealed (1.2 = Y); baseline covariates balanced (1.3 = N) → Low risk.*

**DOMAIN 2 – Bias due to deviations from intended interventions (effect of assignment)**

| **Signalling question** | **Answer** | **Reasoning** |
| --- | --- | --- |
| 2.1 Were participants aware of their assignment? | **N** | The trial is described as “*double-blinded*” with a matching placebo |
| 2.2 Were carers/trial personnel aware? | **N** | Same “double-blinded… placebo-controlled” description applies |
| 2.3–2.5 Any deviations due to the trial context? | **N** | No cross-overs or protocol deviations reported; compliance checked by capsule count and no adverse reactions noted |
| 2.6 Appropriate analysis for effect of assignment? | **Y** | “Analyses were conducted based on *intention-to-treat (ITT) principle*” |

**Domain 2 judgment: Low risk** *Blinding adequate (2.1/2.2 = N) and no deviations (2.3 = N). ITT analysis used (2.6 = Y). Both Parts 1 and 2 = Low risk.*

**DOMAIN 3 – Bias due to missing outcome data**

| **Signalling question** | **Answer** | **Reasoning** |
| --- | --- | --- |
| 3.1 Were outcome data available for nearly all? | **N** | 75/80 participants (93.8 %) provided endpoint data; 5 dropped out |
| 3.2 Evidence results not biased by missing data? | **N** | No sensitivity analysis or imputation described. |
| 3.3 Could missingness depend on true outcome? | **PN** | Drop-outs were simply “*did not attend follow-up measurements*” with no indication of outcome-related reasons |

**Domain 3 judgment: Low risk** *Although <95 % follow-up (3.1 = N), missingness is unlikely related to cystatin C outcome (3.3 = PN) and no other concerns → Low risk per algorithm.*

**DOMAIN 4 – Bias in measurement of the outcome**

| **Signalling question** | **Answer** | **Reasoning** |
| --- | --- | --- |
| 4.1 Was outcome measurement inappropriate? | **N** | Cystatin C measured with a commercial ELISA kit following standard protocol |
| 4.2 Could measurement differ between groups? | **N** | Same central laboratory procedures applied to both arms (methods section) |
| 4.3 Were outcome assessors aware of intervention? | **NI** | Assessor blinding not reported. |
| 4.4 Could awareness influence assessment? | **PN** | Laboratory assay is objective and automated; assessor knowledge unlikely to affect read-outs. |

**Domain 4 judgment: Some concerns** *Objective assay lowers risk, but absence of explicit assessor blinding (4.3 = NI) yields “Some concerns.”*

**DOMAIN 5 – Bias in selection of the reported result**

| **Signalling question** | **Answer** | **Reasoning** |
| --- | --- | --- |
| 5.2 Result selected from multiple measurements? | **PN** | Only one clearly defined time-point (12 weeks) for cystatin C, matching stated endpoint |
| 5.3 Result selected from multiple analyses? | **PN** | No alternative analyses presented; standard ANCOVA/ITT described |
| 5.1 Was analysis pre-specified? | **NI** | No protocol or dated SAP cited. |

**Domain 5 judgment: Some concerns** *No evidence of selective reporting, but lack of a pre-registered analysis plan (5.1 = NI).*

**Overall risk-of-bias judgment: Some concerns**

*Domains 1–3 = Low risk; Domains 4 & 5 = Some concerns (no High-risk domains) → Overall = Some concerns.*

## Study 47

**Risk-of-Bias (RoB 2.0) assessment for the primary outcome = *maximal oxygen uptake (VO₂max)* in
 “Low-intensity rowing with blood-flow restriction over 5 weeks increases VO₂max in elite rowers: a randomized controlled trial”.

**DOMAIN 1 – Bias arising from the randomization process**

| **Signalling question** | **Answer** | **Reasoning** |
| --- | --- | --- |
| 1.1 Was the allocation sequence random? | **Y** | “Thirty-one elite rowers were … assigned to the intervention … or control group, **using the minimization method (Strata: Gender, Age, Height, VO₂max)**.” Minimization is an accepted random-sequence method in RoB 2. |
| 1.2 Was the allocation sequence concealed until enrolment? | **NI** | The Methods section describes the sequence method but gives **no information** on concealment procedures (central service, opaque envelopes, etc.). |
| 1.3 Did baseline differences suggest a problem with randomization? | **N** | Baseline Table 1 shows no significant differences between groups (all p ≥ 0.80). |

**Domain 1 judgement = Some concerns** *Allocation was clearly random (1.1 = Y) and groups were balanced (1.3 = N), but concealment was not reported (1.2 = NI). Under RoB 2 logic NI in 1.2 → Some concerns.*

**DOMAIN 2 – Bias due to deviations from intended interventions (effect of assignment)**

**Part 1 – deviations**

| **Q** | **A** | **Reasoning** |
| --- | --- | --- |
| 2.1 Participants aware? | **Y** | This was an exercise-training study; nothing indicates participant blinding and the intervention (elastic wraps during rowing) is obvious. |
| 2.2 Carers/personnel aware? | **Y** | Coaches applying/monitoring pBFR necessarily knew the assignment. |
| 2.3 Deviations due to that awareness? | **N** | Training logs show similar weekly volume, sessions, and intensity distribution between groups (all p > 0.56). |
| *Part 1 result* | Low risk | Awareness existed, but no deviations attributable to it (2.3 = N). |

**Part 2 – analysis**

| **Q** | **A** | **Reasoning** |
| --- | --- | --- |
| 2.6 Appropriate effect-of-assignment analysis? | **Y** | All 31 randomized athletes were analysed (n = 16 INT, 15 CON) in a 2 × 2 mixed ANOVA; no exclusions reported. |
| *Part 2 result* | Low risk |  |

**Domain 2 judgement = Low risk** *Both Part 1 and Part 2 are low risk.*

**DOMAIN 3 – Bias due to missing outcome data**

| **Q** | **A** | **Reasoning** |
| --- | --- | --- |
| 3.1 Nearly all data available? | **Y** | No drop-outs reported; sample sizes identical pre- and post-intervention (16 + 15 = 31). |
| *Subsequent questions not triggered* |  |  |

**Domain 3 judgement = Low risk** *(> 95 % outcome data; no indication of bias from missing data).*

**DOMAIN 4 – Bias in measurement of the outcome**

| **Q** | **A** | **Reasoning** |
| --- | --- | --- |
| 4.1 Method inappropriate? | **N** | VO₂max measured with breath-by-breath Metamax 3B system using a standard ramp protocol on Concept2 ergometer – a validated, objective method. |
| 4.2 Could measurement differ between groups? | **N** | Same ergometer, protocol, and equipment for both groups. |
| 4.3 Were assessors aware? | **Y** | Testing staff likely knew assignments; blinding not mentioned. |
| 4.4 Could awareness influence outcome? | **PN** | VO₂max is automatically recorded; assessor discretion minimal. |
| 4.5 Likely that assessment was influenced? | **PN** | Objective physiological read-out makes meaningful bias unlikely. |

**Domain 4 judgement = Low risk** *Measurement objective; even if assessors were aware, influence on VO₂max values is improbable.*

**DOMAIN 5 – Bias in selection of the reported result**

| **Q** | **A** | **Reasoning** |
| --- | --- | --- |
| 5.2 Result selected from multiple outcome measurements? | **PN** | Only one VO₂max time-point (post-intervention) is described; no alternative scales/time-points reported. |
| 5.3 Result selected from multiple analyses? | **PN** | A single mixed-model ANOVA is presented; no evidence of unreported alternative analyses. |
| 5.1 Was analysis pre-specified? | **NI** | No protocol or dated SAP referenced. |

**Domain 5 judgement = Some concerns** *Absence of a pre-registered analysis plan gives some concern despite lack of overt selective reporting.*

**OVERALL RISK-OF-BIAS JUDGEMENT = Some concerns**

*Domains 2–4 were low risk, but Domain 1 (randomization concealment) and Domain 5 (analysis pre-specification) each carry “Some concerns”; no domain was high risk.*

## Study 48

**Risk of Bias 2.0 assessment (primary outcome = *Time to recovery from diarrhoea*)**

**DOMAIN 1 – Bias arising from the randomisation process**

| **Signalling question** | **Answer** | **Reasoning** |
| --- | --- | --- |
| **1.1 Was the allocation sequence random?** | **Y** | “All randomisation procedures were performed using a **computer programme (GraphPad QuickCalcs)**” |
| **1.2 Was the allocation sequence concealed until participants were enrolled and assigned?** | **PY** | Sequence codes were “**entirely encrypted … Patients’ families, research investigators, the statistician and the attending healthcare workers were blinded to these codes**,” but the paper does not describe a central or envelope system. |
| **1.3 Did baseline differences suggest a problem with randomisation?** | **N** | Authors state “**There were no significant differences in age or gender distribution between the two groups**,” and Table 1 shows balanced characteristics. |

**Domain-level judgment:** **Low risk** **Justification:** Concealment probably adequate (1.2 = PY); random sequence clearly described (1.1 = Y); no baseline imbalance (1.3 = N) → Low risk.

**DOMAIN 2 – Bias due to deviations from intended interventions (effect of assignment)**

| **Signalling question** | **Answer** | **Reasoning** |
| --- | --- | --- |
| **2.1 Were participants aware of their assigned intervention?** | **N** | Trial is explicitly **double-blind**; participants were blinded. |
| **2.2 Were carers/personnel aware of the intervention?** | **N** | Same double-blind statement covers carers and investigators. |
| **2.3–2.5 Deviations related to awareness** | — | Skipped (2.1 & 2.2 = N ⇒ no risk pathway) |
| **2.6 Was an appropriate analysis used (ITT or similar)?** | **Y** | “**None of the participants discontinued the treatment**,” so all randomised children were analysed, consistent with an ITT approach. |

**Domain-level judgment:** **Low risk** **Justification:** Blinding maintained (Part 1 = Low risk) and all randomised participants analysed (Part 2 = Low risk).

**DOMAIN 3 – Bias due to missing outcome data**

| **Signalling question** | **Answer** | **Reasoning** |
| --- | --- | --- |
| **3.1 Were data available for nearly all participants?** | **Y** | 0/86 participants lost: “**None of the participants discontinued…**” |

**Domain-level judgment:** **Low risk** **Justification:** Complete outcome data for all randomised participants.

**DOMAIN 4 – Bias in measurement of the outcome**

| **Signalling question** | **Answer** | **Reasoning** |
| --- | --- | --- |
| **4.1 Was the outcome measurement method inappropriate?** | **N** | Primary outcome defined a-priori as “**period … until the passage of two consecutive semi-formed stools or no stool for 12 h**,” a standard, objective clinical criterion. |
| **4.2 Could measurement differ between groups?** | **N** | Same criteria and hospital staff for both groups. |
| **4.3 Were outcome assessors aware of intervention?** | **N** | Assessors were among the blinded healthcare workers. |

**Domain-level judgment:** **Low risk** **Justification:** Appropriate, identical measurement; assessors blinded.

**DOMAIN 5 – Bias in selection of the reported result**

| **Signalling question** | **Answer** | **Reasoning** |
| --- | --- | --- |
| **5.2 Result selected from multiple outcome measurements?** | **PN** | Only one logical way to measure “time to recovery”; paper reports that single metric. |
| **5.3 Result selected from multiple eligible analyses?** | **PN** | No evidence of multiple statistical models; main analysis reported. |
| **5.1 Was the analysis plan pre-specified?** | **PY** | Trial **registered (TCTR20190423004)** before enrolment; protocol not supplied but registration suggests pre-specification. |

**Domain-level judgment:** **Low risk** **Justification:** No signs of selective measurement/analysis; registration indicates prior planning.

**OVERALL RISK-OF-BIAS JUDGMENT: Low risk**

All five domains are at low risk; therefore the overall risk of bias for the primary outcome is Low.

## Study 49

**Risk-of-Bias (RoB 2.0) assessment for the primary outcome
 “Sustained return of spontaneous circulation (ROSC ≥ 20 min)”**

**Domain 1 – Bias arising from the randomization process**

| **Signalling question** | **Answer** | **Key evidence** |
| --- | --- | --- |
| 1.1 Allocation sequence random? | **PY** | Article states patients were “randomly assigned in a 1:1 ratio” but gives no details of sequence generation. |
| 1.2 Allocation sequence concealed? | **PY** | Randomization used “opaque, sealed envelope” packs; sequential numbering not described. |
| 1.3 Baseline imbalance? | **N** | Table 1 shows groups closely balanced for all reported characteristics. |

**Domain 1 judgment: Low risk** *Allocation was probably concealed (1.2 = PY) and probably random (1.1 = PY); baseline covariates balanced (1.3 = N).*

**Domain 2 – Bias due to deviations from intended interventions (effect of assignment)**

| **Signalling question** | **Answer** | **Key evidence** |
| --- | --- | --- |
| 2.1 Participants aware? | **N** | “Patients and surrogates were not informed of the assignments.” |
| 2.2 Carers/personnel aware? | **Y** | “On-site clinicians and in-hospital clinicians were aware of the trial-group assignments after randomization.” |
| 2.3 Protocol-non-adherent deviations due to awareness? | **PN** | Cross-over only after two failed attempts, as per protocol; no evidence of preferential deviations. |
| 2.6 Appropriate analysis (ITT)? | **Y** | Outcomes analysed in a “modified intention-to-treat population.” |

**Domain 2 judgment: Low risk** *Clinical staff were unblinded, but deviations were minor, balanced and handled with ITT analysis.*

**Domain 3 – Bias due to missing outcome data**

| **Signalling question** | **Answer** | **Key evidence** |
| --- | --- | --- |
| 3.1 Outcome data available for nearly all? | **Y** | Primary outcome available for 100 % of 1 479 analysed patients; ≤ 0.3 % missing for 30-day secondary outcomes. |

**Domain 3 judgment: Low risk**

**Domain 4 – Bias in measurement of the outcome**

| **Signalling question** | **Answer** | **Key evidence** |
| --- | --- | --- |
| 4.1 Outcome measurement appropriate? | **N** | ROSC defined objectively (palpable pulse/no compressions ≥ 20 min). |
| 4.2 Measurement differ by group? | **N** | Same pre-hospital procedures for both arms. |
| 4.3 Assessors aware of assignment? | **Y** | Treating clinicians (who recorded ROSC) were aware of group. |
| 4.4 Could awareness influence measurement? | **PN** | ROSC is a hard, objective endpoint; unlikely to be affected by awareness. |

**Domain 4 judgment: Low risk**

**Domain 5 – Bias in selection of the reported result**

| **Signalling question** | **Answer** | **Key evidence** |
| --- | --- | --- |
| 5.2 Result selected from multiple measurements? | **N** | Single definition/time-point for ROSC. |
| 5.3 Result selected from multiple analyses? | **N** | Only the pre-specified comparison reported. |
| 5.1 Analysis plan pre-specified? | **PY** | Protocol/SAP publicly available with article (dated but timing vs. database lock not explicit). |

**Domain 5 judgment: Low risk**

**Overall risk-of-bias judgment: Low risk**

All five domains were judged Low risk; the study provides a robust, low-bias estimate of the effect of intraosseous versus intravenous access on sustained ROSC in out-of-hospital cardiac arrest.

## Study 50

**Risk-of-Bias Assessment (RoB 2.0) for SAVE-MORE Trial** Primary outcome assessed: distribution of 11-point WHO Clinical Progression Scale (WHO-CPS) scores on day 28

**DOMAIN 1 – Risk of Bias arising from the Randomization Process**

| **Signalling question** | **Judgment** | **Reasoning** |
| --- | --- | --- |
| **1.1 Was the allocation sequence random?** | **PY** | “Patients with suPAR ≥ 6 ng ml⁻¹ were **electronically 1:2 randomized** into treatment with placebo or anakinra using four randomization strata.” (The term “electronically randomized” strongly implies a computer-generated sequence, but the method is not explicitly described.) |
| **1.2 Was the allocation sequence concealed until participants were enrolled and assigned?** | **Y** | Trial described as **“prospective, double-blind randomized”** , and drugs were prepared by an **unblinded pharmacist** while administered by a **blinded study nurse** —consistent with central concealment. |
| **1.3 Did baseline differences suggest a problem with randomization?** | **N** | “Baseline characteristics … were **similar between the two treatment arms** (Table 1).” |

**Domain 1 Judgment: Low risk** *Logic*: 1.2 = Y → consider 1.1 (PY acceptable) → 1.3 = N → **Low risk**.

**DOMAIN 2 – Risk of Bias due to Deviations from Intended Interventions (Effect of Assignment)**

| **Signalling question** | **Judgment** | **Reasoning** |
| --- | --- | --- |
| **2.1 Were participants aware of their assigned intervention?** | **N** | Trial was **double-blind**. |
| **2.2 Were carers/personnel aware of intervention?** | **N** | Same double-blind design; administration by blinded nurse. |
| **2.3–2.5 Deviations arising from awareness** | *Not triggered* | (2.1 & 2.2 = N) |
| **2.6 Was an appropriate analysis used (effect of assignment)?** | **Y** | “**Data were analyzed for the ITT population.**” |
| **2.7 Bias from inappropriate analysis** | *Not triggered* |  |

**Domain 2 Judgment: Low risk** *Logic*: Part 1 Low (blinding); Part 2 Low (ITT) → **Low risk**.

**DOMAIN 3 – Risk of Bias due to Missing Outcome Data**

| **Signalling question** | **Judgment** | **Reasoning** |
| --- | --- | --- |
| **3.1 Were outcome data available for nearly all participants?** | **Y** | Only **1/594 participants was lost to follow-up**. (≥ 99 % complete) |

**Domain 3 Judgment: Low risk** *Logic*: 3.1 = Y → **Low risk**.

**DOMAIN 4 – Risk of Bias in Measurement of the Outcome**

| **Signalling question** | **Judgment** | **Reasoning** |
| --- | --- | --- |
| **4.1 Was the method of measuring the outcome inappropriate?** | **N** | WHO-CPS is an established, validated ordinal scale. |
| **4.2 Could measurement differ between groups?** | **N** | Same scale and visit schedule used for both groups. |
| **4.3 Were outcome assessors aware of intervention?** | **N** | “Data were captured … by a **physician team blinded to the allocation group**.” |

**Domain 4 Judgment: Low risk** *Logic*: 4.1 = N, 4.2 = N, 4.3 = N → **Low risk**.

**DOMAIN 5 – Risk of Bias in Selection of the Reported Result**

| **Signalling question** | **Judgment** | **Reasoning** |
| --- | --- | --- |
| **5.2 Result selected from multiple measurements?** | **N** | Only one prespecified measurement/time-point (WHO-CPS day 28) reported as primary outcome. |
| **5.3 Result selected from multiple analyses?** | **N** | Primary analysis follows protocol; no unexplained alternative analyses presented. |
| **(5.1) Was analysis pre-specified?** | **Y** | “The **full SAP was developed … before database lock; no amendment was needed.**” |

**Domain 5 Judgment: Low risk** *Logic*: 5.2 = N & 5.3 = N → evaluate 5.1 (Y) → **Low risk**.

**Overall Risk-of-Bias Judgment: Low risk**

*All five domains were assessed as Low risk, indicating a robust trial design and conduct with minimal concern for bias influencing the primary outcome.*
