# Supplementary material for: Evaluating large language model performance in Risk of Bias assessments: A cross-sectional validation study
Source: PLoS One. 2026 Jul 9;21(7):e0353155. doi: 10.1371/journal.pone.0353155 (PMC13349098; doi:10.1371/journal.pone.0353155)
Supplement: S2. File — Completed TRIPOD-LLM checklist for this study. (DOCX) [file pone.0353155.s002.docx]

**TRIPOD-LLM Checklist (Filled)**

Manuscript: Evaluating Large Language Model Performance in Risk of Bias Assessments: A Cross-Sectional Validation Study

| **Section** | **Item** | **Checklist item** | **Page(s) / Location in manuscript** | **Notes (brief)** |
| --- | --- | --- | --- | --- |
| Title | 1 | Identify the study as developing, fine-tuning, and/or evaluating the performance of an LLM, specifying the task, the target population, and the outcome to be predicted. | p. 1 (Title page) | LLM evaluation; task = RoB 2.0; population = RCT reports; outcome = RoB judgments. |
| Abstract | 2 | See TRIPOD-LLM for Abstracts. | p. 2 (Abstract) | Abstract reports objective, design, sample size (50 RCTs), comparisons, and key metrics. |
| Introduction – Background | 3a | Explain the healthcare context/use case and rationale for developing or evaluating the LLM, including references to existing approaches and models. | pp. 4–5 (Introduction) | Context: systematic review RoB assessment; rationale and prior work cited. |
| Introduction – Background | 3b | Describe the target population and the intended use of the LLM in the context of the care pathway, including its intended users in current gold standard practices. | pp. 4–5 (Introduction) | Target users: systematic reviewers/methodologists; intended use as decision-support for RoB assessment. |
| Introduction – Objectives | 4 | Specify the study objectives, including whether the study describes the initial development, fine-tuning, or validation of an LLM (or multiple stages). | pp. 4–5 (Introduction) | Objective: evaluation/validation of an existing closed-source LLM (ChatGPT-o3). |
| Methods – Data | 5a | Describe the sources of data separately for the training, tuning, and/or evaluation datasets and the rationale for using these data. | p. 5 (Study Design and Search Strategy) | Evaluation dataset from meta-analyses (2020–2025) and one RCT per meta-analysis; model training/tuning data not available (closed-source). |
| Methods – Data | 5b | Describe the relevant data points and provide a quantitative and qualitative description of their distribution and other relevant descriptors of the dataset. | pp. 5–6 (Search Strategy; Data Collection); Table 1 p. 23 | Describes 50 RCTs across subspecialties; includes RoB domain labels (Low/Some/High) and overall judgment. |
| Methods – Data | 5c | State the date of the oldest and newest item of text used in development and in the evaluation datasets. | p. 6 (Study Design and Search Strategy) | Evaluation texts: meta-analyses 2020–2025; RCT publications 1998–2025; inference window Apr 20–May 20, 2025. |
| Methods – Data | 5d | Describe any data pre-processing and quality checking, including whether similar across corpora/institutions/groups. | p. 6 (Data Collection and RoB Assessment) | Full-text RCT PDFs uploaded; new chat per trial; registry/protocol retrieval described; no additional preprocessing beyond extraction. |
| Methods – Data | 5e | Describe how missing and imbalanced data were handled and provide reasons for omitting any data. | pp. 6–7 (Data Collection; Statistical Analysis) | Imbalance addressed via agreement statistics robust to prevalence (Gwet’s AC2); missing ancillary documents handled by using available sources; no trial exclusions after selection. |
| Methods – Analytical Methods | 6a | Report the LLM name, version, and last date of training. | p. 6 (Data Collection); refs p. 20 | LLM: ChatGPT-o3 (accessed via ChatGPT web app). Last training date not publicly disclosed; inference date window reported. |
| Methods – Analytical Methods | 6b | Report details of LLM development process (architecture, training, fine-tuning, alignment strategy/goals). | p. 15 (Limitations) and refs p. 20 | Closed-source; developer details limited. Manuscript cites developer information where available; otherwise noted as not available. |
| Methods – Analytical Methods | 6c | Report details of how text was generated, including prompt engineering and inference settings. | p. 6 (Data Collection and RoB Assessment) | Prompt-based RoB 2.0 assessment; default parameters; new chat per RCT; browsing enabled; PDF uploads; structured template used (supplement). |
| Methods – Analytical Methods | 6d | Specify the initial and post-processed output of the LLM. | pp. 6–7 (Data Collection; Statistical Analysis) | Initial output: domain-level and overall RoB judgments with rationale; post-processing: extraction of categorical labels for analyses. |
| Methods – Analytical Methods | 6e | Provide details and rationale for any classification and how probabilities/thresholds identified. | p. 7 (Statistical Analysis) | Ordinal RoB labels analyzed; additional dichotomizations for 'High-risk' vs 'Not High-risk' and 'Low-risk' vs 'Not Low-risk'. |
| Methods – LLM Output | 7a | Include metrics that capture the quality of generative outputs compared to gold standards. | pp. 7–12 (Statistical Analysis; Results); Tables 2–3 p. 26–27 | Agreement (weighted kappa, Gwet’s AC2), diagnostic metrics (sensitivity/specificity/balanced accuracy) vs human panel and OSRA. |
| Methods – LLM Output | 7b | Report outcome metrics' relevance to downstream deployment and correlation to human evaluation, where applicable. | pp. 14–15 (Discussion) | Discusses suitability for workflow support; balanced accuracy/specificity emphasized for screening/triage use cases. |
| Methods – LLM Output | 7c | Define the outcome, how predictions were calculated, inference date, and evaluation metrics. | pp. 6–7 (Data Collection; Statistical Analysis) | Outcome: RoB 2.0 domain and overall judgments; predictions from LLM output categories; inference dates stated; metrics defined. |
| Methods – LLM Output | 7d | If subjective, describe assessor qualifications, instructions, demographics, and inter-assessor agreement. | pp. 6–7 (Data Collection); Table 2 p. 26 | Three masked physician reviewers; consensus and adjudication described; interrater agreement reported. |
| Methods – LLM Output | 7e | Specify how performance was compared to other LLMs, humans, and other benchmarks/standards. | pp. 8–12 (Results); Tables 2–3 p. 26–27 | Comparisons: LLM vs masked panel; OSRA vs masked panel; LLM vs OSRA. |
| Methods – Annotation | 8a | If annotation was done, report how text was labeled, including guidelines with examples. | p. 6 (Data Collection and RoB Assessment) | Human panel applied RoB 2.0; LLM instructed using RoB 2.0 guidance and structured template (supplement). |
| Methods – Annotation | 8b | Report how many annotators labeled dataset(s), overlap, and inter-annotator agreement. | pp. 6–7 (Data Collection; Statistical Analysis); Table 2 p. 26 | Three independent raters; full overlap across all 50 RCTs; pairwise agreement and summary measures reported. |
| Methods – Annotation | 8c | Provide background/experience of annotators or models involved in labeling. | p. 1 (Authors/credentials); p. 6 (Data Collection) | Annotators are study authors with medical training; roles and masking described. |
| Methods – Prompting | 9a | Provide details on processes used during prompt design, curation, and selection. | p. 6 (Data Collection) and supplement | Prompt integrates RoB 2.0 domains and structured output template; standardized across trials. |
| Methods – Prompting | 9b | Report what data were used to develop the prompts. | p. 6 (Data Collection); refs p. 20 | RoB 2.0 guidance and prior template (Lai et al.) used to develop prompt. |
| Methods – Summarization | 10 | Describe any preprocessing of the data before summarization. | N/A | Not a summarization/simplification study. |
| Methods – Instruction tuning/alignment | 11 | If instruction tuning/alignment strategies were used, report instructions, data, interface, and evaluator characteristics. | N/A (existing closed-source LLM) | No tuning/alignment performed by study team. |
| Methods – Compute | 12 | Report compute, or proxies thereof, required to carry out methods. | p. 15 (Limitations) | Compute/cost not quantified; inference done in 50 independent sessions during Apr–May 2025. |
| Methods – Ethical Approval | 13 | Name IRB/ethics committee and consent/waiver. | p. 6 (Study Design) | No human participants; IRB not required; Declaration of Helsinki noted. |
| Open Science | 14a | Give funding source and role of funders. | p. 18 (Acknowledgements) | Funding stated; funder role described. |
| Open Science | 14b | Declare conflicts of interest and financial disclosures. | p. 18 (Acknowledgements) | COI/disclosures stated. |
| Open Science | 14c | Indicate where protocol can be accessed or state protocol not prepared. | p. 5 (Study Design) | Not pre-registered; protocol not prepared/available. |
| Open Science | 14d | Provide registration information or state not registered. | p. 5 (Study Design) | Study not registered. |
| Open Science | 14e | Provide details of availability of study data. | p. 23 (Table 1 is the data source for this study) | Table 1 is the data |
| Open Science | 14f | Provide details of availability of code to reproduce results. | Code was uploaded in initial file submission | Code was uploaded in initial file submission |
| Public Involvement | 15 | Provide details of patient/public involvement or state no involvement. | p. 18 (Acknowledgements) | No patient/public involvement stated. |
| Results – Participants | 16a | Describe flow of text/EHR/patient data through study. | pp. 5–6 (Search Strategy; Study Design) | Flow described: meta-analyses identified and screened; one RCT randomly selected from each. |
| Results – Participants | 16b | Report characteristics overall and by data source/setting/splits. | p. 6 (Study Design); Table 1 p. 23 | RCT date range and diversity described; full domain distributions provided in Table 1. |
| Results – Participants | 16c | For evaluation with clinical outcomes, compare clinical variable distributions between development and evaluation data. | N/A | No clinical outcome prediction. |
| Results – Participants | 16d | When using patient/EHR data, specify number of participants and outcome events. | N/A | No patient/EHR data; unit of analysis = RCT report. |
| Results – Performance | 17 | Report LLM performance according to pre-specified metrics and/or human evaluation. | pp. 8–12 (Results); Tables 2–3 p. 26–27 | All primary performance metrics reported with confidence intervals and pairwise comparisons. |
| Results – LLM Updating | 18 | If applicable, report results from LLM updating. | N/A | No LLM updating performed. |
| Discussion – Interpretation | 19a | Overall interpretation of main results, including fairness issues. | pp. 12–15 (Discussion) | Interpretation in context of prior RoB-LLM studies; notes generalizability considerations. |
| Discussion – Limitations | 19b | Discuss limitations and effects on bias/uncertainty/generalizability. | pp. 14–15 (Discussion) | Limitations include prompt dependence, browsing, leakage risk, and lack of time-savings analysis. |
| Discussion – Usability | 19c | Challenges in using data for task/context with reference to representation, missingness, harmonization, bias. | pp. 14–15 (Discussion) | Discusses trial reporting variability, domain-specific challenges, and missing protocol/registry issues. |
| Discussion – Usability | 19d | Define intended use including input, end-user, autonomy/oversight. | pp. 14–15 (Discussion) | Human-in-the-loop decision support; input = RCT PDF (+ registry/protocol); oversight required. |
| Discussion – Usability | 19e | How poor quality/unavailable input data should be assessed/handled in implementation. | p. 15 (Discussion/Limitations) | Notes need for human verification; recommends handling missing protocols and PDF quality issues. |
| Discussion – Usability | 19f | Whether users must interact and what expertise required. | p. 15 (Discussion) | Requires RoB-trained user to verify/override judgments; intended as assistive tool. |
| Discussion – Next steps | 19g | Next steps for future research with view to applicability/generalizability. | p. 15 (Discussion) | Calls for larger datasets, multiple models, prompt sensitivity, and workflow impact studies. |
